# The origins and spread of domestic horses from the Western Eurasian steppes

**DOI:** 10.1038/s41586-021-04018-9

**Published:** 2021-10-20

**Authors:** Pablo Librado, Naveed Khan, Antoine Fages, Mariya A. Kusliy, Tomasz Suchan, Laure Tonasso-Calvière, Stéphanie Schiavinato, Duha Alioglu, Aurore Fromentier, Aude Perdereau, Jean-Marc Aury, Charleen Gaunitz, Lorelei Chauvey, Andaine Seguin-Orlando, Clio Der Sarkissian, John Southon, Beth Shapiro, Alexey A. Tishkin, Alexey A. Kovalev, Saleh Alquraishi, Ahmed H. Alfarhan, Khaled A. S. Al-Rasheid, Timo Seregély, Lutz Klassen, Rune Iversen, Olivier Bignon-Lau, Pierre Bodu, Monique Olive, Jean-Christophe Castel, Myriam Boudadi-Maligne, Nadir Alvarez, Mietje Germonpré, Magdalena Moskal-del Hoyo, Jarosław Wilczyński, Sylwia Pospuła, Anna Lasota-Kuś, Krzysztof Tunia, Marek Nowak, Eve Rannamäe, Urmas Saarma, Gennady Boeskorov, Lembi Lōugas, René Kyselý, Lubomír Peške, Adrian Bălășescu, Valentin Dumitrașcu, Roxana Dobrescu, Daniel Gerber, Viktória Kiss, Anna Szécsényi-Nagy, Balázs G. Mende, Zsolt Gallina, Krisztina Somogyi, Gabriella Kulcsár, Erika Gál, Robin Bendrey, Morten E. Allentoft, Ghenadie Sirbu, Valentin Dergachev, Henry Shephard, Noémie Tomadini, Sandrine Grouard, Aleksei Kasparov, Alexander E. Basilyan, Mikhail A. Anisimov, Pavel A. Nikolskiy, Elena Y. Pavlova, Vladimir Pitulko, Gottfried Brem, Barbara Wallner, Christoph Schwall, Marcel Keller, Keiko Kitagawa, Alexander N. Bessudnov, Alexander Bessudnov, William Taylor, Jérome Magail, Jamiyan-Ombo Gantulga, Jamsranjav Bayarsaikhan, Diimaajav Erdenebaatar, Kubatbeek Tabaldiev, Enkhbayar Mijiddorj, Bazartseren Boldgiv, Turbat Tsagaan, Mélanie Pruvost, Sandra Olsen, Cheryl A. Makarewicz, Silvia Valenzuela Lamas, Silvia Albizuri Canadell, Ariadna Nieto Espinet, Ma Pilar Iborra, Jaime Lira Garrido, Esther Rodríguez González, Sebastián Celestino, Carmen Olària, Juan Luis Arsuaga, Nadiia Kotova, Alexander Pryor, Pam Crabtree, Rinat Zhumatayev, Abdesh Toleubaev, Nina L. Morgunova, Tatiana Kuznetsova, David Lordkipanize, Matilde Marzullo, Ornella Prato, Giovanna Bagnasco Gianni, Umberto Tecchiati, Benoit Clavel, Sébastien Lepetz, Hossein Davoudi, Marjan Mashkour, Natalia Ya. Berezina, Philipp W. Stockhammer, Johannes Krause, Wolfgang Haak, Arturo Morales-Muñiz, Norbert Benecke, Michael Hofreiter, Arne Ludwig, Alexander S. Graphodatsky, Joris Peters, Kirill Yu. Kiryushin, Tumur-Ochir Iderkhangai, Nikolay A. Bokovenko, Sergey K. Vasiliev, Nikolai N. Seregin, Konstantin V. Chugunov, Natalya A. Plasteeva, Gennady F. Baryshnikov, Ekaterina Petrova, Mikhail Sablin, Elina Ananyevskaya, Andrey Logvin, Irina Shevnina, Victor Logvin, Saule Kalieva, Valeriy Loman, Igor Kukushkin, Ilya Merz, Victor Merz, Sergazy Sakenov, Victor Varfolomeyev, Emma Usmanova, Viktor Zaibert, Benjamin Arbuckle, Andrey B. Belinskiy, Alexej Kalmykov, Sabine Reinhold, Svend Hansen, Aleksandr I. Yudin, Alekandr A. Vybornov, Andrey Epimakhov, Natalia S. Berezina, Natalia Roslyakova, Pavel A. Kosintsev, Pavel F. Kuznetsov, David Anthony, Guus J. Kroonen, Kristian Kristiansen, Patrick Wincker, Alan Outram, Ludovic Orlando

**Affiliations:** 1 Centre d’Anthropobiologie et de Génomique de Toulouse, Université Paul Sabatier, Toulouse, France; 2 Department of the Diversity and Evolution of Genomes, Institute of Molecular and Cellular Biology SB RAS, Novosibirsk, Russia; 3 W. Szafer Institute of Botany, Polish Academy of Sciences, Kraków, Poland; 4 Genoscope, Institut de biologie François-Jacob, Commissariat à l’Energie Atomique (CEA), Université Paris-Saclay, Evry, France; 5 Génomique Métabolique, Genoscope, Institut de biologie François Jacob, CEA, CNRS, Université d’Evry, Université Paris-Saclay, Evry, France; 6 Earth System Science Department, University of California, Irvine, Irvine, CA USA; 7 Department of Ecology and Evolutionary Biology, University of California, Santa Cruz, Santa Cruz, CA USA; 8 Howard Hughes Medical Institute, University of California, Santa Cruz, Santa Cruz, CA USA; 9 Department of Archaeology, Ethnography and Museology, Altai State University, Barnaul, Russia; 10 Department of Archaeological Heritage Preservation, Institute of Archaeology of the Russian Academy of Sciences, Moscow, Russia; 11 Zoology Department, College of Science, King Saud University, Riyadh, Saudi Arabia; 12 Institute for Archaeology, Heritage Conservation Studies and Art History, University of Bamberg, Bamberg, Germany; 13 Museum Østjylland, Randers, Denmark; 14 Saxo Institute, section of Archaeology, University of Copenhagen, Copenhagen, Denmark; 15 ArScAn-UMR 7041, Equipe Ethnologie préhistorique, CNRS, MSH-Mondes, Nanterre Cedex, France; 16 Muséum d’histoire naturelle, Secteur des Vertébrés, Geneva, Switzerland; 17 UMR 5199 De la Préhistoire à l’Actuel : Culture, Environnement et Anthropologie (PACEA), CNRS, Université de Bordeaux, Pessac Cedex, France; 18 Geneva Natural History Museum, Geneva, Switzerland; 19 Department of Genetics and Evolution, University of Geneva, Geneva, Switzerland; 20 OD Earth & History of Life, Royal Belgian Institute of Natural Sciences, Brussels, Belgium; 21 Institute of Systematics and Evolution of Animals, Polish Academy of Sciences, Kraków, Poland; 22 Institute of Archaeology and Ethnology Polish Academy of Sciences, Kraków, Poland; 23 Institute of Archaeology, Jagiellonian University, Kraków, Poland; 24 Department of Archaeology, Institute of History and Archaeology, Tartu, Estonia; 25 Department of Zoology, Institute of Ecology and Earth Sciences, University of Tartu, Tartu, Estonia; 26 Diamond and Precious Metals Geology Institute, SB RAS, Yakutsk, Russia; 27 Archaeological Research Collection, Tallinn University, Tallinn, Estonia; 28 Department of Natural Sciences and Archaeometry, Institute of Archaeology of the Czech Academy of Sciences, Prague, Czechia; 29 Prague, Czechia; 30 Vasile Pârvan Institute of Archaeology, Department of Bioarchaeology, Romanian Academy, Bucharest, Romania; 31 Institute of Archaeogenomics, Research Centre for the Humanities, Eötvös Loránd Research Network, Budapest, Hungary; 32 Department of Genetics, Eötvös Loránd University, Budapest, Hungary; 33 Institute of Archaeology, Research Centre for the Humanities, Eötvös Loránd Research Network, Budapest, Hungary; 34 Ásatárs Ltd., Kecskemét, Hungary; 35 Rippl-Rónai Municipal Museum with Country Scope, Kaposvár, Hungary; 36 School of History, Classics and Archaeology, University of Edinburgh, Old Medical School, Edinburgh, UK; 37 Trace and Environmental DNA (TrEnD) Lab, School of Molecular and Life Sciences, Curtin University, Perth, Western Australia Australia; 38 Lundbeck Foundation GeoGenetics Centre, GLOBE Institute, University of Copenhagen, Copenhagen, Denmark; 39 Department of Academic Management, Academy of Science of Moldova, Chișinău, Republic of Moldova; 40 Center of Archaeology, Institute of Cultural Heritage, Academy of Science of Moldova, Chișinău, Republic of Moldova; 41 Archaeological Institute of America, Boston, MA USA; 42 Centre National de Recherche Scientifique, Muséum national d’Histoire naturelle, Archéozoologie, Archéobotanique (AASPE), CP 56, Paris, France; 43 Institute for the History of Material Culture, Russian Academy of Sciences (IHMC RAS), St Petersburg, Russia; 44 Geological Institute, Russian Academy of Sciences, Moscow, Russia; 45 Arctic and Antarctic Research Institute, St Petersburg, Russia; 46 Institute of Animal Breeding and Genetics, University of Veterinary Medicine Vienna, Vienna, Austria; 47 Department of Prehistory and Western Asian/Northeast African Archaeology, Austrian Archaeological Institute, Austrian Academy of Sciences, Vienna, Austria; 48 Estonian Biocentre, Institute of Genomics, University of Tartu, Tartu, Estonia; 49 Department of Archaeogenetics, Max Planck Institute for the Science of Human History, Jena, Germany; 50 SFB 1070 Resource Cultures, University of Tübingen, Tübingen, Germany; 51 Department of Early Prehistory and Quaternary Ecology, University of Tübingen, Tübingen, Germany; 52 UMR 7194 Muséum National d’Histoire Naturelle, CNRS, UPVD, Paris, France; 53 Semenov-Tyan-Shanskii Lipetsk State Pedagogical University, Lipetsk, Russia; 54 Museum of Natural History, University of Colorado-Boulder, Boulder, CO USA; 55 Musée d’Anthropologie préhistorique de Monaco, Monaco, Monaco; 56 Institute of Archaeology, Mongolian Academy of Sciences, Ulaanbaatar, Mongolia; 57 Department of Archaeology, Max Planck Institute for the Science of Human History, Jena, Germany; 58 Chinggis Khaan Museum, Ulaanbaatar, Mongolia; 59 Department of Archaeology, Ulaanbaatar State University, Ulaanbaatar, Mongolia; 60 Department of History, Kyrgyz-Turkish Manas University, Bishkek, Kyrgyzstan; 61 Department of Biology, National University of Mongolia, Ulaanbaatar, Mongolia; 62 Division of Archaeology, Biodiversity Institute, University of Kansas, Lawrence, KS USA; 63 Institute for Prehistoric and Protohistoric Archaeology, Kiel University, Kiel, Germany; 64 ROOTS Excellence Cluster, Kiel University, Kiel, Germany; 65 Archaeology of Social Dynamics, Institució Milà i Fontanals d’Humanitats, Consejo Superior de Investigaciones Científicas (IMF-CSIC), Barcelona, Spain; 66 Departament d’Història i Arqueologia–SERP, Universitat de Barcelona, Barcelona, Spain; 67 Grup d’Investigació Prehistòrica, Universitat de Lleida, PID2019-110022GB-I00, Lleida, Spain; 68 Valencia, Spain; 69 Departamento de Medicina Animal, Facultad de Veterinaria, Universidad de Extremadura, Cáceres, Spain; 70 Centro Mixto UCM-ISCIII de Evolución y Comportamiento Humanos, Madrid, Spain; 71 Instituto de Arqueología (CSIC–Junta de Extremadura), Mérida, Spain; 72 Laboratori d’Arqueologia Prehistòrica, Universitat Jaume I, Castelló de la Plana, Spain; 73 Departamento de Geodinámica, Estratigrafía y Paleontología, Facultad de Ciencias Geológicas, Universidad Complutense de Madrid, Madrid, Spain; 74 Department of Eneolithic and Bronze Age, Institute of Archaeology National Academy of Sciences of Ukraine, Kyiv, Ukraine; 75 Department of Archaeology, University of Exeter, Exeter, UK; 76 Center for the Study of Human Origins, Anthropology Department, New York University, New York, NY USA; 77 Department of Archaeology, Ethnology and Museology, Al Farabi Kazakh National University, Almaty, Kazakhstan; 78 Scientific Research Department, Orenburg State Pedagogical University, Orenburg, Russia; 79 Department of paleontology, Faculty of Geology, Moscow State University, Moscow, Russia; 80 Institute of Geology and Petroleum Technologies, Kazan Federal University, Kazan, Russia; 81 Georgian National Museum, Tbilisi, Georgia; 82 Tbilisi State University, Tbilisi, Georgia; 83 Università degli Studi di Milano, Dipartimento di Beni Culturali e Ambientali, Milan, Italy; 84 University of Tehran, Central Laboratory, Bioarchaeology Laboratory, Archaeozoology Section, Tehran, Iran; 85 Research Institute and Museum of Anthropology, Lomonosov Moscow State University, Moscow, Russia; 86 Department of Archaeogenetics, Max Planck Institute for Evolutionary Anthropology, Leipzig, Germany; 87 Institute for Pre- and Protohistoric Archaeology and Archaeology of the Roman Provinces, Ludwig Maximilian University, Munich, Munich, Germany; 88 School of Biological Sciences, The University of Adelaide, Adelaide, South Australia Australia; 89 Department of Biology, Universidad Autónoma de Madrid, Madrid, Spain; 90 Eurasia Department of the German Archaeological Institute, Berlin, Germany; 91 Evolutionary Adaptive Genomics, Institute of Biochemistry and Biology, Faculty of Mathematics and Science, University of Potsdam, Potsdam, Germany; 92 Department of Evolutionary Genetics, Leibniz-Institute for Zoo and Wildlife Research, Berlin, Germany; 93 Albrecht Daniel Thaer-Institute, Faculty of Life Sciences, Humboldt University Berlin, Berlin, Germany; 94 ArchaeoBioCenter and Institute of Palaeoanatomy, Domestication Research and the History of Veterinary Medicine, LMU Munich, Munich, Germany; 95 SNSB, State Collection of Anthropology and Palaeoanatomy, Munich, Germany; 96 ArchaeoZOOlogy in Siberia and Central Asia—ZooSCAn International Research Laboratory, Institute of Archeology and Ethnography of the Siberian Branch of the RAS, Novosibirsk, Russia; 97 Department of Eastern European and Siberian Archaeology, State Hermitage Museum, St Petersburg, Russia; 98 Paleoecology Laboratory, Institute of Plant and Animal Ecology, Ural Branch of the Russian Academy of Sciences, Ekaterinburg, Russia; 99 Zoological Institute, Russian Academy of Sciences, St Petersburg, Russia; 100 Department of Archaeology, History Faculty, Vilnius University, Vilnius, Lithuania; 101 Laboratory for Archaeological Research, Faculty of History and Law, Kostanay State University, Kostanay, Kazakhstan; 102 Department of History and Archaeology, Surgut Governmental University, Surgut, Russia; 103 Saryarka Archaeological Institute, Buketov Karaganda University, Karaganda, Kazakhstan; 104 Toraighyrov University, Joint Research Center for Archeological Studies, Pavlodar, Kazakhstan; 105 Faculty of History, L. N. Gumilev Eurasian National University, Nur-Sultan, Kazakhstan; 106 Institute of Archaeology and Steppe Civilizations, Al-Farabi Kazakh National University, Almaty, Kazakhstan; 107 Department of Anthropology, Alumni Building, University of North Carolina at Chapel Hill, Chapel Hill, NC USA; 108 Nasledie Cultural Heritage Unit, Stavropol, Russia; 109 Research Center for the Preservation of Cultural Heritage, Saratov, Russia; 110 Department of Russian History and Archaeology, Samara State University of Social Sciences and Education, Samara, Russia; 111 Russian and Foreign History Department, South Ural State University, Chelyabinsk, Russia; 112 South Ural Department, Institute of History and Archaeology, Ural Branch of the Russian Academy of Sciences, Ekaterinburg, Russia; 113 Archaeological School, Chuvash State Institute of Humanities, Cheboksary, Russia; 114 Department of History of the Institute of Humanities, Ural Federal University, Ekaterinburg, Russia; 115 Department of Human Evolutionary Biology, Harvard University, Cambridge, MA USA; 116 Anthropology Faculty, Hartwick College, Oneonta NY, USA; 117 Department of Nordic Studies and Linguistics, University of Copenhagen, Copenhagen, Denmark; 118 Leiden University Center for Linguistics, Leiden University, Leiden, The Netherlands; 119 Department of Historical Studies, University of Gothenburg, Gothenburg, Sweden; 120 Present Address: Lundbeck Foundation GeoGenetics Centre, Copenhagen, Denmark; 121 Department of Biotechnology, Abdul Wali Khan University, Mardan, Pakistan

**Keywords:** Evolutionary genetics, Population genetics, Population genetics

## Abstract

Domestication of horses fundamentally transformed long-range mobility and warfare^1^. However, modern domesticated breeds do not descend from the earliest domestic horse lineage associated with archaeological evidence of bridling, milking and corralling^2–4^ at Botai, Central Asia around 3500 bc^3^. Other longstanding candidate regions for horse domestication, such as Iberia^5^ and Anatolia^6^, have also recently been challenged. Thus, the genetic, geographic and temporal origins of modern domestic horses have remained unknown. Here we pinpoint the Western Eurasian steppes, especially the lower Volga-Don region, as the homeland of modern domestic horses. Furthermore, we map the population changes accompanying domestication from 273 ancient horse genomes. This reveals that modern domestic horses ultimately replaced almost all other local populations as they expanded rapidly across Eurasia from about 2000 bc, synchronously with equestrian material culture, including Sintashta spoke-wheeled chariots. We find that equestrianism involved strong selection for critical locomotor and behavioural adaptations at the *GSDMC* and *ZFPM1* genes. Our results reject the commonly held association^7^ between horseback riding and the massive expansion of Yamnaya steppe pastoralists into Europe around 3000 bc^8,9^ driving the spread of Indo-European languages^10^. This contrasts with the scenario in Asia where Indo-Iranian languages, chariots and horses spread together, following the early second millennium bc Sintashta culture^11,12^.

## Abstract (web summary)

Analysis of 273 ancient horse genomes reveals that modern domestic horses originated in the Western Eurasian steppes, especially the lower Volga-Don region.

## Main

**Fig. 1:**
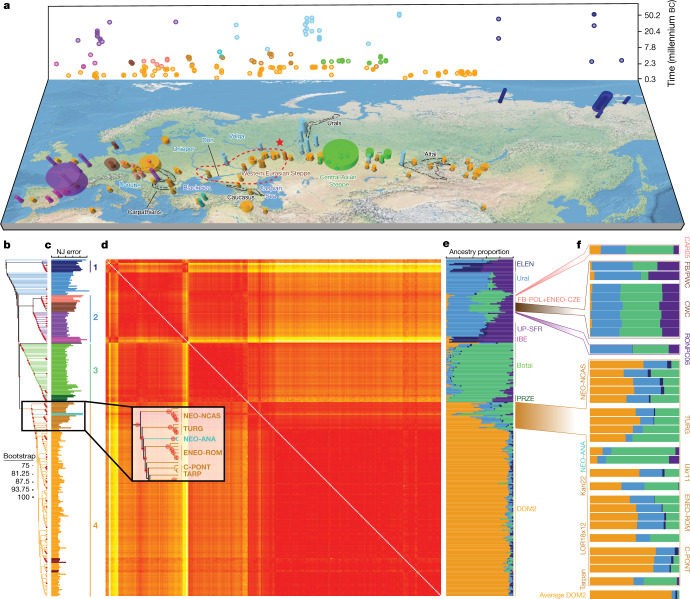
Ancient horse remains and their genomic affinities. **a**, Temporal and geographic sampling. The red star indicates the location of the two TURG horses (late Yamnaya context) showing genetic continuity with DOM2. The dashed line indicates the inferred homeland of DOM2 horses in the lower Volga-Don region. Colours refer to regions and/or time periods delineating genetically close horses. The radius of each cylinder is proportional to the number of samples analysed (for <10 specimens; radius constant above this), and the height refers to the time range covered. **b**, Neighbour-joining phylogenomic tree (100 bootstrap pseudo-replicates). Samples are coloured according to **a** and the main phylogenetic clusters are numbered from 1 to 4. **c**, Fold difference between neighbour-joining-based and raw pairwise genetic distances. **d**, Pairwise distance matrix of Struct-f4 genetic affinities between samples. Increasing genetic affinities are indicated by a yellow-to-red gradient. **e**, Struct-f4 ancestry component profiles. **f**, Ancestry profiles of selected key horse groups and samples. PRZE, Przewalski; UP-SFR, Upper Palaeolithic Southern France.

We gathered horse remains encompassing all suspected domestication centres, including Iberia, Anatolia and the steppes of Western Eurasia and Central Asia (Fig 1a). The sampling targeted previously under-represented time periods, with 201 radiocarbon dates spanning 44426 to 202 bc, and five beyond 50250 to 47950 bc (Supplementary Table 1).

**Extended Data Fig. 1:**
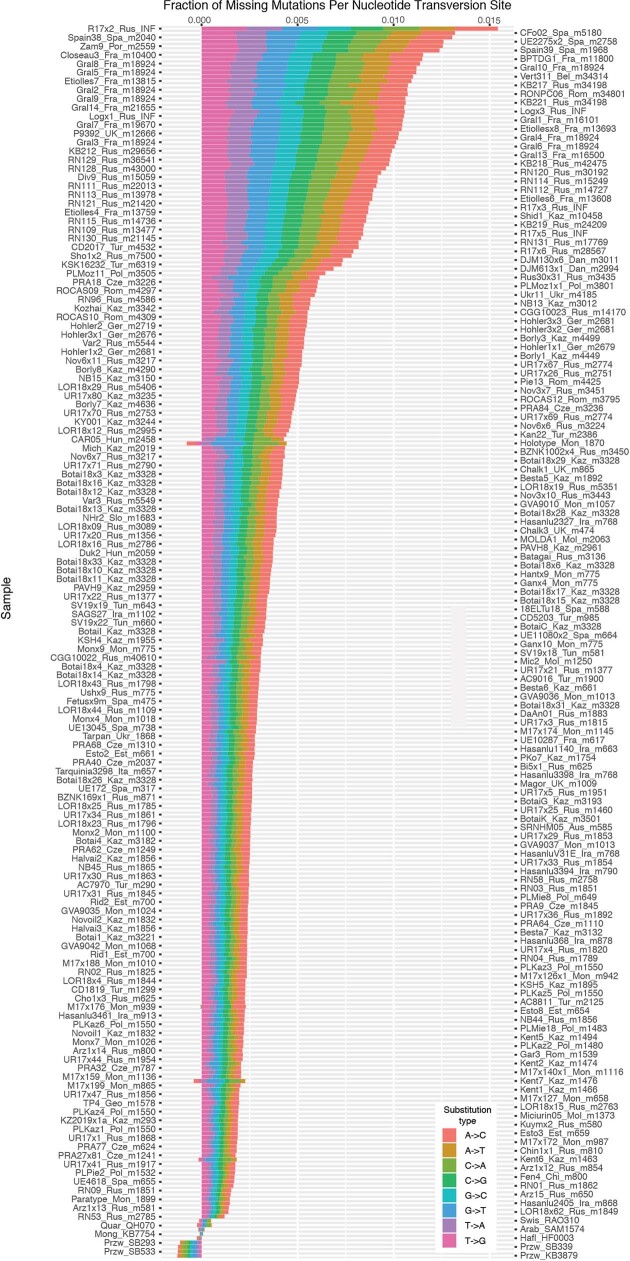
Proportion of missing derived mutations at sites representing nucleotide transversions. Proportions are provided relative to the genome of a modern Icelandic^89^ (P5782) horse (Spearman correlation coefficient between total transversion errors and time, R=−0.77 p-value =0).

The DNA quality enabled shotgun sequencing of 264 ancient genomes at 0.10× to 25.76× average coverage (239 genomes above 1× coverage), including 16 genomes for which further sequencing added to previously reported data. Enzymatic^13^ and computational removal of post mortem DNA damage produced high-quality data with derived mutations decreasing with sample age, as expected if mutations accumulate through time (Extended Data Fig. 1). We added ten published modern genomes, and nine ancient genomes characterized with consistent technology or covering relevant time periods and locations, to obtain the most extensive high-quality genome time series for horses.

## Pre-domestication population structure

**Extended Data Fig. 2:**
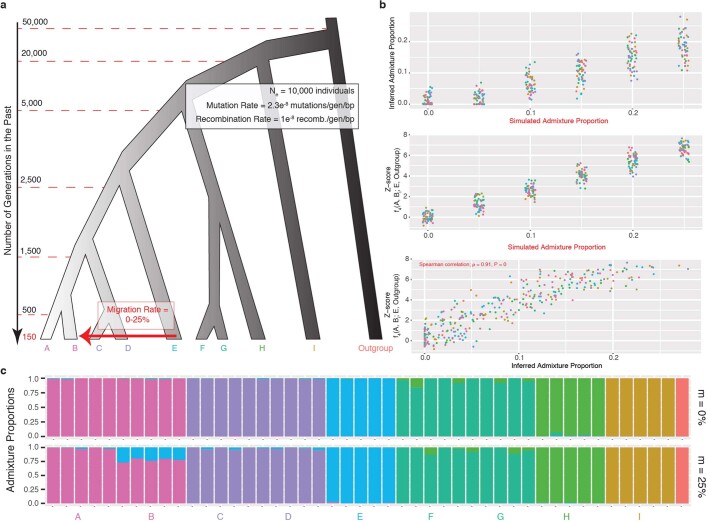
Struct-f4 validation. **a**, Simulated demographic model. A single migration pulse is assumed to have occurred 150 generations ago from population E into B. The magnitude of the migration represents 5% to 25% of the effective size of population B. The model was also simulated in the absence of migration (i.e. m=0%). Five individuals are simulated per population considered, except for the outgroup where only one individual was considered. **b**, Correlation of the expected levels of gene-flow with the predicted E-ancestry component in individuals *i* belonging to population B, as well as with the average Z-scores of the *f*_*4*_(A, B_*i*_; E, Outgroup) configurations, which reflects the stochasticity resulting from the simulations, prior to any inference. Each point represents a simulated individual. Colors indicate the 10 independent simulation replicates carried out. **c**, Predicted ancestry profiles in the absence (m=0%) and with gene flow (m=25% and K=7, as per the number of internal nodes immediately ancestral to the 10 extant populations).

Neighbour-joining phylogenomic inference revealed four geographically defined monophyletic groups (Fig 1b). These closely mirrored clusters identified using an extension of the Struct-f4 method^5^ (Fig 1d–f, Extended Data Fig. 2, Supplementary Methods), except for the Neolithic Anatolia group (NEO-ANA), where the tree-to-data goodness of fit suggested phylogenetic misplacement (Fig 1c, Supplementary Methods).

**Fig. 2:**
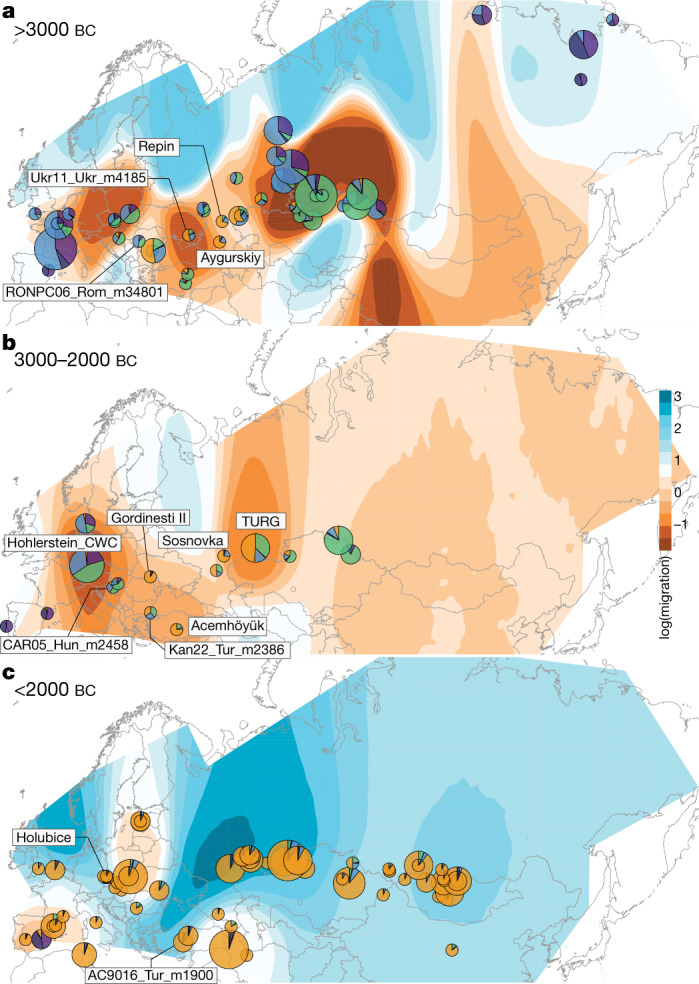
Horse geographic and genetic affinities. **a**–**c**, EEMS-predicted migration barriers^16^ and average ancestry components found in each archaeological site from before 3000 bc (**a**), during the third millennium bc (**b**) and after around 2000 bc (**c**). The size of the pie charts is proportional to the number of samples analysed in a given location (<10, constant above). Pie chart colours refer to *K* = 6 ancestry components, averaged per location. Regions inferred as geographic barriers are shown in shades of brown, and regions affected by migrations are shown in shades of blue. The base map was obtained from rworldmap^46^.

**Extended Data Fig. 3:**
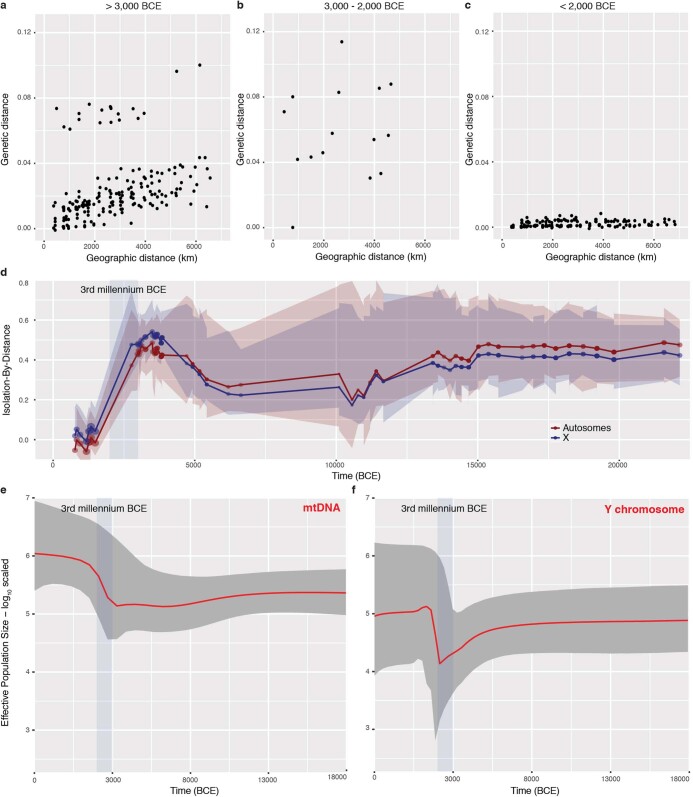
Mobility and demographic shifts. **a–c**, Correlation between observed pairwise genetic distances between demes as inferred by EEMS^16^ and Haversine geographic distances prior to ~3,000 BCE (**a**), during the third millennium BCE (**b**) and after ~2,000 BCE (**c**). **d**, Isolation-by-distance patterns through time inferred from autosomal (red) and X-chromosomal (blue) variation. **e–f**, Bayesian Skyline plots reconstructed from mtDNA (**e**) and Y-chromosomal variation (**f**). The third millennium BCE is highlighted in blue. The red line indicates the median of the 95% confidence range, shown in grey.

The most basal cluster included *Equus lenensis* (ELEN), a lineage identified in northeastern Siberia from the Late Pleistocene to the late fourth millennium bc^5,14,15^. A second group covered Europe, including Late Pleistocene Romania, Belgium, France and Britain, and the region from Spain to Scandinavia and Hungary, Czechia and Poland during the sixth-to-third millennium bc. The third cluster comprised the earliest known domestic horses from Botai and Przewalski’s horses, as previously reported^3^, and extended to the Altai and Southern Urals during the fifth-to-third millennium bc. Finally, modern domestic horses clustered within a group that became geographically widespread and prominent following about 2200 bc and during the second millennium bc (DOM2). This cluster appears genetically close to horses that lived in the Western Eurasia steppes (WE) but not further west than the Romanian lower Danube, south of the Carpathians, before and during the third millennium bc. Significant correlation between genetic and geographic distances, and inference of limited long-distance connectivity with estimated effective migration surface^16^ (EEMS), confirmed the strong geographic differentiation of horse populations before about 3000 bc (Fig 2a, Extended Data Fig. 3a).

**Fig. 3:**
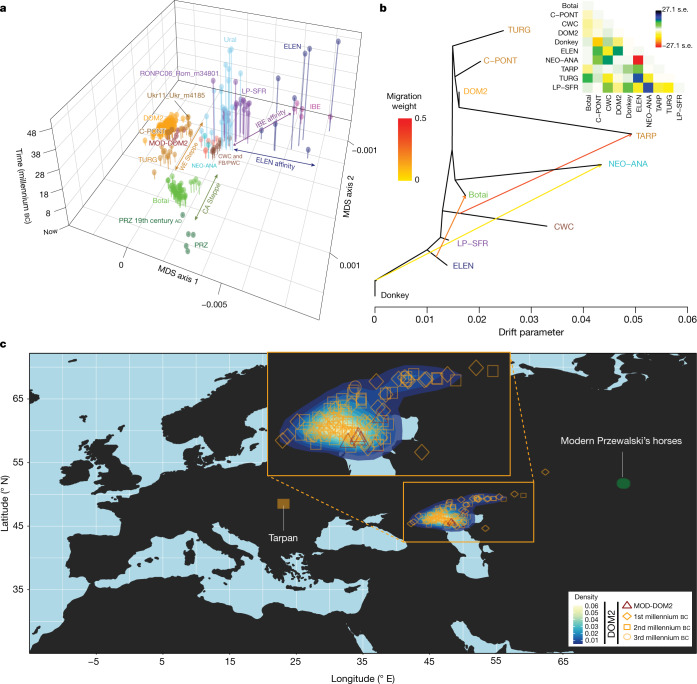
Population genetic affinities, evolutionary history and geographic origins. **a**, Multi-dimensional scaling plot of *f*_4_-based genetic affinities. The age of the samples is indicated along the vertical axis. CA, Central Asia. **b**, Horse evolutionary history inferred by OrientAGraph^19^ with three migration edges and nine lineages representing key genomic ancestries (coloured as in Fig 1a). The model explains 99.99% of the total variance. The triangular pairwise matrix provides model residuals. The external branch leading to donkey was set to zero to improve visualization. **c**, LOCATOR^20^ predictions of the geographic region where the ancestors of DOM2, tarpan and modern Przewalski’s horses lived. The tarpan and modern Przewalski’s horses do not descend from the same ancestral population as modern domestic horses. The map was drawn using the maps R package^47^.

**Extended Data Fig. 4:**
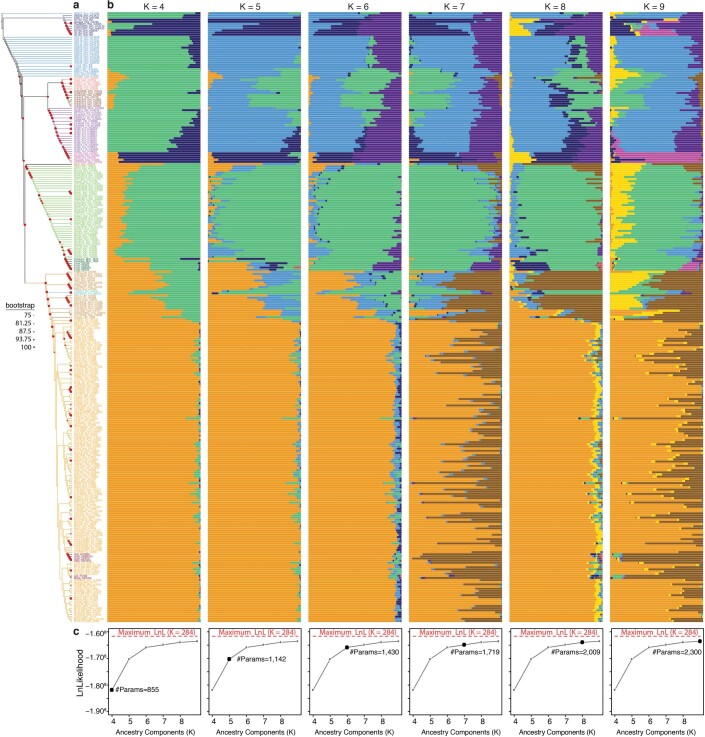
Individual ancestry profiles. **a**, NJ-tree shown in Fig 1b with sample labels as defined in Supplementary Table 1. **b**, Struct-f4 individual ancestry profiles. **c**, Model likelihood. A total of K=4 to K=9 ancestral populations are assumed. LnL = natural log-likelihood.

Horse ancestry profiles in Neolithic Anatolia and Eneolithic Central Asia, including at Botai, maximized a genetic component (coloured green in Fig. 1e, f) that was also substantial in Central and Eastern Europe during the Late Pleistocene (RONPC06_Rom_m34801) and the fourth or third millennium bc (Figs. 1e, 3a, Extended Data Fig. 4). It was, however, absent or moderately present in the Romanian lower Danube (ENEO-ROM), the Dnieper steppes (Ukr11_Ukr_m4185) and the western lower Volga-Don (C-PONT) populations during the sixth to third millennia bc. This indicates possible expansions of Anatolian horses into both Central and Eastern Europe and Central Asia regions, but not into the Western Eurasia steppes. The absence of typical NEO-ANA ancestry rules out expansion from Anatolia into Central Asia across the Caucasus mountains but supports connectivity south of the Caspian Sea prior to about 3500 bc.

## The origins of DOM2 horses

The C-PONT group not only possessed moderate NEO-ANA ancestry, but also was the first region where the typical DOM2 ancestry component (coloured orange in Fig. 1e, f) became dominant during the sixth millennium bc. Multi-dimensional scaling further identified three horses from the western lower Volga-Don region as genetically closest to DOM2, associated with Steppe Maykop (Aygurskii), Yamnaya (Repin) and Poltavka (Sosnovka) contexts, dated to about 3500 to 2600 bc (Figs. 2a, b, 3a). Additionally, genetic continuity with DOM2 was rejected for all horses predating about 2200 bc, especially those from the NEO-ANA group (Supplementary Table 2), except for two late Yamnaya specimens from approximately 2900 to 2600 bc (Turganik (TURG)), located further east than the western lower Volga-Don region (Figs. 2a, b, 3a). These may therefore have provided some of the direct ancestors of DOM2 horses.

Modelling of the DOM2 population with qpADM^17^, rotating^18^ all combinations of 2, 3 or 4 population donors, eliminated the possibility of a contribution from the NEO-ANA population, but indicated possible formation within the WE population, including a genetic contribution of approximately 95% from C-PONT and TURG horses (Supplementary Table 3). This was consistent with OrientAGraph^19^ modelling from nine lineages representing key ancestry combinations, which confirmed the absence of NEO-ANA genetic ancestry in DOM2 and confirmed DOM2 as a sister population to the C-PONT horses (Fig. 3b).

Identifying discrete populations and modelling admixture as single unidirectional pulses, however, was highly challenging given the extent of spatial genetic connectivity. Indeed, the typical DOM2 ancestry component was maximized in the C-PONT group, but declined sharply eastwards (TURG and Central Asia) in the third millennium bc as the proportion of NEO-ANA ancestry increased (Fig. 2a). This suggests a cline of genetic connectivity east of the Western Eurasia steppes and Central Asia, ruling out DOM2 ancestors further east than the western lower Volga-Don and Turganik. A similar genetic cline characterized the region located west of C-PONT, where the typical DOM2 ancestry component declined steadily in the Dnieper steppes, Poland, Turkish Thrace and Hungary in the fifth to third millennia bc. This eliminates the possibility of DOM2 ancestors further west than C-PONT and the Dnieper steppes. Furthermore, patterns of spatial autocorrelations in the genetic data^20^ indicated Western Eurasia steppes as the most likely geographic location of DOM2 ancestors (Fig. 3c). Combined, our results demonstrate that DOM2 ancestors lived in the Western Eurasia steppes, especially the lower Volga-Don, but not in Anatolia, during the late fourth and early third millennia bc.

## Expansion of steppe-related pastoralism

**Extended Data Fig. 5:**
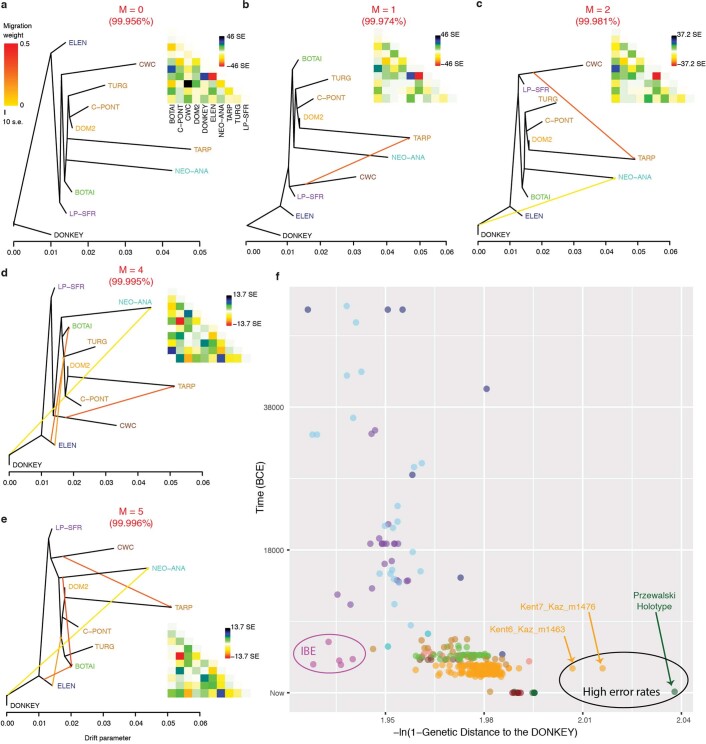
OrientAGraph^19^ population histories and genetic distances to the domestic donkey. **a–e**, OrientAGraph^19^ models and residuals assuming M=0 to M=5 migration edges and considering nine lineages representing key genomic ancestries (colored as in Fig 1a). M=3 is shown in Fig 3b. **f**, Pairwise genetic distances between a given horse and the domestic donkey plotted as a function of the age of the horse specimen considered.

Analyses of ancient human genomes have revealed a massive expansion from the Western Eurasia steppes into Central and Eastern Europe during the third millennium bc, associated with the Yamnaya culture^8,9,11,12,21^. This expansion contributed at least two thirds of steppe-related ancestry to populations of the Corded Ware complex (CWC) around 2900 to 2300 bc^8^. The role of horses in this expansion remained unclear, as oxen could have pulled Yamnaya heavy, solid-wheeled wagons^7,22^. The genetic profile of horses from CWC contexts, however, almost completely lacked the ancestry maximized in DOM2 and Yamnaya horses (TURG and Repin) (Figs. 1e, f, 2a, b) and showed no direct connection with the WE group, including both C-PONT and TURG, in OrientAGraph modelling (Fig. 3b, Extended Data Fig. 5).

The typical DOM2 ancestry was also limited in pre-CWC horses from Denmark, Poland and Czechia, associated with the Funnel Beaker and early Pitted Ware cultures (FB/PWC, FB/POL and ENEO-CZE, respectively). DOM2 ancestry reached a maximum 12.5% in one Hungarian horse dated to the mid-third millennium bc and associated with the Somogyvár-Vinkovci Culture (CAR05_Hun_m2458). qpAdm^17^ modelling indicated that its DOM2 ancestry was acquired following gene flow from southern Thrace (Kan22_Tur_m2386), but not from the Dnieper steppes (Ukr11_Ukr_m4185) (Supplementary Table 3). Combined with the lack of increased horse dispersal during the early third millennium bc (Fig. 2b, Extended Data Fig. 3b), these results suggest that DOM2 horses did not accompany the steppe pastoralist expansion north of the Carpathians.

By around 2200–2000 bc, the typical DOM2 ancestry profile appeared outside the Western Eurasia steppes in Bohemia (Holubice), the lower Danube (Gordinesti II) and central Anatolia (Acemhöyük), spreading across Eurasia shortly afterwards, eventually replacing all pre-existing lineages (Fig 2c, Extended Data Fig. 3c). Eurasia became characterized by high genetic connectivity, supporting massive horse dispersal by the late third millennium and early second millennium bc. This process involved stallions and mares, indicated by autosomal and X-chromosomal variation (Extended Data Fig. 3d), and was sustained by explosive demographics apparent in both mitochondrial and Y-chromosomal variation (Extended Data Fig. 3e, f). Altogether, our genomic data uncover a high turnover of the horse population in which past breeders produced large stocks of DOM2 horses to supply increasing demands for horse-based mobility from around 2200 bc.

**Extended Data Fig. 6:**
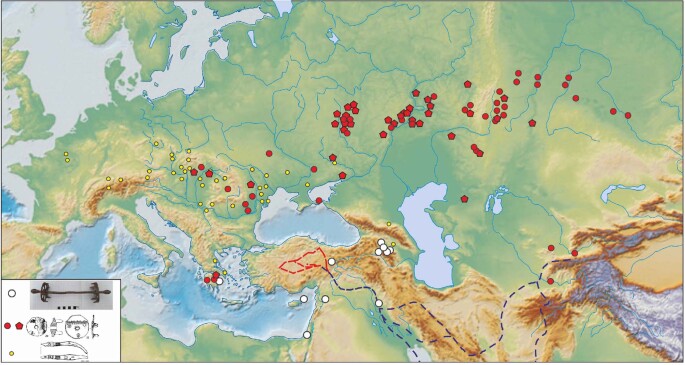
Inter-regional trade and chariot networks, marked by horse cheek pieces, connecting Bronze Age steppe societies, mineral rich Caucasian societies and the Old Assyrian trade network during the period 1,950-1,750 BCE. Documented Near Eastern trade routes are marked with stippled lines (after^23^, supplemented with data from^90,91^ and Pavel F. Kuznetsov).

Of note, the DOM2 genetic profile was ubiquitous among horses buried in Sintashta kurgans together with the earliest spoke-wheeled chariots around 2000–1800 bc^7,9,23,24^ (Extended Data Fig. 6). A typical DOM2 profile was also found in Central Anatolia (AC9016_Tur_m1900), concurrent with two-wheeled vehicle iconography from about 1900 bc^25,26^. However, the rise of such profiles in Holubice, Gordinesti II and Acemhöyük before the earliest evidence for chariots supports horseback riding fuelling the initial dispersal of DOM2 horses outside their core region, in line with Mesopotamian iconography during the late third and early second millennia bc^27^. Therefore, a combination of chariots and equestrianism is likely to have spread the DOM2 diaspora in a range of social contexts from urban states to dispersed decentralized societies^28^.

## DOM2 biological adaptations

**Extended Data Fig. 7:**
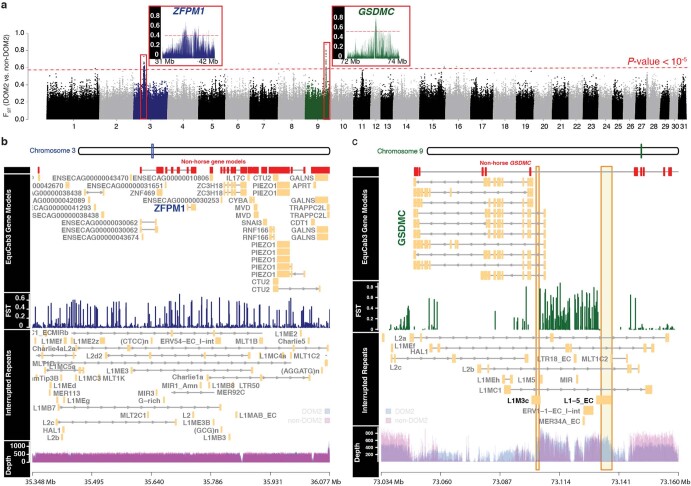
DOM2 selection signatures. **a**, Manhattan plot of F_ST_-differentiation index between DOM2 and non-DOM2 horses along the 31 EquCab3 autosomes. F_ST_ outliers are highlighted using an empirical *P*-value threshold of 10^−5^ (red dashed line). The two outlier regions on chromosomes 3 and 9 are highlighted within red frames. **b**, F_ST_-differentiation index and genomic tracks around the *ZFPM1* gene. Depth represents the accumulated number of reads per position within DOM2 (blue) and non-DOM2 (magenta) genomes. **c**, Same as Panel b at *GSDMC*.

Human-induced DOM2 dispersal conceivably involved selection of phenotypic characteristics linked to horseback riding and chariotry. We therefore screened our data for genetic variants that are over-represented in DOM2 horses from the late third millennium bc (Extended Data Fig. 7). The first outstanding locus peaked immediately upstream of the *GSDMC* gene, where sequence coverage dropped at two L1 transposable elements in all lineages except DOM2. The presence of additional exons in other mammals suggests that independent L1 insertions remodelled the DOM2 gene structure. In humans, *GSDMC* is a strong marker for chronic back pain^29^ and lumbar spinal stenosis, a syndrome causing vertebral disk hardening and painful walking^30^.

The second most differentiated locus extended over approximately 16 Mb on chromosome 3, with the *ZFPM1* gene being closest to the selection peak. *ZFPM1* is essential for the development of dorsal raphe serotonergic neurons involved in mood regulation^31^ and aggressive behaviour^32^. *ZFPM1* inactivation in mice causes anxiety disorders and contextual fear memory^31^. Combined, early selection at *GSDMC* and *ZFPM1* suggests shifting use toward horses that were more docile, more resilient to stress and involved in new locomotor exercise, including endurance running, weight bearing and/or warfare.

## Evolutionary history and origins of tarpan horses

Our analyses elucidate the geographic, temporal and biological origins of DOM2 horses. This study features a diverse ancient horse genome dataset, revealing the presence of deep mitochondrial and/or Y-chromosomal haplotypes in non-DOM2 horses (Supplementary Fig 1). This suggests that yet-unsampled divergent populations contributed to forming several lineages excluding DOM2. This was especially true in the Iberian group (IBE), where the expected genetic distance to the donkey was reduced (Extended Data Fig. 5f), but also in NEO-ANA according to OrientAGraph modelling (Fig 3b). Disentangling exact divergence and ancestry contributions of such unsampled lineages is difficult with the currently available data. It can, however, be stressed that Iberia and Anatolia represent two well-known refugia^33^, where populations could have survived and mixed during Ice Ages.

Finally, our analyses have solved the mysterious origins of the tarpan horse, which became extinct in the early 20th century. The tarpan horse came about following admixture between horses native to Europe (modelled as having 28.8–34.2% and 32.2–33.2% CWC ancestry in OrientAGraph^19^ and qpAdm^17^, respectively) and horses closely related to DOM2. This is consistent with LOCATOR^20^ predicting ancestors in western Ukraine (Fig 3c) and refutes previous hypotheses depicting tarpans as the wild ancestor or a feral version of DOM2, or a hybrid with Przewalski’s horses^34^.

## Discussion

This work resolves longstanding debates about the origins and spread of domestic horses. Whereas horses living in the Western Eurasia steppes in the late fourth and early third millennia bc were the ancestors of DOM2 horses, there is no evidence that they facilitated the expansion of the human genetic steppe ancestry into Europe^8,9^ as previously hypothesized^7^. Instead of horse-mounted warfare, declining populations during the European late Neolithic^35^ may thus have opened up an opportunity for a westward expansion of steppe pastoralists. Yamnaya horses at Repin and Turganik carried more DOM2 genetic affinity than presumably wild horses from hunter-gatherer sites of the sixth millennium bc (NEO-NCAS, from approximately 5500–5200 bc), which may suggest early horse management and herding practices. Regardless, Yamnaya pastoralism did not spread horses far outside their native range, similar to the Botai horse domestication, which remained a localized practice within a sedentary settlement system^2,36^. The globalization stage started later, when DOM2 horses dispersed outside their core region, first reaching Anatolia, the lower Danube, Bohemia and Central Asia by approximately 2200 to 2000 bc, then Western Europe and Mongolia soon afterwards, ultimately replacing all local populations by around 1500 to 1000 bc. This process first involved horseback riding, as spoke-wheeled chariots represent later technological innovations, emerging around 2000 to 1800 bc in the Trans-Ural Sintashta culture^7^. The weaponry, warriors and fortified settlements associated with this culture may have arisen in response to increased aridity and competition for critical grazing lands, intensifying territoriality and hierarchy^37^. This may have provided the basis for the conquests over the subsequent centuries that resulted in an almost complete human and horse genetic turnover in Central Asian steppes^11,21^. The expansion to the Carpathian basin^38^, and possibly Anatolia and the Levant, involved a different scenario in which specialized horse trainers and chariot builders spread with the horse trade and riding. In both cases, horses with reduced back pathologies and enhanced docility would have facilitated Bronze Age elite long-distance trade demands and become a highly valued commodity and status symbol, resulting in rapid diaspora. We, however, acknowledge substantial spatiotemporal variability and evidential bias towards elite activities, so we do not discount additional, harder to evidence, factors in equine dispersal.

**Extended Data Fig. 8:**
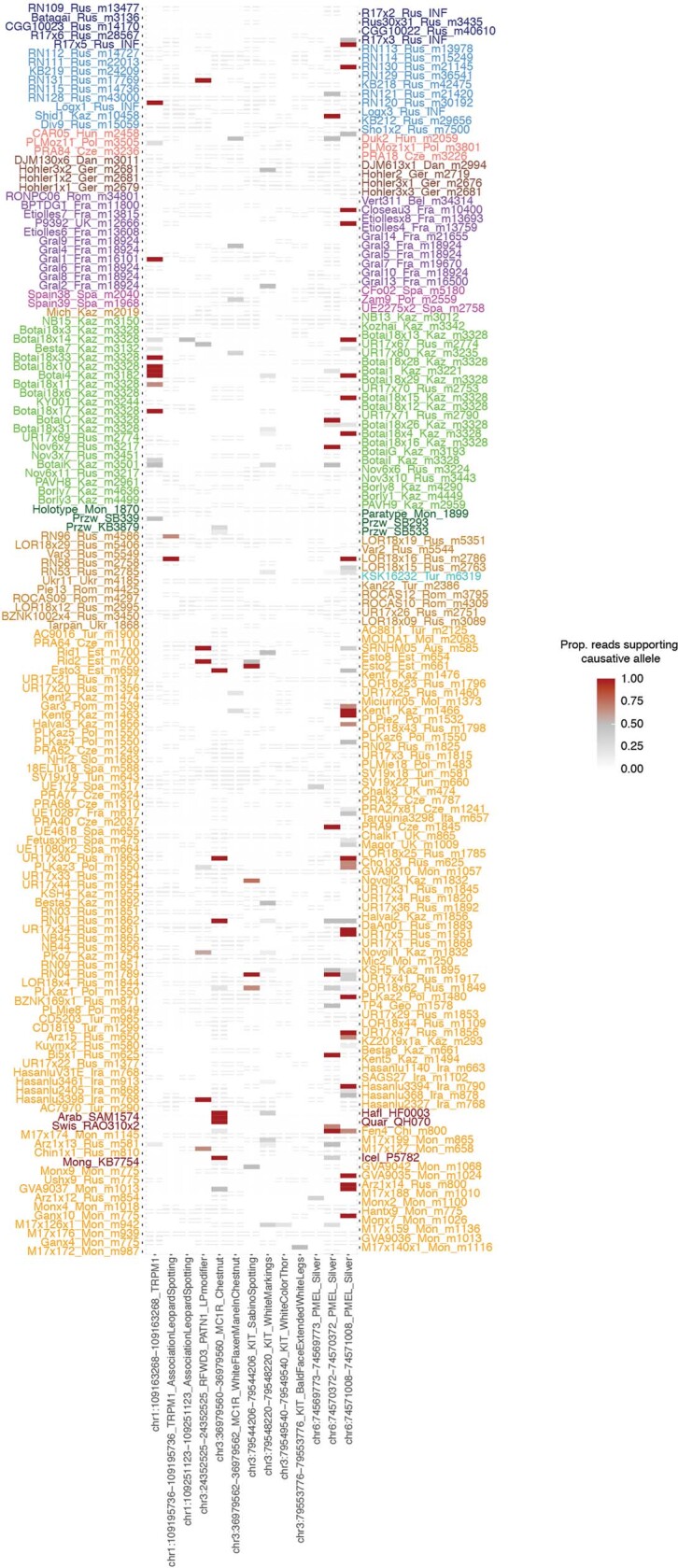
Normalized read coverage supporting the presence of causative alleles for coat coloration variation. Each column represents a particular genome position where genetic polymorphisms associated or causative for coat coloration patterns have been described. The exact EquCab3 genome coordinates are indicated in the locus label. Specimens (rows) are ordered according to their phylogenetic relationships, as shown in Fig 1b. The color gradient is proportional to the fraction of reads carrying the causative variant. Loci that are not covered following trimming and rescaling of individual BAM sequence alignment files are indicated with a white cross.

Our results also have important implications for mechanisms underpinning two major language dispersals. The expansion of the Indo-European language family from the Western Eurasia steppes has traditionally been associated with mounted pastoralism, with the CWC serving as a major stepping stone in Europe^39–41^. However, while there is overwhelming lexical evidence for horse domestication, horse-drawn chariots and derived mythologies in the Indo-Iranian branch of the Indo-European family, the linguistic indications of horse-keeping practices at the deeper Proto-Indo-European level are in fact ambiguous^42^ (Supplementary Discussion) . The limited presence of horses in CWC assemblages^43^ and the local genetic makeup of CWC specimens reject scenarios in which horses were the primary driving force behind the initial spread of Indo-European languages in Europe^44^. By contrast, DOM2 dispersal in Asia during the early-to-mid second millennium bc was concurrent with the spread of chariotry and Indo-Iranian languages, whose earliest speakers are linked to populations that directly preceded the Sintashta culture^11,12,45^. We thus conclude that the new package of chariotry and improved breed of horses, including chestnut coat colouration documented both linguistically (Supplementary Discussion) and genetically (Extended Data Fig. 8), transformed Eurasian Bronze Age societies globally within a few centuries after about 2000 bc. The adoption of this new institution, whether for warfare, prestige or both, probably varied between decentralized chiefdoms in Europe and urbanized states in Western Asia. The results thus open up new research avenues into the historical developments of these different societal trajectories.

## Methods

### Radiocarbon dating

A total of 170 new radiocarbon dates were obtained in this study. Dating was carried out at the Keck Carbon Cycle AMS Laboratory, UC Irvine following collagen extraction and ultra-filtration from approximately 1 g of osseous material. IntCal20 calibration^48^ was performed using OxCalOnline^49^.

### Genome sequencing

All samples were collected with permission from the organizations holding the collections and documented through official authorization letters for partially destructive sampling from local authorities. Samples were processed for DNA extraction, library construction and shallow sequencing in the ancient DNA facilities of the Centre for Anthropobiology and Genomics of Toulouse (CAGT), France. The overall methodology followed the work from Seguin–Orlando and colleagues^50^. It involved: (1) powdering a total of 100–590 mg of osseous material using the Mixel Mill MM200 (Retsch) Micro-dismembrator; (2) extracting DNA following the procedure Y2 from Gamba and colleagues^51^, tailored to facilitate the recovery of even the shortest DNA fragments; (3) treating DNA extracts with the USER (NEB) enzymatic cocktail to eliminate a fraction of post mortem DNA damage^13^; (4) constructing from double-stranded DNA templates DNA libraries in which two internal indexes are added during adapter ligation and one external index is added during PCR amplification; and (5) amplification, purification and quantification of DNA libraries before pooling 20–50 DNA libraries for low-depth sequencing on the Illumina MiniSeq instrument (paired-end mode, 2 × 80). All three indexes of each library were unique in a given sequencing pool.

Raw fastQ files were demultiplexed, trimmed and collapsed when individual read pairs showed significant overlap using AdapterRemoval2^52^ (version 2.3.0), disregarding reads shorter than 25 bp. Processed reads were then aligned against the nuclear and mitochondrial horse reference genomes^53,54^, and appended with the Y-chromosome contigs from^55^ using the Paleomix bam_pipeline (version 1.2.13.2) with the mapping parameters recommended by Poullet and Orlando^56^. Sequencing reads representing PCR duplicates or showing a mapping quality below 25 were disregarded. DNA fragmentation and nucleotide misincorporation patterns were assessed on the basis of 100,000 random mapped reads using mapDamage2^57^ (version 2.0.8). Paleomix returned provisional estimates of endogenous DNA content and clonality, as defined by the fraction of retained reads mapping uniquely against the horse reference genomes and those mapping at the same genomic coordinates, respectively. These numbers guided further experimental decisions, including (1) the sequencing effort to be performed per individual library; (2) the preparation of additional libraries from left-over aliquots of USER-treated DNA extracts, or following treatment of DNA extract aliquots with the USER enzymatic cocktail; and (3) the preparation of additional DNA extracts. After initial screening for library content, sequencing was carried out on the Illumina HiSeq4000 instruments from Genoscope (paired-end mode, 2 × 76; France Génomique), except for four samples (BPTDG1_Fra_m11800, Closeau3_Fra_m10400, Novoil1_Kaz_m1832 and Novoil2_Kaz_m1832), for which sequencing was done at Novogene Europe on an Illumina NovaSeq 6000 instrument (S4 lanes, paired-end mode, 2 × 150). Overall, we obtained sequence data for a total of 264 novel ancient horse specimens and 1,029 DNA libraries (980 new), summing up to 31.86 billion sequencing read pairs and 100.82 billion collapsed read pairs, which was sufficient to characterize 226 novel ancient genomes showing a genomic depth-of-coverage of at least 1× (median 2.80-fold, maximum 25.76-fold) (Supplementary Table 1).

### Allele sampling, sequencing error rates, genome rescaling and trimming

Following previous work^5,58^, error rates are defined as the excess of mutations that are private to the ancient genome, relative to a modern genome considered as error-free. Mutations were polarized using an outgroup genome representing a consensus built from seven male specimens of diverse equine species (*Equus africanus somaliensis*, *Equus asinus*, *Equus burchelli*, *Equus grevyi*, *Equus hartmannae*, *Equus hemionus onager* and *Equus kiang*^59^), according to a majority rule in which at maximum 2 of the 7 individuals showed an alternative allele. Minor and major alleles were identified using ANGSD^60^ (version 0.933-86-g3fefdc4, htslib: 1.10.2-106-g9c35744) and the following parameters: -baq 0 -doMajorMinor 2 -uniqueOnly 1 -minMapQ 25 -minQ 30 -minind 7 -doCounts 1 -doMaf 1.

Error rate estimates ranged between 0.000337 and 0.003966 errors per site and revealed that nucleotide C→T and G→A misincorporation rates were still inflated relative to their reciprocal substitution types (T→C and A→G), despite USER treatment. Therefore, individual BAM alignment files were processed to further reduce nucleotide misincorporation rates. To achieve this, we used PMDtools^61^ (version 0.60) to bin apart reads likely containing post mortem DNA damage (--threshold 1; DAM) from those that did not (--upperthreshold 1; NODAM). NODAM-aligned reads were then directly trimmed by 5 bp at their ends, where individual base qualities generally drop. The base quality of aligned DAM reads was first rescaled using mapDamage2^57^ (version 2.0.8), penalizing all instances of potential derivatives of post mortem cytosine deamination, then further trimmed by 10 bp at both ends. The resulting NODAM and DAM aligned reads were merged again to obtain final BAM sequence alignments. Final error rate estimates ranged between 0.000080 and 0.000933 errors per site (Supplementary Table 1).

### Uniparentally inherited markers and coat colouration

Mitochondrial genomes for the 264 newly sequenced samples were characterized from quality-filtered BAM alignment files (minMapQ=25, minQ=30), using a majority rule requiring at least five individual reads per position. Their resulting complete mitochondrial genome sequences were aligned together with a total of 193 sequences previously characterized^3,5,14,15,58,62,63^ using mafft^64^ (version 7.407). Sequence alignments were split into six partitions, following previous work^5^, including the control region, all tRNAs, both rRNAs and each codon position considered separately. Maximum-likelihood phylogenetic reconstruction was performed using RAxML^65^ (version 8.2.11) with default parameters, and assessing node support from a total of 100 bootstrap pseudo-replicates. The same partitions were provided as input for BEAST^66^ (version 2.5.1), together with calibrated radiocarbon years (Supplementary Table 1). Specimens lacking direct radiocarbon dates or identified as not belonging to the DOM2 cluster were disregarded (Supplementary Table 1). While the former ensured precise tip-calibration for molecular clock estimation (assuming uncorrelated log-normal relaxed model), the latter prevented misinterpreting spatial variation in the population structure as changes in the effective population size^67^. The best substitution model was selected from ModelGenerator^68^ (version 0.85) and Bayesian Skyline plots^69^ were retrieved following 1,000,000,000 generations, sampling 1 every 1,000 and disregarding the first 30% as burn-in. Convergence was visually checked in Tracer^70^ (version 1.7.2).

The Y-chromosome maximum-likelihood tree was constructed calling individual haplotypes from trimmed and rescaled BAM sequence alignments against the contigs described by Felkel and colleagues^55^, filtered for single copy MSY regions. The final multifasta sequence alignment included sites covered in at least 20% of the specimens, pseudo-haploidizing each position and filtering out transitions, as done with autosomal data. It was further restricted to specimens showing at least 20% of the final set of positions covered. This represented a total of 3,195 nucleotide transversions for 142 specimens. The final tree was computed using IQtree (version 1.6.12), following AICc selection of the best substitution model and 1,000 ultrafast bootstrap approximation for assessing node support^71,72^. The Y-chromosome Bayesian skyline plot was obtained following the same procedure as above. Maximum-likelihood trees and Bayesian skyline plots are shown in Supplementary Fig 1 and Extended Data Fig. 3e, f, respectively.

The presence of alleles associated with or causative for a diversity of coat colouration changes was investigated using individual BAM read alignments. For a total of 43 genomic locations representing biallelic SNPs, we simply counted the proportion of reads supporting the associated or causative allele. Results were summarized in the heat map shown in Extended Data Fig. 8, with respect to the sample ordering displayed in the neighbour-joining phylogenetic reconstruction, and limited to those 13 loci that were polymorphic in our horse panel for clarity.

### Neighbour-joining phylogeny, genetic continuity and population modelling

Phylogenetic affinities were first estimated by performing a BioNJ tree reconstruction with FastME^73^ (version 2.1.4), based on the pairwise matrix of genetic distances inferred from the bed2diffs_v1 program^16^. Node supports were assessed using a total of 100 bootstrap pseudo-replicates. The ‘goodness-of-fit’ of the neighbour-joining tree to the data was evaluated by comparing the patristic distances and raw pairwise distances. Patristic distances were obtained from the ape^74^ R package (version 5.5) and their ratios to raw pairwise distances were averaged for each given individual (Fig 1c). Averaged ratios equal to one support perfect phylogenetic placement for the specimen considered.

Genetic continuity between each individual specimen predating about 2200 bc and DOM2 horses was tested following the methodology from Schraiber^75^, which implements a likelihood-ratio test to compare the statistical support for placing DOM2 and the ancient specimen in a direct line of ancestry or as two sister groups. This methodology relies on exact allele frequency estimates within DOM2 and read counts for putatively ancestral ancient samples. To exclude residual sequencing errors within DOM2 horses, we, thus, conditioned these analyses on variants segregating at least as doubletons in positions covered in at least 75% of the DOM2 samples. Linked variation was pruned using Plink^76^ (version v.1.9), with the following parameters, --indep-pairwise 50 10 0.2, which provided a panel of about 1.4 million transversions. Allele frequencies were polarized considering the outgroup genome used for measuring error rates. Results from direct ancestry tests are summarized in Supplementary Table 2.

The complex genetic makeup of some individuals (CAR05_Hun_m2458 and Kan22_Tur_m2386) and/or group of individuals (DOM2) was investigated using the *f*_*4*_-statistics-based ancestry decomposition approach implemented in qpAdm^17^ (version 7.0), in which one particular (group of) individual(s) is modelled as a linear, additive combination of candidate population sources (‘left’ populations). We followed the rotating strategy recommended by Harney and colleagues^18^ to assess all possible combinations of two, three and four donors (‘left’) selected from a total of 18 populations. The remaining 14, 15 and 16 populations were used as reference (‘right’) populations (Supplementary Table 3).

We selected a total of nine horse lineages representing the main phylogenetic clusters, and carrying genetic ancestry profiles representative of the complete dataset, to model the population evolutionary history using OrientAGraph^19^ (version 1.0). By implementing a network orientation subroutine that enables throughout exploration of the graph space, OrientAGraph constitutes a marked advancement in the automated inference of admixture graphs. We considered scenarios from zero to five migration pulses (*M* = 0 to 5; Extended Data Fig. 5a–e), and the population model assuming *M* = 3 is represented in Fig 3b. This analysis was conditioned on sites covered at least in one specimen of each population group. This filter yielded a set of 7,936,493 fully orthologous nucleotide transversions.

### Struct-f4, ancestry components and multi-dimensional scaling

We extended the Struct-f4 package so as to assess individual genetic affinities within a panel of genomes, and to decompose them into *K* genetic ancestries. Struct-f4, thus, achieves similar objectives to other clustering methods, such as ADMIXTURE^77^ and Ohana^78^, but does not assume Hardy–Weinberg equilibrium. The latter assumption is known to cause misinterpretation of highly drifted samples as ancestral homogeneous groups instead of highly derived mixtures from multiple populations, as thoroughly described elsewhere^79^. To circumvent this, Struct-f4 relies on the calculation of the widely used *f*_*4*_ statistics, which were originally devised not only to test for admixture, but also to quantify the drift between the internal nodes of a population tree. The latter provides a direct representation of the true ancestral populations. Overall, Struct-f4 thus implements a more natural and robust (model-free) approach than other clustering alternatives.

Struct-f4 is based on a mixture model that parametrizes the drift that occurred between a given number of *K* pre-defined ancestral populations, and the mixing coefficient of each individual. Model parameters are estimated using an adaptive Metropolis–Hastings Markov chain Monte Carlo integration, identifying optimal numerical solutions for parameters by means of likelihood maximization. Struct-f4 was validated following extensive coalescent simulations with fastsimcoal2^80^ (version 2.6.0.3). An example of such simulation designed to mimic the complex horse evolutionary history is provided in Extended Data Fig. 2, based on mutation and recombination rates of 2.3 × 10^−8^ and 10^−8^ events per generation and bp, respectively. Struct-f4 is implemented in Rcpp and only takes the full set of *f*_*4*_-statistics as input to automatically return individual ancestry coefficients, without requiring pre-defined, ad-hoc sets of reference and test populations.

Multi-dimensional scaling was carried out based on the co-ancestry semi-matrix summarizing the drift measured between each pair of individuals, as returned by Struct-f4, removing the domestic donkey outgroup prior to using the cmdscale R function.

### Isolation by distance and spatial connectivity

Spatial barriers to gene flow prior to about 3000 bc, between about 3000 and 2000 bc and following about 2000 bc were run using EEMS^16^ (built with Eigen version 3.2.2 and Boost version 1.57, and using rEEMSplots version 0.0.0.9000) for 50 million iterations and considering a burn-in of 15 million iterations. Convergence was ensured from visual inspection of likelihood trajectories as well as by the strong correlation obtained between the observed and fitted genetic dissimilarities. Pie-charts depicting the ancestry proportions inferred by Struct-f4 were overlaid on the migration surfaces to facilitate tracking the geographic position of each excavation site, averaging ancestry proportions or using individual ancestry profiles if only one sample was characterized genetically at that location. Spatial pie-chart projection was carried out using the draw.pie R function from the mapplots package^81^ (version 1.5.1). The size of each individual pie-chart was commensurate with the number of samples excavated at a given geographic location, provided that the number of samples was lower than 10, while set to a constant maximum radius otherwise.

Partial Mantel tests measuring the correlation between geographic and genomic distances over time were carried out using the ncf R package^82^ (version 1.2.9). This test corrected for the time variation present within each window, similar to the approach described by Loog and colleagues^83^. Haversine geographic distances between pairs of ancient samples were computed using the geosphere package (version 1.5.10) in R^84^, from the corresponding longitude and latitude coordinates, while radiocarbon date ages were considered as point estimates (Supplementary Table 1). The matrix of pairwise genetic distances was obtained from the bed2diffs_v1 program provided together with the EEMS software^16^. The analysis was carried out for autosomes and the X chromosome separately, so as to investigate possible sex-bias in horse dispersal. Confidence intervals were calculated by sampling with replacement individuals within each time window.

Sliding time windows (step size = 250 years) were broadened forward in time until including at least ten specimens covering two-thirds of the total geographic area sampled in this study. The area delimited by a set or subset of GPS coordinates was calculated using the GeoRange R package^85^ (version 0.1.0) and the age of the window was set to the average age amongst the samples included. Additionally, pairwise distances involving samples located less than 500 km away and separated by less than 500 years were masked in the corresponding matrices to estimate the patterns of isolation by distance between demes, instead of within demes. This whole scheme was designed to prevent regional effects, caused by the over-representation of particular regions in specific time intervals.

The LOCATOR^20^ program (version 1.2) was run using a geolocated reference panel consisting of all non-DOM2 horses (*n* = 136), except the tarpan and the four Przewalski’s horses present in our dataset, and considering nucleotide transversions covered at least in 75% of the samples, for a total of 3,194,008 SNPs. The geographic origin of each DOM2 horse was then estimated from the geographic structure defined by the populations present in the reference panel. Default parameters were used, except that the width of each neural layer was 512 (instead of 256). The best run was selected as the one showing the lowest validation error from a total of 50 independent runs. The analysis was repeated for the tarpan as well as the four Przewalski’s horses present in our dataset.

### Selection scans

To pinpoint genetic changes potentially underlying biological adaptation within DOM2 horses, we contrasted the frequency of each nucleotide transversion in our dataset (*n* = 10,205,277) in DOM2 (*n* = 141) and non-DOM2 horses (*n* = 142). The extensive number of samples represented provided unprecedented resolution into patterns of allele frequency differentiation, and encompassed the largest diversity of non-DOM2 horses characterized to date. Weir and Cockerham *F*_ST_ index values between both groups were calculated using Plink^76^ (version 1.9) and visualized using the GViz R package^86^ (version 1.36.2), together with external genomic tracks provided by the gene models annotated for EquCab3 (Ensembl v0.102) and the interrupted repeats precomputed for the same assembly and stored in the UCSC browser.

### Reporting summary

Further information on research design is available in the Nature Research Reporting Summary linked to this paper.

## Online content

Any methods, additional references, Nature Research reporting summaries, source data, extended data, supplementary information, acknowledgements, peer review information; details of author contributions and competing interests; and statements of data and code availability are available at 10.1038/s41586-021-04018-9.

## Supplementary information

Supplementary Information **Supplementary Methods; Supplementary Discussion; Supplementary Notes**. This file provides full description of archaeological material and contexts, develops the methodology underlying genome analyses, and summarizes linguistic information on Indo-European equine and Indo-Iranian chariotry terminology. A full list of supplementary references is provided.

Reporting Summary

Peer Review File

Supplementary Fig. 1 **Mitochondrial and Y-chromosome phylogenies** This figure provides ML phylogenies mtDNA (a) and the Y-chromosome (b), with full sample labels. Node support is assessed using 100 bootstrap pseudo-replicates.

Supplementary Tables 1–3 Table1 provides details on archeological contexts and DNA data. Table 2 presents the results of genetic continuity tests, while Table 3 summarizes the best ancestry profiles identified with qpAdm.

## Data Availability

All collapsed and paired-end sequence data for samples sequenced in this study are available in compressed fastq format through the European Nucleotide Archive under accession number PRJEB44430, together with rescaled and trimmed bam sequence alignments against both the nuclear and mitochondrial horse reference genomes. Previously published ancient data used in this study are available under accession numbers PRJEB7537, PRJEB10098, PRJEB10854, PRJEB22390 and PRJEB31613, and detailed in Supplementary Table 1. The genomes of ten modern horses, publicly available, were also accessed as indicated in their corresponding original publications^59,63,87–89^.

## Code availability

The Struct-f4 software is available without restriction on Bitbucket (https://bitbucket.org/plibradosanz/structf4/src/master/).

## References

[1] Kelekna, P. *The Horse in Human History* (Cambridge Univ. Press, 2009).

[2] Outram AK (2009). The earliest horse harnessing and milking. Science.

[3] Gaunitz C (2018). Ancient genomes revisit the ancestry of domestic and Przewalski’s horses. Science.

[4] Olsen, S. L. in *Horses and Humans: The Evolution of Human Equine Relationships* (eds Olsen S. L.et al.) 81–113 (Archaeopress, 2006).

[5] Fages A (2019). Tracking five millennia of horse management with extensive ancient genome time series. Cell.

[6] Guimaraes, S. et al. Ancient DNA shows domestic horses were introduced in the southern Caucasus and Anatolia during the Bronze Age. *Sci. Adv*. **6**, eabb0030 (2020). 10.1126/sciadv.abb0030 PMC7494339 32938680

[7] Anthony, D. W. *The Horse, the Wheel and Language* (Princeton Univ. Press, 2007).

[8] Haak W (2015). Massive migration from the steppe was a source for Indo-European languages in Europe. Nature.

[9] Allentoft ME (2015). Population genomics of Bronze Age Eurasia. Nature.

[10] Demoule, J. P. Mais où sont passés les Indo-Européens ? Le mythe d'origine de l'Occident (Le Seuil, 2014).

[11] de Barros Damgaard P (2018). 137 ancient human genomes from across the Eurasian steppes. Nature.

[12] Narasimhan VM (2019). The formation of human populations in South and Central Asia. Science.

[13] Rohland N, Harney E, Mallick S, Nordenfelt S, Reich D (2015). Partial uracil-DNA-glycosylase treatment for screening of ancient DNA. Philos. Trans. R. Soc. Lond. B.

[14] Schubert M (2014). Prehistoric genomes reveal the genetic foundation and cost of horse domestication. Proc. Natl Acad. Sci. USA.

[15] Librado P (2015). Tracking the origins of Yakutian horses and the genetic basis for their fast adaptation to subarctic environments. Proc. Natl Acad. Sci. USA.

[16] Petkova D, Novembre J, Stephens M (2016). Visualizing spatial population structure with estimated effective migration surfaces. Nat. Genet..

[17] Patterson N (2012). Ancient admixture in human history. Genetics.

[18] Harney É, Patterson N, Reich D, Wakeley J (2021). Assessing the performance of qpAdm: a statistical tool for studying population admixture. Genetics.

[19] Molloy, E. K., Durvasula, A. & Sankararaman, S. Advancing admixture graph estimation via maximum likelihood network orientation. *Bioinformatics* **37**, i142–i150 (2021). 10.1093/bioinformatics/btab267 PMC8336447 34252951

[20] Battey C, Ralph PL, Kern AD (2020). Predicting geographic location from genetic variation with deep neural networks. eLife.

[21] de Barros Damgaard P (2018). The first horse herders and the impact of early Bronze Age steppe expansions into Asia. Science.

[22] Reinhold, S. et al. in *Appropriating Innovations: Entangled Knowledge in Eurasia, 5000–1500 bce* (eds Stockhammer, P. W. & Maran, J.) 78–97 (Oxbow Books, 2017).

[23] Kristiansen, K. in *Trade and Civilization. Economic Networks and Cultural Ties, from Prehistory to the Early Modern Period* (eds Kristiansen, K. et al.) (Cambridge Univ. Press, 2018).

[24] Chechushkov I. V., & Epimakhov, A. V. in *The Puzzle of Indo-European Origins and Dispersals: Archeology, Linguistics and Genetics* (eds Kristiansen, K. et al.) (Cambridge Univ. Press, in the press).

[25] Littauer MA, Crouwel JH (1996). The origin of the true chariot. Antiquity.

[26] Lindner S (2020). Chariots in the Eurasian Steppe: a Bayesian approach to the emergence of horse-drawn transport in the early second millennium BC. Antiquity.

[27] Moorey PRS (1970). Pictorial evidence for the history of horse-riding in Iraq before the Kassite period. . Iraq.

[28] Kanne, K. Riding, ruling, and resistance equestrianism and political authority in the Hungarian Bronze Age. *Curr. Anthropol*. (in the press).

[29] Suri P (2018). Genome-wide meta-analysis of 158,000 individuals of European ancestry identifies three loci associated with chronic back pain. PLoS Genet..

[30] Jiang H (2020). Two GWAS-identified variants are associated with lumbar spinal stenosis and Gasdermin-C expression in Chinese population. Sci. Rep..

[31] Tikker L (2020). Inactivation of the GATA cofactor ZFPM1 results in abnormal development of dorsal raphe serotonergic neuron subtypes and increased anxiety-like behavior. J. Neurosci..

[32] Takahashi A, Miczek KA (2014). Neurogenetics of aggressive behavior: studies in rodents. Curr. Top. Behav. Neurosci..

[33] Schmitt T, Varga Z (2012). Extra-Mediterranean refugia: the rule and not the exception?. Frontiers Zool..

[34] Spasskaya, N. N., & Pavlinov, I. in *Zoological Research* (Arch. Zoological Museum, Moscow State Univ., 2016).

[35] Colledge S, Conolly J, Crema E, Shennan S (2019). Neolithic population crash in northwest Europe associated with agricultural crisis. Quat. Res..

[36] Outram, A. K. & Bogaard, A. *Subsistence and Society in Prehistory: New Directions in Economic Archaeology* (Cambridge Univ. Press, 2019).

[37] Anthony, D. W. in Social Complexity in Prehistoric Eurasia: Monuments, Metals and Mobility (eds Hanks, B. K. & Lindruff, K. M.) Ch. 4 (2009).

[38] Maran, J., Bajenaru, R., Ailincai, S.-C., Popescu, A.-D. & Hansen, S. I. Objects, ideas and travelers. Contacts between the Balkans, the Aegean and Western Anatolia during the Bronze and Early Iron Age. In: *Proc. of the Conference in Tulcea 10-13 November, 2017* (Rudolf Habelt, 2020).

[39] Glob, P. V. *Denmark: An Archaeological History from the Stone Age to the Vikings* (Cornell Univ. Press, 1971).

[40] Gimbutas M (1977). The first wave of Eurasian Steppe pastoralists into Copper Age Europe. J. Indo. Eur. Stud..

[41] Anthony DW (1986). The “Kurgan Culture,” Indo-European origins, and the domestication of the horse: a reconsideration. Curr. Anthropol..

[42] Renfrew C (1989). They ride horses, don’t they?: Mallory on the Indo-Europeans. Antiquity.

[43] Vandkilde, H. *Culture and Change in Central European Prehistory* (Aarhus Univ. Press, 2007).

[44] Häusler, A. in *Indogermanen und das Pferd* (eds Hänsel, B. & Zimmer, S.) 217–257 (Archaeolingua Alapitvany, 1994).

[45] Kroonen, G., Barjamovic, G. & Peyrot, M.Linguistic supplement to de Barros Damgaard et al. 2018: Early Indo-European languages, Anatolian, Tocharian and Indo-Iranian https://zenodo.org/record/1240524#.YFtLgGjTVMQ (2018).

[46] South A (2011). rworldmap: a new R package for mapping global data. R J..

[47] Brownrigg, R. maps: draw geographical maps. R package version 3.3.0 https://CRAN.R-project.org/package=maps (2018).

[48] Reimer P (2020). The IntCal20 Northern Hemisphere radiocarbon age calibration curve (0–55 cal kBP). Radiocarbon.

[49] Ramsey CB (2009). Bayesian analysis of radiocarbon dates. Radiocarbon.

[50] Seguin-Orlando A (2021). Heterogeneous hunter-gatherer and steppe-related ancestries in Late Neolithic and Bell Beaker genomes from present-day France. Curr. Biol..

[51] Gamba C (2016). Comparing the performance of three ancient DNA extraction methods for high-throughput sequencing. Mol. Ecol. Resour..

[52] Schubert M, Lindgreen S, Orlando L (2016). AdapterRemoval v2: rapid adapter trimming, identification, and read merging. BMC Res. Notes.

[53] Kalbfleisch TS (2018). Improved reference genome for the domestic horse increases assembly contiguity and composition. Commun. Biol..

[54] Xu X, Arnason U (1994). The complete mitochondrial DNA sequence of the horse, *Equus caballus*: extensive heteroplasmy of the control region. Gene.

[55] Felkel S (2019). The horse Y chromosome as an informative marker for tracing sire lines. Sci. Rep..

[56] Poullet M, Orlando L (2020). Assessing DNA sequence alignment methods for characterizing ancient genomes and methylomes. Front. Ecol. Evol..

[57] Jónsson H, Ginolhac A, Schubert M, Johnson PLF, Orlando L (2013). mapDamage2.0: fast approximate Bayesian estimates of ancient DNA damage parameters. Bioinformatics.

[58] Orlando L (2013). Recalibrating *Equus* evolution using the genome sequence of an early Middle Pleistocene horse. Nature.

[59] Jónsson H (2014). Speciation with gene flow in equids despite extensive chromosomal plasticity. Proc. Natl Acad. Sci. USA.

[60] Korneliussen TS, Albrechtsen A, Nielsen R (2014). ANGSD: analysis of next generation sequencing data. BMC Bioinformatics.

[61] Skoglund P (2014). Separating endogenous ancient DNA from modern day contamination in a Siberian Neandertal. Proc. Natl Acad. Sci. USA.

[62] Librado P (2017). Ancient genomic changes associated with domestication of the horse. Science.

[63] Der Sarkissian C (2015). Evolutionary genomics and conservation of the endangered Przewalski’s horse. Curr. Biol..

[64] Katoh K, Standley DM (2013). MAFFT multiple sequence alignment software version 7: improvements in performance and usability. Mol. Biol. Evol..

[65] Stamatakis A (2014). RAxML version 8: a tool for phylogenetic analysis and post-analysis of large phylogenies. Bioinformatics.

[66] Bouckaert R (2019). BEAST 2.5: An advanced software platform for Bayesian evolutionary analysis. PLoS Comput. Biol..

[67] Heller R, Chikhi L, Siegismund HR (2013). The confounding effect of population structure on Bayesian skyline plot inferences of demographic history. PLoS ONE.

[68] Keane TM, Creevey CJ, Pentony MM, Naughton TJ, Mclnerney JO (2006). Assessment of methods for amino acid matrix selection and their use on empirical data shows that ad hoc assumptions for choice of matrix are not justified. BMC Evol. Biol..

[69] Drummond AJ, Rambaut A, Shapiro B, Pybus OG (2005). Bayesian coalescent inference of past population dynamics from molecular sequences. Mol. Biol. Evol..

[70] Rambaut A, Drummond AJ, Xie D, Baele G, Suchard MA (2018). Posterior summarization in Bayesian phylogenetics using Tracer 1.7. Syst Biol.

[71] Nguyen L-T, Schmidt HA, von Haeseler A, Minh BQ (2015). IQ-TREE: a fast and effective stochastic algorithm for estimating maximum-likelihood phylogenies. Mol. Biol. Evol..

[72] Hoang DT, Chernomor O, von Haeseler A, Minh BQ, Vinh LS (2018). UFBoot2: improving the ultrafast bootstrap approximation. Mol. Biol. Evol..

[73] Lefort V, Desper R, Gascuel O (2015). FastME 2.0: a comprehensive, accurate, and fast distance-based phylogeny inference program. Mol. Biol. Evol..

[74] Paradis E, Schliep K (2019). ape 5.0: an environment for modern phylogenetics and evolutionary analyses in R. Bioinformatics.

[75] Schraiber J (2018). Assessing the relationship of ancient and modern populations. Genetics.

[76] Purcell S (2007). PLINK: a tool set for whole-genome association and population-based linkage analyses. Am. J. Hum. Genet..

[77] Alexander DH, Novembre J, Lange K (2009). Fast model-based estimation of ancestry in unrelated individuals. Genome Res..

[78] Cheng JY, Mailund T, Nielsen R (2017). Fast admixture analysis and population tree estimation for SNP and NGS data. Bioinformatics.

[79] Lawson DJ, van Dorp L, Falush D (2018). A tutorial on how not to over-interpret STRUCTURE and ADMIXTURE bar plots. Nat. Commun..

[80] Excoffier L, Dupanloup I, Huerta-Sánchez E, Sousa VC, Foll M (2013). Robust demographic inference from genomic and SNP data. PLoS Genet..

[81] Gerritsen, H. mapplots: data visualisation on maps. R package version 1.5.1 https://CRAN.R-project.org/package=mapplots (2018).

[82] Bjornstad, O. N. & Cai, J. ncf: spatial covariance functions. R package version 1.2-9 http://ento.psu.edu/directory/onb1 (2020).

[83] Loog L (2017). Estimating mobility using sparse data: application to human genetic variation. Proc. Natl Acad. Sci. USA.

[84] Hijmans, R. J., Williams, E. & Vennes, C. E.. geosphere: spherical trigonometry. R package version 1.5.1 (2019).

[85] Boyle, J. GeoRange: calculating geographic range from occurrence data. R package version 0.1.0. (2017).

[86] Hahne F, Ivanek R (2016). Visualizing genomic data using Gviz and Bioconductor. Methods Mol. Biol..

[87] Renaud G (2018). Improved de novo genomic assembly for the domestic donkey. Sci. Adv..

[88] Jagannathan V (2019). Comprehensive characterization of horse genome variation by whole-genome sequencing of 88 horses. Anim. Genet..

[89] Andersson LS (2012). Mutations in DMRT3 affect locomotion in horses and spinal circuit function in mice. Nature.

[90] Teufer M (1999). Ein Scheibenknebel aus Dzarkutan (Süduzbekistan). Archäologische Mitteilungen aus Iran und Turan. Band.

[91] Chechushkov, I. V. *Wheel Complex of the Late Bronze Age Era of Steppe and Forest-Steppe Eurasia (from Dnieper to Irtysh)*. PhD thesis. Department of Archeology and Ethnography of the Federal State Budgetary Institution of Science, Institute of History and Archeology of the Ural Branch of the Russian Academy of Sciences (2013).

