# Supplementary material for: The origins and spread of domestic horses from the Western Eurasian steppes
Source: Nature. 2021 Oct 20;598(7882):634–40. doi: 10.1038/s41586-021-04018-9 (PMC8550961; doi:10.1038/s41586-021-04018-9)
Supplement: Supplementary file 1 — Supplementary Information Supplementary Methods; Supplementary Discussion; Supplementary Notes. This file provides full description of archaeological material and contexts, develops the methodology underlying genome analyses, and summarizes linguistic information on Indo-European equine and Indo-Iranian chariotry terminology. A full list of supplementary references is provided. [file 41586_2021_4018_MOESM1_ESM.pdf]

nature portfolio

https://doi.org/10.1038/s41586-021-04018-9

Supplementary information

The origins and spread of domestic horses from the Western Eurasian steppes

In the format provided by the authors and unedited

Supplementary Methods 1. Archaeological sites, dating and samples

Archaeological sites and sample information

The following section is organized alphabetically by country and describes the archaeological contexts associated with all equid remains sequenced in this study (SI Table 1). The full name of each specimen is composed of the sample name, the excavation country (three first letters) and its corresponding age (in years Before Common Era, BCE, if prefixed with 'm', or Common Era, CE, if no prefix is added). Temporal information is estimated from direct radiocarbon dating or inferred from the archaeological context (SI Table 1). It is noteworthy that all specimens previously published in either Fages and colleagues[5] or Gaunitz and colleagues[3] and included in this study have been here re-processed according to the methodology described in the following sections, in order to provide novel sequence data consistently generated using the same experimental procedures as those applied to all other specimens. This, thus, ensured minimal impact of potential technological batch effects.

- **Austria, Bad Pirawarth, 48.451361 N, 16.599992 E:**

The archaeological site of Bad Pirawarth is located in Lower Austria. Excavations revealed settlement remains like several storage pits and a pottery kiln, assigned to the Early Bronze Age[92]. Radiocarbon analyses of three horses (one subadult, two adults) found on site (pers. comm. K. Saliari, E. Pucher), however, indicated that the site was possibly also occupied later than previously expected. The horse specimen labelled SRNHM05_Aus_m585 belongs to this putative later Iron Age occupation.

- **Belgium, Goyet Third Cave, 50.44667 N, 5.00911 E:**

The third cave of Goyet, Belgium, represents a Middle and Upper Palaeolithic site from the Mosan basin. The horse specimen labelled Vert311_Bel_m34314 corresponds to a wild horse, sampled from a metatarsus fragment that was excavated from the first bone horizon[93]. This specimen, with a calibrated age of c. 34,300 y BP, was previously analyzed at the genetic level by Fages and colleagues[5].

- **China, Fengtai, 36.840667 N, 101.958472 E:**

The horse specimen labelled Fen4_Chi_m800 was excavated from the archaeological site of Fengtai, Qinghai province, China, and dates back to the Iron Age. An important number of domesticated plant grains have been unearthed from Fengtai, which indicates a mixed agro-pastoral economy. The genome of this specimen was previously analyzed by Fages and colleagues[5].

- **Czechia, Březno, 50.35784 N, 13.74354 E:**

The horse specimen labelled PRA27x81_Cze_m1241 was sampled from an adult female horse skull, which was found together with a big part of a postcranial skeleton in pit 629 (ID 510), and was allegedly tossed[94]. It belonged to the Late Bronze Age Knovíz Culture settlement of Březno, district Louny, Czechia. No butchering, burning or gnawing marks were observed on the skull. Domestic animals clearly dominated in the Knovíz Culture settlement, with cattle representing the most frequent (50–62% according to various methods[95]), and horses only a more modest proportion (4.5–9.7%). The dimension of the skull analyzed suggests a medium-sized horse, which is expected to be domestic in this time-space context.

- **Czechia, Černý Vůl, 50.14943 N, 14.33203 E:**

The poly-cultural site of Černý Vůl, district Prague-West, Czechia, is located in the lowlands of central Bohemia. The horse specimen labelled PRA32_Cze_m787 is, based on ceramics and C14

analysis, considered to represent an Early Hallstatt intrusion in Neolithic feature 104/1975-1977 (ID 250)[96], as radiocarbon dating of the analyzed horse scapula indicates a temporal locus between the Bronze and Iron Ages. Measurements taken from the scapula suggest a medium size of the horse. This early Hallstatt intrusion could be influenced by Cimmerian invasions in the region.

- **Czechia, Holubice, 50.20132 N, 14.29183 E:**

The horse specimen labelled PRA40_Cze_m2037 comes from the archaeological material present in the feature 2/2005 from site Holubice, district Prague-West, Czechia (ID 146). This is located in the central Bohemia lowlands. According to Daněček[97] and Kovačiková & Daněček[98] the ceramic finds from this feature have been dated to the Funnel Beaker Culture but younger, the Únětice Culture, finds were also found in the vicinity. Only 17 determinable bone finds were present in the pit. The only animal bone that was identified as a horse was investigated in this study.

- **Czechia, Litovice, 50.08088 N, 14.23425 E:**

Excavations at the archaeological site of Litovice (Za Litovickou tvrzí), district Prague-West, Czechia, revealed osteological finds of various cultures[99]. Radiocarbon dating of the horse specimen labelled PRA18_Cze_m3226 corresponds to the Middle Eneolithic horizon (possibly the Salzmünde Culture). This sample originates from a bigger part of an adult male skull without visible butchering, burning or gnawing marks, and is the only find unearthed from the bottom of pit 118/2004 (ID 352)[100]. However, there were no indications of any ritual treatment of this horse. The dimension of the skull analyzed suggests a medium-sized horse.

- **Czechia, Stránská skála, 49.19081 N, 16.67640 E:**

The horse specimen labelled PRA84_Cze_m3236 originates from the mandible of a whole skull found in a small pit situated in the corner of large clay pit at Stránská skála in Brno, Czechia[101], dated to the Funnel Beaker Culture (Early Eneolithic, mid fourth mill BCE) but containing also ceramics of later, Boleráz horizon. The site is located in the South Moravian lowlands. Domestic animals clearly dominated in the osteological material, and cattle were especially frequent (70%). Horses bones, in contrast, were scarce (0.3%). The skull of the individual included in this study is without visible butchering, burning or gnawing marks and represents an exceptionally large horse male, exceeding what is expected to be found in the Neolithic–Eneolithic (157–168cm in withers). This has suggested import or influence from the eastern steppes[102].

- **Czechia, Tištín, 49.31300 N, 17.15314 E:**

The horse specimen labelled PRA62_Cze_m1249 was sampled from the molar of a small horse. It originates from Tištín, district Prostějov, Czechia, in the central Moravian lowlands. It was unearthed from a settlement pit 553/2002 (ID 175312), together with ceramics of the Bell Beaker Culture, by Andrea Matějíčková. Radiocarbon dating, however, placed the sample in the Late Bronze Age. The material in the pit as well as in the whole settlement is dominated by domestic animals, especially cattle.

- **Czechia, Toušeň – Hradišťko, 50.16813 N, 14.72267 E:**

The archaeological site of Toušeň – Hradišťko, district Prague-East, central Bohemia, is located in the lowlands, near the Elbe river. Archaeological excavations supervised by Jaroslav Špaček revealed finds of the Middle Eneolithic, Early Bronze Age, Iron Age and Middle Ages. The premolar of the smaller horse specimen labelled PRA9_Cze_m1845 analyzed here originates from the trench 5/1976 (ID P3416), which cut cultural layers. It was described to be part of a normal settlement waste where domestic animals predominated.

- **Czechia, Tuchoměřice, 50.13493 N, 14.28531 E:**

Tuchoměřice, district Prague-West, Czechia is located in the lowlands of Central Bohemia. The horse specimen labelled PRA64_Cze_m1110 was sampled from the mandible of an old medium-sized horse. It was excavated from feature 36/2005 (ID 428), a regular settlement waste. The find was accompanied by ceramics of the Late Bronze Age Knovíz/Štítary Culture[103]. Sheep/goats, domestic cattle and pigs predominate in the settlement, while the horse represents only 4% of bones[104].

- **Czechia, Tuchoměřice – Kněžívka, 50.12523 N, 14.26973 E:**

Tuchoměřice – Kněžívka, district Prague-West, Czechia, is located in the lowlands of Central Bohemia. The horse specimen labelled PRA68_Cze_m1310 belonged to a very small horse excavated from settlement feature 73/2007 (ID 1176), which also contained the remains of a child and a ceramic bowl, and was dated to the Late Bronze Age Knovíz Culture[105]. Domestic cattle, pigs and sheep/goats predominate in the settlement according to the preliminary observations.

- **Czechia, Vliněves, 50.36361 N, 14.44931 E:**

The poly-cultural site at Vliněves, district Mělník, Central Bohemian lowlands, excavated under the supervision of Petr Limburský, is dominated by finds from exceptionally large Early Bronze Age – Únětice Culture – settlement and cemetery[106]. The horse specimen labelled PRA77_Cze_m624, the only horse found in the settlement feature 8805/2007 (ID 4349)[107] representing a medium-sized specimen, was, however, dated to the Hallstatt Culture by the radiocarbon analysis.

- **Denmark, Ginnerup, 56.41134 N, 10.74481 N:**

Ginnerup is located on a prominent moraine hill, above the northern shore of the now dried-up Kolindsund on Djursland, eastern Jutland, Denmark. The site dates back to the Middle Neolithic, approximately ~3,100 cal. BCE, a transitional phase between the Funnel Beaker Culture and the Pitted Ware Culture in Djursland. It contains cultural layers and several larger oblong pits/ditches forming a series of isolated ditch segments[108]. Features A1, A3 and A4 contained ritual depositions of marine shells, animal bones (including horses), pottery and flints. Small-scale excavations were carried out in 2001 and 2003 and provided the material underlying the horse specimens labelled DJM130x6_Dan_m3011 and DJM613x1_Dan_m2994, both included in this study.

- **Estonia, Asva, 58.410926 N, 23.016137 E:**

The fortified settlement of Asva is located at the eastern site of the Saaremaa Island of western Estonia, 5 kilometers away from the seacoast. During the Late Bronze Age (eighth-sixth centuries BCE), the settlement was spread across a surface area of 3,500 square meters. In total, three different occupation phases could be identified during the excavations from 1938-1939, 1948-1949 and 1965-1966, including a non-fortified settlement, a fortified settlement stage I and a fortified settlement stage II, respectively. The horse specimens labelled Esto2_Est_m661 and Esto3_Est_m659 were unearthed during the excavation campaign of 1939 (Collection ID no. AI-3799:467). The artifacts found at the site consist of ceramics (for a total of approximately ~32,000), bone and horn items (approximately ~500), a few bronze items, arrowheads, sickles, spoons, etc[109]. In addition, several thousand of remains of domestic animals (including horses) and seals have been recorded[110].

- **Estonia, Ridala, 58.4552 N, 23.0326 E:**

Ridala is a fortified settlement on the Saaremaa Island of western Estonia, and dated to the eight-seventh centuries BCE[111]. At least eight slaughtered horses have been excavated from this Late Bronze Age site, including the horse specimens labelled Esto8_Est_m654, Rid1_Est_m700 and

Rid2_Est_m700 analyzed in this study (excavated in 1961-1963, collection ID no. AI-4261 and AI-4329). The two latter specimens were previously analyzed at the genetic level[3,5].

- **France, Le Closeau, 48.871883 N, 2.157139 E:**

Le Closeau is an Epipaleolithic site located in Rueil-Malmaison, northern France, specifically dating back to the Early Azilian period[112,113]. Individual Closeau3_Fra_m10400 was sampled from locus 46, from which a wide variety of animals have been excavated, including horses that were most likely hunted all year long[114,115]. Excavations at locus 46 also revealed the presence of deers, boars, and even some hares, dogs and cave lions[116].

- **France, Étiolles, l'amas de cheval, 48.63324 N, 2.465916 E:**

Etiolles is an Upper Palaeolithic site located by the Seine river, France, and associated with the Bølling interstadial period. Excavations at Etiolles have revealed many faunal remains, and identified at least one bison, one mammoth, one reindeer and three horses that were most likely hunted by Magdalenians. These horses include one adult, one subadult and one juvenile, possibly corresponding to a family group composed of an older stallion, a young mare and their foal[114]. Samples Etiolles4_Fra_m13759, Etiolles6_Fra_m13608, Etiolles7_Fra_m13815 and Etiollesx8_Fra_m13693 were all excavated from partly-fragmented petrosal bones and seem to correspond to the fore-mentioned family.

- **France, Igue du Gral, 44.499039 N, 1.692232 E:**

L'Igue du Gral is a 9-meter deep late Pleistocene natural trap-cave located at Sauliac-sur-Célé, in southern France, and excavated since 2001. It covers a long palaeontological period, covering 10,500 BP to 32,000 BP, and has yielded over 26,000 animal remains, mostly reindeers, horses, bison for large herbivores but also many leporids, choughs and carnivores[117]. All wild horses from Igue du Gral included in this study were sampled from well-preserved petrosal bones.

- **France, La Monédière, 43.3667 N, 3.4167 E:**

La Monédière is an archaeological site located in the commune of Bessan, Hérault, France, excavated from 1972 to 1974. Because of its proximity to the Greek counter of Agde, it is thought to have developed as a flourishing Mediterranean trade center between the sixth and the fifth century BCE. The presence lots of Greek material and apses in the excavated infrastructures suggest that La Monédière was most likely a Phocaean town, and could thus represent a Greek attempt at colonising the region further inlands[118]. Specimen UE10287_Fra_m617 was sampled from a tooth unearthed while cleaning the surface of the uppermost layer of the site.

- **France, Tureau des Gardes 6, 48.387245 N, 3.007653 E:**

Le Tureau des Gardes 6 is a Magdalenian site located close to Marolles-sur-Seine, France. Excavations have revealed at least eight reindeers and 22 horse individuals, among which specimen BPTDG1_Fra_m11800. This site likely represented a natural trap, used by hunters especially around the end of winter and start of springtime. The presence of ten foals - under the age of two - out of the 22 excavated horses suggests that hunters specifically targeted younger individuals while hunting[114].

- **Georgia, Tachti Perda, 41.467 N, 46.017 E:**

Tachti Perda is a prehistoric settlement, located in Kakheti, eastern Georgia. It was occupied from the late middle Bronze Age until the late Iron Age[119]. The horse specimen labelled TP4_Geo_m1578 was excavated from this site and dated to the late Bronze Age. The specimen was previously analyzed at the genetic level by Gaunitz and colleagues[3].

- **Germany, Hohler Stein bei Schwabthal, 50.06053 N, 11.104783 E:**

The "Hohler Stein" is a conspicuous dolomite rock about 2-3 kilometers away from Schwabthal, North Bavaria, Germany. Excavations have revealed indications of a late Neolithic occupation associated with the Corded Ware Culture, as well as ritual activities from many prehistoric periods, from the early Neolithic to the Iron Age[120]. The horse bones Hohler1x1_Ger_m2679, Hohler1x2_Ger_m2681, Hohler2_Ger_m2719, Hohler3x1_Ger_m2676, Hohler3x2_Ger_m2681 and Hohler3x3_Ger_m2681 were excavated from different trenches around the rock and can be interpreted as waste disposal or alternatively ritual deposits from the nearby settlement. These bones belong to subadult and adult horse individuals, and have been radiocarbon dated to between 2,700 and 2,500 BCE. The ceramics of the final Neolithic period have been described as typical of the Corded Ware Culture, which suggests that the "Hohler Stein" settlement is comparable to other nearby Corded Ware Culture settlements of the same region, e.g. Wattendorf- Motzenstein[121,122] or "Rothensteine" near Stübig[123].

- **Hungary, Dunaújváros-Kosziderpadlás, 46.961906 N, 18.935523 E:**

The settlement of Dunaújváros-Kosziderpadlás is a Middle Bronze Age site located in Hungary, by the Danube river. A large number of domestic animals and bronze hoards were found at this site[124], suggesting that the site represented an important trade center for livestock and bronze[125]. In particular, over 150 horse specimens were excavated, including the horse specimen labelled Duk2_Hun_m2059. The genome of this specimen was previously analyzed by Gaunitz and colleagues[3] and by Fages and colleagues[5].

- **Hungary, Kaposújlak-Várdomb, 46.362 N, 17.731 E:**

Kaposújlak-Várdomb is located in South-West Hungary and encompasses different time periods, from the late Neolithic until the late Bronze Age. In the Early Bronze Age, the site represented the largest Somogyvár-Vinkovci fortified settlement in Hungary, and probably served as a butchery site as most of the animal remains from that period represent food waste[126]. The animal record from that period is dominated by cattle and pigs, with horses representing less than 1% of the total assemblage. Horse sample CAR05_Hun_m3130 was found in feature 1038, which contained the remains of both domestic and wild animals, with most of them representing skeletons and separated skulls. Based on archaeological and morphological analyses, the Early Bronze Age horse remains of this feature have been generally assigned to the domestic form.

- **Iran, Tepe Hasanlu, 37.004486 N, 45.458817 E:**

A total of eight horses included in this study were excavated from Tepe Hasanlu, a fortified site in western Azerbaijan province, Iran. Human occupation at Tepe Hasanlu span a long time period, from the Neolithic up until the Iron Age[127]. The individuals analyzed here were unearthed from various archaeological parts of the site, including rough soil deposit, a citadel, and horse stables, but all date back to the Iron Age[128]. One specimen, Hasanlu2405_Ira_m868, and three other specimens, Hasanlu3394_Ira_m790, Hasanlu3461_Ira_m913 and Hasanlu368_Ira_m878 were previously analyzed at the genetic level by Gaunitz and colleagues[3] and Fages and colleagues[5], respectively.

- **Iran, Sagzabad, 35.773 N, 49.938 E:**

Sagzabad is an archaeological site located in the Qazvin plain, Iran, and was occupied in the Late Bronze Age and Iron Age. More than 10,000 animal bones were excavated at Sagzabad, representing mostly domestic cattle and ovi-caprines, but also to a lesser but significant extent, domestic horses, including the horse specimen labelled SAGS27_Ira_m1102[129]. This specimen was previously analyzed at the genetic level by Fages and colleagues[5].

- **Italy, Tarquinia, 42.249167 N, 11.756111 E:**

The 'monumental complex' of Tarquinia offers the extraordinary opportunity to monitor the cultural development of an Etruscan area sacred to the major female goddess of the Etruscans. Archaeological evidence sheds light on the continuity and memory of the sacred area over the centuries up to the encounter with Rome. From the end of the 10th century BCE, offerings located by a natural cavity show the cult of a divinity of Nature, who catalyzed the very first community. Ritual sealing of a number of votive pits of different size contain a considerable number of animal bones[130]. The sample Tarquinia3298_Ita_m657 analyzed here probably corresponds to a secondary deposit.

- **Kazakhstan , Ashchisu, 49.916667 N, 74.216667 E:**

The Ashchisu kurgan is located in the Bukharzhirau district of the Karaganda region of the Republic of Kazakhstan, and dates back to the early Alakul Culture (19th-18th centuries BCE). A total of seven altars were discovered next to the graves. The first altar included a vessel made of clay and a horse's skull, while altars 2, 3, 4, 5, 6, and 7 consisted of skulls and horse bones, including the horse remain here referred to as PKo7_Kaz_m1754[131].

- **Kazakhstan, Belkaragay, 51.52 N, 62.55 E:**

The horse specimens labelled NB13_Kaz_m3012 and NB15_Kaz_m3150 were unearthed at Belkaragay, an Eneolithic site in Kostanay region, Kazakhstan, and associated with the Botai-Tersek Culture. More than 20,000 artifacts and goods were recovered from this site, including stone tools, ceramics and animal bones. The faunal assemblage at Belkaragay includes carnivores, such as wolves and foxes, but also Saiga antelopes and equids, including hemiones and horses, which represent the vast majority of the animal remains and belong to at least 139 individuals.

- **Kazakhstan, Bestamak, 51.575646 N, 64.724468 E:**

The Bestamak cemetery is located at the source of the Ubagan River, Kostanay region, northern Kazakhstan. The total excavation includes 183 burial pits and ritual structures. It represents the only site in Kazakhstan containing materials dating from the Eneolithic Period to the Middle Ages, inclusively[132]. The horse specimen labelled Besta5_Kaz_m1892 was unearthed from *Sacrificial Complex 127*, which is an oval pit, starting from a depth of 0.2 m, where skulls of 1 ox, 9 cows and 2 horses were recorded. All skulls have traces of breaks. A second layer of animal limbs was found under the skulls. This sacrificial complex is ascribed to the Sintashta Culture (Bronze Age, second mill BCE). The horse specimen labelled Besta7_Kaz_m3132 was sampled from a horse skull excavated from *Pit 142*, appeared also to be associated with the Sintashta Culture, but has now been radiocarbon dated as belonging to the earlier Eneolithic Tersek phases of the site. The skull was found at a depth of 1.8 meters, along with several fragments of animal bones. The horse specimen labelled Besta6_Kaz_m661 was found in *Burial 183*, at the depth of 1.7 meters, together with a 30-year-old woman buried in an elongated position on her back, with her head to the North-West. This burial dates to the Early Iron Age.

- **Kazakhstan, Borly, 51.826192 N, 77.948253 E:**

The Borly settlement site is located on the western shore of the eponymous lake, 35 kilometers East of the River Irtysh, dating back to the middle to second half of the fifth mill BCE. It is the largest Neolithic monument in the territory of North-East Kazakhstan, with more than 400 square meters of cultural layer studied[133]. Archaeological material includes stone and bone tools and ceramics of the Neolithic period, as well as a large number of horse and cattle bones, including samples Borly1_Kaz_m4449, Borly3_Kaz_m4499, Borly7_Kaz_m4636 and Borly8_Kaz_m4290.

- **Kazakhstan, Borly 4, 51.824694 N, 77.945111 E:**

The Borly 4 settlement is located in the Lebyazhinsky district of the Pavlodar region of the Republic of Kazakhstan, 35 kilometers East of the River Irtysh, on the western shore of lake Borly. The settlement contained three layers, including (1) an upper black-brown layer containing mixed archaeological materials from the modern Soviet period to the Early Bronze Age, (2) a rich Eneolithic layer with many animal bones, and (3) a light brown layer with materials of the Middle and Late Neolithic were found. The horse specimens labelled PAVH8_Kaz_m2961 and PAVH9_Kaz_m2959 both belonged to the Eneolithic layer. These specimens were previously analyzed at the genetic level by Gaunitz and colleagues[3].

- **Kazakhstan, Botai, 53.303942 N, 67.645786 E:**

Botai is an Eneolithic settlement in northern Kazakhstan dating back to ~3,500 BCE, and associated with the eponymous semi-sedentary Botai Culture, the subsistence economy of which relied heavily on horse management. Archaeological and molecular evidence suggest that horses were domesticated at Botai, but that these are not the direct ancestors of modern domesticated horses[3,5]. A total of 23 ancient horses from Botai were included in this study, of which 14 were not previously sequenced.

- **Kazakhstan, Halvai, 52.843 N, 62.9029 E:**

The Halvai kurgans are located on the left bank of the Tobol branch of the Karatomar reservoir, Kostanay region, northern Kazakhstan. Samples Halvai2_Kaz_m1856 and Halvai3_Kaz_m1856 are from Halvai Kurgan 3 and were unearthed from Burial 3. The burial was covered with overlapping pine logs, above which were two horse skulls from a stallion and a mare, and in the South-East corner, another mare's skull. Only the southern part of the pit remained undisturbed, where two human skulls were found along with a bronze awl, a knife in a scabbard made of fabric, leather and birch bark and an axe. At least three adult individuals and one child were buried, including two women and a man[134]. Horse specimen KSH5_Kaz_m1895 was excavated from Pit 8A, - which partially overlaps with Pit 8 - with the remains of a woman and a sheep.

Finally, specimen KSH4_Kaz_m1955 was excavated from Halvai Kurgan 5, 500m to the North-East of Halvai 3, from Pit 4. Pit 4 is the central burial of the kurgan and is 5 x 4.8 m with a depth of 3.3 m. The pit contained the disparate bones of a woman aged 30-40 years, along with the remains of cereals, the skull of a horse, other bones of horse and sheep, stone points, a bronze axe-adze, fragments of ceramic and a zoomorphic alter stone. The burial dates to the Bronze Age Sintashta Culture, but was robbed in antiquity. Specimen KSH4_Kaz_m1955 specimens were previously analyzed at the genetic level by Fages and colleagues[5].

- **Kazakhstan, Kent, 49.202561 N, 75.940728 E:**

Kent is one of the most studied settlements of the Begazy-Dandybaev Culture, dating from the 14-10 centuries BCE, and located 220 kilometers to the South-East of Karaganda, in the Kent mountain range, Kazakhstan. Having emerged as a settlement supporting a large population, Kent quickly grew and became a major cultural, economic and administrative center. On the eastern outskirts of the monument were three large stone-walled enclosures with traces of bonfires and the remains of ritual offerings in the form of bronze and carved bones, suggesting they were places of worship. The archaeological evidence also indicates a significant concern within the population for warfare. This settlement was included in the system of intercultural relations, as evidenced by finds of imported ceramics indicating a multi-ethnic composition of the population. The principal fauna at Kent were sheep/goat - 51.5%, cattle - 26.74% and horses - 18.42%, including samples Kent1_Kaz_m1466, Kent2_Kaz_m1474, Kent5_Kaz_m1494, Kent6_Kaz_m1463 and Kent7_Kaz_m1476. Wild animals were also hunted, including wild aurochs, gazelle, saiga antelope and deer. The

material culture and faunal evidence clearly indicate that cattle breeding was the main activity in the subsistence economy of the inhabitants of Kent.

- **Kazakhstan, Kozhai 1, 49.4 N, 66.75 E:**

Kozhai 1 is a settlement site of the Eneolithic Tersek Culture, located centrally within Kazakhstan in the far South of Kustanay region[135,136]. The Tersek Culture bears many similarities to the contemporary and neighboring Botai Culture with horses dominating faunal assemblages and representing the mainstay of the subsistence economy. Whilst the Botai exploited little else, Tersek sites have slightly more diversity in their animal bone assemblages. At Kozhai 1, from approximately ~70,000 identified bone specimens, about 66.1% were horses - including specimens Kozhai_Kaz_m3342 and UR17x80_Kaz_m3235, 21.8% onagers, 9.4% bisons and 2.1% cattle[137]. The domestic or wild status of horses at Kozhai has been debated and still remains contentious.

- **Kazakhstan, Krasnyi Yar, 53.325935 N, 69.260924 E:**

The horse specimen labelled KY001_Kaz_m3244 was recovered from Krasnyi Yar, an Eneolithic site associated with the Botai Culture, the economy of which relied on horse management. Similarly to Botai, horses at Krasnyi Yar were likely to be domesticated, and kept in corral enclosures for milk and meat consumption primarily, although excavated horse leather and bone tools suggest the use of secondary products as well[2,4].

- **Kazakhstan, Michuruno 1, 52.447098 N, 76.794851 E:**

The horse specimen labelled Mich_Kaz_m2019 was excavated at Michurino 1, a multi-layered settlement of the Early Bronze Age to Early Iron Age, located 3 kilometers South of Michurino village in the Pavlodar region. From 1995 to 1997 and in 2015, an area of 346 square meters was excavated, and the remains of man-made structures, burials, animal bones, ceramics and metal objects of the early Bronze Age were discovered. They belong to a later stage, corresponding to the Yelunin Culture. Based upon analogues and radiocarbon dates, the lifetime of the monument appears to span the transition from the third-to-second mill BCE[138].

- **Kazakhstan, Novoil'inovskiy 2 Cemetery, 52.6401793 N, 62.7610338 E:**

The horse remains Novoil1_Kaz_m1832 and Novoil2_Kaz_m1832 were excavated at the Novoil'inovskiy 2 Cemetery, Kazakhstan, which provided some of the earliest evidence for established horsemanship practices in Central Asia steppes. Radiocarbon dating returned 1,890-1,774 BCE for the age of the complex, and provided, thus, an antequem date for the rise of chariot or horseback riding in the region[139].

- **Kazakhstan, Shiderty 3, 51.639256 N, 74.654947 E:**

The multi-layered site of Shiderty 3 is located on the north-eastern outskirts of the Kazakh Uplands. There were six cultural layers containing up to nine horizons with various types of stone industries dating from the end of the Palaeolithic to the Early Bronze Age. Until recently the lack of radiocarbon dating from layer 6 did not allow a precise determination of the earliest phase. The horse specimen labelled Shid1_Kaz_m10458, obtained from the tooth of a horse from layer 5, was dated to the eleventh mill BCE, while its estimated date was previously determined by stratigraphy to be at the end of the pre-boreal/beginning of the boreal, at the boundary of the eight-seventh mill BCE[140]. The new radiocarbon evidence therefore compels to reconsider the dating of layers 5 and 6, and in the future, to conduct additional studies to clarify their age.

- **Kazakhstan, Shilikty 5, 47.237169 N, 84.542033 E:**

Shilikty 5 is an archaeological site located in the Zaysan district of East Kazakhstan, North-West of the village of Tasbatau, and dated to the fourth century BCE. The horse specimen labelled KZ2019x1a_Kaz_m293 was unearthed from a grave pit, which also contained the remains of a man. The horse laid on its left side, head to the North, and is associated with the funerary rite of the ancient nomads of the region.

- **Moldova, Gordinesti II-Stînca goală, 48.159923 N, 27.169232 E:**

Gordinesti II-Stînca goală is a Late Eneolithic settlement, in Edineț district, Moldova. Excavations at this site yielded only a few wild animals, the majority of the remains being domestic cattle and goats. Interestingly, a fragmented and burnt tooth of a juvenile horse, here referred to as MOLDA1_Mol_m2063, was also unearthed from the cultural layer of the site. Morphological analysis of the horse tooth suggested that this specimen was domestic[141].

- **Moldova, Miciurin, 47.987105 N, 27.788329 E:**

Miciurin is a Middle/Late Bronze Age settlement located near Odaia, Moldova, and associated with the Noua Culture. Horse remains found at Miciurin, including sample Miciurin05_Mol_m1373, represent approximately ~4% of the animal assemblage recovered from ashy deposits, with cattle and sheep/goat forming the largest part of the excavated materials[142]. The clear dominance of domestic species was the main reason to assign the horse remains to the domestic horse.

- **Mongolia, Bor Shoroonii Am, 46.4 N, 100.68 E:**

The site of Bor Shoroonii Am is a large, multicomponent cemetery complex in the southern Khangai Mountains of Bayankhongor province, Central Mongolia. The site contains burials and monuments ranging from the late Bronze Age through the early Middle Ages. The Deer Stone and Khirigsuur component of the site belongs to the Late Bronze Age Deer Stone-Khirigsuur (DSK) Complex (~1,200-700 BCE), which represents the first culture group associated with horse breeding and horse transport in Mongolia. The horse specimen labelled M17x127_Mon_m658 is a young individual, found in a slab-grave like feature containing an uprooted and reburied Deer Stone. Within the feature were several other juvenile horses and an elderly female horse, and a large number of other livestock. Although it was disturbed, the presence of human remains indicates that this feature was a burial.

- **Mongolia, Burgast, 49.830833 N, 89.99 E:**

The site of Burgast is located in Mongolia, in the province of Bayan-Ölgii (district of Nogoonnuur), on the eastern fringes of the Altai mountain range. Archaeological remains from several periods (Late Bronze Age, hun-Sarmatian, Türk) are scattered along the terrace, and include a Late Bronze Age khirigsuur. To the East, and aligned over a length of 30 meters, there are seven mounds covering horse remains, sometimes showing connected cervical vertebrae and terminal phalanges, including sample GVA9042_Mon_m1068.

- **Mongolia, Ganga-Tsagaan-ereg, 49.572897 N, 103.251933 E:**

Ganga Tsagaan ereg is an archaeological site consisting of seven burial mounds, located in the northern Mongolia and dating back to the Late Bronze Age. It is associated with the early stage of the khirigsuur and Deer Stone Culture, however, no "deer" stones were found on site[143-145]. This and other similar sites most likely reflect the existence of a large cultural and historical community in the form of one of the most archaic nomadic empires[146]. The horse specimens labelled Ganx4_Mon_m775 and Ganx10_Mon_m775 were excavated from two altars surrounding two distinct khirigsuurs.

- **Mongolia, Khantain-tov, 49.56105 N, 103.2428 E:**

The horse specimen labelled Hantx9_Mon_m775 was excavated from a stone altar of the Khantain tov archaeological site, located in the northern Mongolia, and associated with the khirigsuur and Deer Stone Culture. The stone altar was located next to a large burial mound, together with 57 other similar altars and some stone memorials[144].

- **Mongolia, Monostoy-Nuga, 49.553253 N, 103.277861 E:**

Monostoy-Nuga is an archaeological site located in the valley of the Egiin Gol River, northern Mongolia, consisting of three burial mounds. Similar to Ganga Tsagaan ereg and Khantain tov sites, it is associated with the Khereksur and Deer Stone Culture, although no "deer" stones were found at the Monostoy-Nuga site[143,145]. Horse bones come from one burial mound of this site. This khirigsuur consists of an almost square fence made of stones, with central embankment under which one individual was buried, as well as seven small stone altars to the South, and three more to the West, from which samples Monx2_Mon_m1100, Monx4_Mon_m1018, Monx7_Mon_m1026 and Monx9_Mon_m775 were unearthed[144].

- **Mongolia, Morin Mort, 46.94125 N, 98.595817 E:**

The site of Morin Mort is a Deer Stone monument site in the southern Khangai Mountains of Bayankhongor province, Central Mongolia. The Deer Stones are situated in a small valley bottom adjacent to a hill with a rock panel showing dozens or hundreds of horse hoof prints carved into the hillside, from which the site derives its name. The horse specimen labelled M17x199_Mon_m865 comes from a small stone mound encircling a Deer Stone of the "*Sayan Altai*" type, and is an elderly female horse, with osteological evidence of use in transportation.

- **Mongolia, Tsatsyn Ereg, 47.750843 N, 101.369458 E:**

The site of Tsatsyn Ereg is located in the Khoid Tamir Valley, Arkhangaï province, Mongolia. Its total area encompassed 150 squared kilometers, in which are concentrated many structures of different periods, most notably in the Bronze Age. Among the excavated structures, a large Khirigsuur and features associated with a Deer Stone yielded a large number of horse bones[147]. The three specimens from Tsatsiin Ereg included in this study, and labelled GVA9010_Mon_m1057, GVA9035_Mon_m1024, GVA9036_Mon_m1013 and GVA9037_Mon_m1013, come from a Deer Stone associated with a total of 144 mounds.

- **Mongolia, Ulaan Tolgoi, 49.931778 N, 99.804167 E:**

The site of Ulaan Tolgoi, sometimes also known as Erkhel Lake, is a large Deer Stone and Khirigsuur monument complex on the southern shores of Erkhel Lake in Khuvsgul Province, northern Mongolia. Excavations of this site have revealed the evidence of a fully developed diary pastoral economy. The excavated horse here referred to as M17x126x1_Mon_m942 comes from a small stone mound encircling Deer Stone 5, and is a juvenile horse.

- **Mongolia, Ushgiin Uvur (Ushkin-Uver), 49.655567 N, 99.9271 E:**

Ushgiin Uvur (Ushkin-Uver) is an archaeological site of the transition period from the Bronze Age to the Early Iron Age (10th – 7th centuries BCE), located next to the Ulaan Uushig Mountain in the northern Mongolia. This large memorial complex belongs to the Khirigsuur and Deer Stone Culture[148]. "Deer" stones were placed instead of the burials of warriors. They contain images of weapons belonging to the end of the Bronze Age - Early Scythian time Nearby were a large number of altars in which skulls and limb bones of sacrificial horses were found, including the specimen designated in this article as Ushx9_Rus_m775[148,149].

- **Mongolia, Zeerdegchingiin Khoshuu, 51.421361 N, 99.364611 E:**

The site of Zeerdegchingiin Khoshuu is a Deer Stone-Khirigsuur site located at the far northern edge of Tsagaan Nuur lake in the Darkhad Basin of Khuvsgul province, northern Mongolia. The complex consists of a small Khirigsuur surrounded by a row of horse burials and stone circles. Excavations in 2015 revealed a bronze knife inside the Feature A horse mound, one of the first artifacts ever found in direct association with horse remains, including sample M17x159_Mon_m1136. This is the only Deer Stone site to contain an in-situ artifact besides horse/human bone.

- **Mongolia, Zunii Gol, 49.309444 N, 99.849722 E:**

The horse specimen labelled M17x172_Mon_m987, M17x174_Mon_m1145 and M17x176_Mon_m939 were excavated from the site of Zunii Gol, a large Deer Stone and Khirigsuur burial complex located near the town of Tumurbulag in Khuvsgul Province, northern Mongolia. The site belongs to the late Bronze Age Deer Stone-Khirigsuur Complex (~1,200-700 BCE), which represents the first culture group associated with horse breeding and horse transport in Mongolia. The site is known for its unique and beautiful Deer Stones, which include not only the classic Mongolian deer but also the beginnings of other elements of Scythian "*animal style*" art, including predators, birds, and even a frog.

- **Mongolia, Zuunkhangai, 49.325583 N, 95.442278 E:**

The site of Zuunkhangai, is a Deer Stone-Khirigsuur burial complex located near the township of Zuunkhangai in Uvs province, northern Mongolia. The excavated horses M17x140x1_Mon_m1116 and M17x188_Mon_m1010 come from two small burial mounds outside of several Khirigsuurs, located at the same cemetery.

- **Poland, Kazimierza Wielka, 50.2741 N, 20.4654 E:**

Samples PLKaz1_Pol_m1550, PLKaz2_Pol_m1480, PLKaz3_Pol_m1550, PLKaz4_Pol_m1550, PLKaz5_Pol_m1550 and PLKaz6_Pol_m1550 were recovered from excavations in the city of Kazimierza Wileka, site number 6, Świętokrzyskie province, Poland. The archaeological excavations were conducted in 2018 and revealed that the remains date back to the Early Bronze Age and are associated with the Trzciniec Culture. All six horses from Kazimierza Wielka included in this study were unearthed from a single pit (number 8), without any other archaeological material, and were accompanied by three features filled by horse and some single cattle bones, with Trzciniec Culture pottery[150]. Since these horses come from a Trzciniec characteristic horse burial, they were most likely all domesticated.

- **Poland, Miechów, 50.35749 N, 20.04609 E:**

The archaeological site number 3 in Miechów lies on the left bank of the upper Vistula River, southern Poland. The site is located in the Miechów Upland, and is part of the Nida Basin. The salvage archaeological fieldwork was carried out between 2011 and 2012[151], and more than 3,000 archaeological features were found. They dated from the Early Neolithic until the Early Medieval period. The horse specimen labelled PLMie8_Pol_m649 was sampled from a partially preserved skeleton of a domestic horse, unearthed from pit 2077, and associated with the Early Bronze Age Trzciniec Culture. Specimen PLMie18_Pol_m1483 was sampled from a metacarpus and appeared to be much older as it was both excavated from a single pit containing pottery of the Eneolithic Funnel Beaker Culture together with other animal remains, and radiocarbon dated to ~1,483 BCE. Radiocarbon dating of both specimens did not, thus, appear in line with their stratigraphic association.

- **Poland, Mozgawa, 50.44378 N, 20.51853 E:**

The Mozgawa site is situated in the Nida Basin, on the eastern outskirts of the western Lesser Poland loess upland, and is associated with the Eneolthic Funnel Beaker Culture[152,153]. First archaeological excavations at this site were carried out in 2014-2016 and trenches were identified in three parts of the site[154]. The two horses from Mozgawa included in this study, PLMoz11_Pol_m3505 and PLMoz1x1_Pol_m3801, correspond to horse molar and metacarpus discovered in fillings of feature 11 and 55, respectively, which had lost their original residential/warehouse function and had been left filled. Animal remains here present mostly post-consumer waste, with bones being strongly fragmented, and some even displaying traces of human activity (fire and cut marks).

- **Poland, Pielgrzymowice, 50.143 N, 20.04672 E:**

The archaeological site number 6 in Pielgrzymowice is situated at the southern part of Miechów Upland, 10 kilometers from Kraków. Salvage excavations were carried out from 2016 to 2018 and indicated a multicultural site, settled from the Early Neolithic up until the Early Medieval period. In pit 787, excavations revealed a partially preserved skeleton of a domestic horse, here referred to as PLPie2_Pol_m1532, probably corresponding to a horse burial damaged by later settlers, and associated with the Early Bronze Age Trzciniec Culture.

- **Portugal, Zambujal, 39.0744 N, -9.2857 E:**

Zambujal is an Eneolithic fortified settlement associated with the Bell Beaker Culture, located in southern Portugal. Material excavated at Zambujal consists mostly of domestic animals. However, sample Zam9_Por_m2559, unearthed from trench 15, belongs to the early phase of the site and could be either a domestic or a wild horse.

- **Romania, Căscioarele, 44.12731 N, 26.4696 E:**

Căscioarele is located on an islet, in the gulf of the former Cătălui lake, in the vicinity of Căscioarele, Romania. The tell consists of neo-Eneolithic cultural layers[155] mostly associated with the Gumelnița Culture (4,500-3,900 BCE) but the archaeologists discovered also some artefacts from Cernavoda I Culture. The Gumelnița Culture yielded an abundant faunal assemblage[156], in which wild animals represent about ~80% of the total faunal remains. In particular, large equids, including samples ROCAS09_Rom_m4297 and ROCAS10_Rom_m4309, represent ~8% of the assemblage and most likely correspond to wild individuals. Another sample who indicated also a large equid wild was dated to Cernavoda I Culture, ROCAS12_Rom_m3795.

- **Romania, Gârbovăț (Gîrbovăț), 44.86699 N, 22.007939 E:**

Gârbovăț is an archaeological site associated with the Bronze Age Noua Culture, and located in eastern Romania, in the vicinity of Tecuci. Between 1960 and 1962, excavations at Gârbovăț revealed an abundant domestic faunal assemblage dominated by cattle, goat and sheep, but also including horses, such as the horse remain here referred to as Gar3_Rom_m1539[157]. The specimen was previously analyzed at the genetic level by Gaunitz and colleagues[3].

- **Romania, Nandru Peștera Curată, 45.807945 N, 22.812173 E:**

The horse specimen labelled RONPC06_Rom_m34801 is a wild horse excavated from Nandru Peștera Curată, a Palaeolithic cave located in a crystalline limestone, East/North-East of the Poiana Ruscă Mountains, on the right side of the Valea Roatei stream, Romania[158]. Radiocarbon dating of this sample confirmed the wild status of this animal, which lived some 37 thousand years ago.

- **Romania, Pietrele, 44.067779 N, 26.156737 E:**

Pietrele is an Eneolithic tell-settlement, located in southern Romania, in the vicinity of the Danube river. It was occupied from ~4,550 BCE up until ~ 4,250 cal. BCE. The faunal assemblage at Pietrele includes both domesticated animals - cattle, sheep and goat - and wild game - mostly boar, but also deer, auroch and horse[159]. The horse specimen labelled Pie13_Rom_m4425 was sampled from a bone associated with the later Gumelnița phase of the site, after 4,400 cal. BCE, and most likely belonged to a wild animal.

- **Russia, Aleksandrovskoe IV, 53.974236 N, 74.884425 E:**

The Aleksandrovskoe IV settlement is located on the terrace of the Irtysh River, Russia. Hand sculpted ceramics of the Eneolithic period and stone tools were found in the cultural layer, yet the archaeological culture is still not precisely defined. As for the fauna fossil record, a total of 356 horse bones, 18 aurochs bones and seven saiga bones have been excavated, including samples UR17x67_Rus_m2774, UR17x69_Rus_m2774, UR17x70_Rus_m2753 and UR17x71_Rus_m2790.

- **Russia, Algay, 50.156028 N, 48.526306 E:**

The Algay site is located in the Volga-Ural interfluve, on the right bank of the Bolshoi Uzen' river, Russia. It contains Eneolithic and Neolithic layers, including an upper layer associated with the Khvalynsk Culture and a lower layer that relates to Orlovka Culture. Sample LOR18x29_Rus_m5406 was unearthed from the upper part of the lower layer, related to the middle Orlovka of the Neolithic period. It most likely represents a wild horse. Apart from dogs, all animals from this layer were also wild (wild ox, kulan, fox, saiga, hare, deer, badger).

- **Russia, Arzhan I and II, 52.066389 N, 93.5225 E:**

Samples Arz1x12_Rus_m854, Arz1x13_Rus_m581, Arz1x14_Rus_m800 and Arz15_Rus_m650 were excavated from the huge mound Arzhan-1 (the Tyva Republic, Russia) associated with the Aldy-Bel Culture and dates back to the end of the nineth-to-eighth centuries BCE (Early Scythian time)[160,161]. The Arzhan complex includes weapons, harness, decorations and items in the Scythian-Siberian animal style. The art of this culture is characterized by the image of animals in a static pose[162].

- **Russia, Aygurskiy 2, 45.69 N, 43.262 E:**

The Aygurskiy burial mound cemetery 2 was situated on a promontory overlooking the small river of Aygurki, about 22 kilometers East of the city of Ipatovo, Stavropol region, Russia. The area is part of the Kalaus drainage system, an important linkage of the Caucasus mountain system and the South Russian steppe. The horse specimen labelled BZNK1002x4_Rus_m3450 stems from grave 16 of mound 22, which was the largest of these mounds. This mound comprised three major mound construction phases of which the first was a complex stone construction dating to the Maykop epoch[163]. As for grave 16, it included both human and animal bones, dated to the mid to late Maykop horizon[164]. The material was found during the excavation '*Nasledie*' 2000, Stavropol, licence №2000-776 (V. A. Babenko).

- **Russia, Batagai, 67.567 N, 134.767 E:**

Individual Batagai_Rus_m3136 represents a wild horse, and was excavated from Batagai, Verkhoyansk District, Russia. The specimen was previously analyzed at the genetic level by Librado and colleagues[15].

- **Russia, Bijke-V, 51.164483 N, 86.139367 E:**

Bijke-V is an archaeological site of the Arzhan-Mayemir time (nineth-to-seventh centuries BCE) located on the right bank of the Katun River, 5.6 kilometers from the village of Elanda (Altai Republic, Russia). It is associated with the Bijke Culture, whose subsistence economy mostly relied on horse and sheep breeding. The horse labelled Bi5x1_Rus_m625 was excavated from kurgan 8, together with artifacts related to horse equipment[165,166].

- **Russia, Bol'shekaraganskii, 52.65 N, 59.566667 E:**

Bol'shekaraganskii is located on the first floodplain terrace of the Bolshaya Karaganka River, in southern Russia. The burial ground consists of 24 mounds, each of which containing human burials, as well as clay vessels, bronze weapons, tools and jewelry. This funerary site also includes skeletal parts and entire skeletons of many sacrificed domestic animals: cattle, sheep, goat, horses, pigs and dogs[167]. Samples UR17x1_Rus_m1868 was excavated from mound 11 and samples UR17x4_Rus_m1820 and UR17x5_Rus_m1951 from mound 24, all associated with the Sintashta Culture. The horse specimen labelled UR17x3_Rus_m1815 was found in a grave pit of mound 20, which belongs to the Srubnaya Culture.

- **Russia, Burial mound at Berezovaya mountain, 52.429072 N, 55.227878 E:**

The burial mound at Berezovaya mountain is associated with the Sintastha Culture and located in the Orenburg Oblast, Russia. Two sacrificed domestic horses, including sample LOR18x62_Rus_m1849, were found in this burial together with a chariot[168].

- **Russia, Chinge-Tey-I, 52.067083 N, 93.464806 E:**

Burial and memorial complex Chinge-Tey I is an elite burial mound, surrounded by a moat as well as numerous ritual facings and fences. It is located in the Tyva Republic (Russia) and belongs to the Aldy-Bel Culture of the Early Scythian time. The horse specimen labelled Chin1x1_Rus_m810 was sampled from a tooth recovered from a ditch of this site[169,170].

- **Russia, Choburak-I, 51.173733 N, 86.113083 E:**

Choburak-I is situated on the right bank of the Katun River, 3.4 kilometers South/South-East from the mouth of the Tytkesken River (Altai Republic, Russia). The site is associated with the Iron Age Biyke Culture. The horse specimen labelled Cho1x3_Rus_m625 was excavated from a small stone layout located to the East of the mound 55. It was found deposited on its side with bent limbs and oriented towards the West, together with some items of horse equipment.

- **Russia, Divnogor'ye 9, 50.963242 N, 39.29496 E:**

Divnogor’ye 9 (or Divnogorie 9) is an archaeological site in the southern margin of the Central Russian Upland. The site contains seven layers rich with bones, which are separated by sterile layers with chalk blocks. The dates produced from the layers 5 and 6 with bone concentrations range between 14,500 and 13,000 C14 uncal BP (17,245–17 336 to 16,329–16 827 cal BP) placing them in the Late Glacial period before the Bølling-Allerød interval and the lithic assemblages represent an Epigravettian technocomplex. The recovered fauna was all attributed to horses - including sample Div9_Rus_m15059 - with a rare occurrence of other fauna, making this one of the largest wild horse assemblages from the Palaeolithic period[171,172].

- **Russia, Hyena's Lair, 51.17025 N, 82.967972 E:**

The Hyena's Lair Cave is located in the North-western Altai. The length of this horizontal cave is about 20 meters, the depth of loose sediments is 1-1.5 meters. The cave taphocenosis was formed in the Late Pleistocene due to the nutritional activity of large predators, primarily cave hyenas. In the Pleistocene layers, almost 6.3 thousand bone remains from 30 species of large mammals were found[173,174]. Among the representatives of the megafauna, the remains of horses of two species predominate (45.7%), of which more than 4/5 belong to the Ovodov horse (*Equus ovodovi*). 18.6% of the bones belong to the steppe bison (*Bison priscus*), 8.8% of the bones belong to the argali (*Ovis ammon*) and Siberian ibex (*Capra sibirica*). The percentage of predators is 11.9%, among them the most numerous are the remains of the cave hyena (*Crocuta crocuta spelaea*) - 6.5%. Based on the composition of theriofauna, during the formation of the taphocenosis, moderately arid steppe landscapes dominated in the vicinity of the cave. Bone samples Logx1_Rus_INF and Logx3_Rus_INF were taken from the remains of large caballoid horses (*Equus ferus*) of this cave[173].

- **Russia, Kamenyi Ambar 5, 52.833333 N, 60.366667 E:**

This site is located on the first above-floodplain terrace of the Karagaily-Ayat River in the south of the Chelyabinsk Oblast. The burial ground consists of thirteen burial mounds. Between 1994 and 2003, 4 mounds of Sintashta culture were excavated, including mounds two, four, and eight (the head of the excavation is Epimakhov A. V.), from which the samples reported in this study originate. 17 burial pits were located in mounds two and four. They contained incomplete skeletons of about 100 individuals of different sex and age. The following were found in the graves: numerous clay vessels (made by hand), weapons made of bronze, stone, bone and horn (daggers, spearheads, arrowheads, bow details), tools (knives, adzes, awls, fish hook, harpoon), jewelry (bracelets, beads), copper ore and metallurgical slags, wheel tracks from chariots and cheekpieces. The funeral rite included the sacrifice of domestic animals: cattle, sheep, goats, horses, pigs, dogs. Whole skeletons and parts of skeletons from several dozen animals were found in the graves[175,176]. Domestic horses were buried in the graves of the Kamennyi Ambar 5 burial ground. The remains of the animals lay at the bottom of the grave pits. They were sacrificed during burial. These are the earliest horses that were harnessed to chariots. A total of seven AMS radiocarbon dates of the bones of people from the burial ground were obtained. These dates fit into the interval from 2,040 to 1,730 calibrated years BC (2σ), which coincide with the existence of the Sintashta culture in the region[177]. The artifacts found in the graves are similar to those of the Sintashta culture[178]. The content of ^{13}C and ^{15}N isotopes[179-181], trace elements[182] and DNA from the bones of people from the burials of this burial ground was studied. Analysis of the isotopes ^{13}C and ^{15}N showed that meat was the main part of the diet of the Sintashta culture people[179-181]. A fraction of 95-97% of animal bones in the settlements of the Sintashta culture belong to domestic animals[183]. In the burial grounds of the Sintashta culture, there are many altars with animal bones (whole skeletons and parts of skeletons), of which 99.9% belong to domestic animals[184]. Animal husbandry was an important part of the economy of the Sintashta culture.

- **Russia, Kostenki 15, 51.376 N, 39.071 E:**

The horse specimen labelled KB212_Rus_m29656 was excavated at Kostenki 15, a locality belonging to the Upper Palaeolithic site cluster discovered in the modern village of Kostenki. A single Early Upper Palaeolithic archaeological horizon was excavated in 1952[185], containing abundant remains of at least eleven butchered horses, including male and female adults, as well as younger individuals, thought to reflect one or more mass kill events at the site[186]. Lithic debitage and tools were also found and have been assigned to the Gorodtsov Culture, as well as the remains of a partially articulated human child burial that was interred alongside a range of grave goods[185-187].

- **Russia, Krasnosamarskoe, 51.497056 N, 85.9745 E:**

Krasnosamarskoe is a Late Bronze Age settlement, located in Samara Oblast Russia, and dated to 1800-1700 years cal. BCE. It is associated with the Srubnaya Culture, and some horse bones, including horse specimens labelled LOR18x43_Rus_m1798 and LOR18x44_Rus_m1109, were found in the cultural layer of the site[188].

Russia, Kuyum, 51.497292 N, 85.971322 E:

Kuyum is a multi-layered and multi-time archaeological site, consisting of 26 graves, located on the right bank of the Katun River at the mouth of the Kuyum tributary, between the villages of Uznezya and Elekmonar (Altai Republic, Russia)[189]. It encompasses several time periods, from the Mesolithic, Eneolithic - associated with the Afanasievo Culture - and up until the Early Iron Age. The horse specimen labelled Kuymx2_Rus_m580 was excavated from mound 9 and is associated with the Iron Age Biyke Culture[190].

- **Russia, Medvezhiya cave, 62.083 N, 58.083 E:**

Medvezhiya Cave is located in a rock shelter near the upper course of the River Pechora, inside the modern permafrost zone in the Ural Mountains, Russia. A sequence of deposits ranging from 2.5 to more than 6 meters depth has been recorded during two main phases of excavations just outside the cave entrance[191]. One archaeological horizon is known, dated by three radiocarbon samples to between 16,100-18,700 ^{14}C years BP. The samples KB217_Rus_m34198, KB218_Rus_m42475, KB219_Rus_m24209 and KB221_Rus_m34198 reported in this study are derived from palaeontological layers (noted as spit contexts) without any evidence of human activity.

- **Russia, Nikolskaya, 55.466667 N, 59.45 E:**

The Nikolskaya cave is located on the western slope of the southern Urals, Russia, dating back to the late Pleistocene. Excavations were carried out in 1997 by Pavel A. Kosintsev. The excavations revealed three layers. In layers 1 and 2, a few bones of the Holocene time were found, while in layer 3, more than 1,000 bones of large mammals were recovered. These include remains of wolves, red foxes, polar foxes, cave lions, wooly mammoths, wooly rhinoceroses, red deer, reindeer, elks, bison, saiga antelopes and horses, among which specimens here referred to as RN128_Rus_m43000, RN129_Rus_m36541 and RN130_Rus_m21145[192].

- **Russia, Noviye Kluchi III, 53.338939 N, 51.870031 E:**

Koviye Kluchi III is a Kurgan cemetery associated with the Pokrovka Culture, and dating back to 2,000-1,700 cal BCE. Two sacrificed domestic horses were found near burial graves, including sample LOR18x4_Rus_m1844[193].

- **Russia, Novoilinka-III and Novoilinka-VI, 53.546361 N, 79.350056 E:**

Samples Nov3x10_Rus_m3443 and Nov3x7_Rus_m345 were excavated from Novoilinka-III, an Eneolithic site located in the Khabarsky district of Altai Krai, Russia. Since its discovery, excavations have revealed about 600 square meters of settlements, with a representative collection of ceramics and stone artifacts from the Eneolithic period. In total, more than 420 bone remains were found on site, species affiliation of the majority of which (approximately ~ 63.7%) has not been determined. The vast majority of the bones, species affiliation of which has been determined, belongs to horses, with almost all skeletal elements being represented. Morphological comparisons between Novoilinka-III and Botai bones revealed very strong morphological similarities[194].

Novoilinka-VI was discovered in 2013, 0.3 kilometers to the West of the Novoilinka-III site, in Altai Krai, Russia. A total of 96 square meters of the settlement have been excavated and investigated,

including dwelling No. 1, from which samples labelled Nov6x6_Rus_m3224, Nov6x7_Rus_m3217 and Nov6x11_Rus_m3217 were excavated. Three cultural horizons have been identified from this dwelling: a first layer associated with the final Eneolithic, when the dwelling was used for waste disposal, a second layer corresponding to the period of functioning of the dwelling from the early Eneolithic, and a third layer dating back to the Neolithic period[195,196]. All three horses included in this study belong to layer 1.

- **Russia, Oroshaemoe I, 50.157528 N, 48.527694 E:**

The Oroshaemoe site belongs to the Orlovka Culture and located by the Bolshoi Uzen' river, Russia, only 250 meters away from Algay. The faunal record at oroshaemoe includes both domestic sheep and various wild animals. The horse sample labelled RN96_Rus_m4586 that is included in this study most likely represents a wild individual, as horses were hunted in that region up until the Russian Empire in the 18th century CE.

- **Russia, Ouren, 53.44725 N, 48.942667 E:**

The horse specimens labelled LOR18x23_Rus_m1796 and LOR18x25_Rus_m1785 were excavated from the Ouren mound, which dates back to the late Bronze Age, and is associated with the Pokrov Culture.

- **Russia, Pershinskaya, 57.45 N, 61.45 E:**

The Pershinskaya I cave is located on the eastern slope of the Middle Urals, Russia. Stratigraphic analyses of sediments revealed four distinct layers, with bones of Holocene mammals and birds in layers 1 and 2, and over 1,000 bones of late Pleistocene large mammals in layer 4, including wolves, foxes, cave lions, wooly mammoths and rhinoceros, horses, muskoxen and deer. Of these, 266 bones belong to horses[197], including the six horse specimens included in this study.

- **Russia, Potapovka, 53.658 N, 50.668444 E:**

Potapovka is a Late Bronze Age site dated to the third mill BCE, and located by the Sok river, Samara region, Russia. In burial 4 of kurgan 3, a man and a child have been found together with weapons and two sacrificed domestic horses. Samples RN01_Rus_m1862 and DaAn01_Rus_m1883 represent some of the earliest horses harnessed to a chariot[198].

- **Russia, Repin khutor, 49.215583 N, 43.904556 E:**

Repin khutor is an ancient settlement, located on top of a hill, on the right bank of the Don River, Russia, and dated to 3,400-2,800 cal. BCE. It is associated with the eponymous Repin Culture, synchronous to the early Yamnaya Culture from the Volga-Ural region, and that some archaeologists even consider an early stage of the Yamnaya Culture. In the cultural layer, over 85% of the animal bones belong to horses, including the horse specimens labelled LOR18x09_Rus_m3089 and LOR18x12_Rus_m2995. It remains unclear whether these horses were wild or domestic[199].

- **Russia, Serpievskaya, 54.833333 N, 57.883333 E:**

Serpievskaya I is a late Pleistocene cave located on the western slope of the southern Urals, Russia. Stratigraphic analyses revealed three layers. Bones of large and small mammals of the Holocene were found in layers 1. In layer 2, more than 135 bones of large mammals were found, while in layer 3, only a few bones were found[192]. The horse remain RN131_Rus_m17769 included in this study was taken from a bone excavated from layer 2.

- **Russia, Sholma 1, 55.883333 N, 47.491667 E:**

The settlement of Sholma-I is located on the first above-floodplain terrace of the Maly Tsivil River of the Chuvash Republic. An analysis of the different types of sites of the Mari-Chuvash Volga region (long-term and seasonal) led to the conclusion that the site represented a peculiar Mesolithic culture at the end of the Boreal - the beginning of the Atlantic periods (late eighth - early seventh mill BCE). The horse bone sample labelled Sho1x2_Rus_m7500 was excavated at settlement Sholma-I and dates back to the 8th millennium BCE. The excavation area was 36 square meters. The settlement has two cultural layers that occur in situ in two humus horizons of the buried soil. More than 15 thousand stone artifacts of flint and quartzite have been found. The fauna includes more than 150 bones of the wild horse (*Equus ferus*), 4 hare (*Lepus timidus*) bones and Eulipotyphla tooth (Soricidae gen.). C14 dates were obtained from two horse bones from the 2007 excavation: 8160 ± 120 BP (Ki-15155) and 9200 ± 200 BP (Ki-15156). According to the leaders of the excavations, the lower buried soil was formed in the Bølling interstadial, and the upper one was formed in the Allerød oscillation. The settlement belongs to the Ust-Kama archaeological culture[200,201].

- **Russia, Sintashta, 52.486359 N, 60.187925 E:**

The Sintashta site is located in Chelyabinsk Oblast, Russia, and is associated with the eponymous Bronze Age Sintashta Culture. It includes a fortified settlement and a large number of graves, including grave 19, from which a chariot and the horse specimens labelled NB44_Rus_m1856 and NB45_Rus_m1865 were excavated[7].

- **Russia, Sosnovka, 51.566667 N, 45.783333 E:**

The settlement is located on the floodplain terrace of the Volga River, Russia, where an area of 120 square meters was unearthed in 1991[202]. Excavations of the cultural layer revealed that almost all archaeological material belongs to the catacomb Culture of the Middle Bronze Age. In the upper horizons of the cultural layer, single fragments of vessels of the late middle (Poltava Culture) and late (Khvalynsky Culture) Bronze Age were found. Among the bone remains, a total of 1,334 bones of cattle, as well as 750 bones of small cattle, 121 horse bones, 23 pig bones, 9 dog bones and 22 bones of wild species were identified[203]. All five horse samples included in this study were taken from the lower horizons of the cultural layer and belong to the Catacomb Culture.

- **Russia, Taymyr, 73.046 N, 109.708 E:**

Two samples, CGG10022_Rus_m40610 and CGG10023_Rus_m14170, have been excavated from an Upper Palaeolithic site in the Taymyr Peninsula, located in North East Siberia. These two individuals correspond to wild individuals. These specimens were previously analyzed at the genetic level by Schubert and colleagues[14].

- **Russia, Turganik, 52.80375 N, 53.793639 E:**

Turganik is an Eneolithic and Bronze Age settlement, dated to 4,900 – 3,300 cal. BCE and located by the Turganik river, in Orenburg Oblast, Russia. Two distinct phases have been identified at Turganik, associated with the Samara Culture and an early form of the Pit Grave (Yamnaya) horizon, respectively. The cultural affiliation of the horse bones found on site could not be determined, which makes it impossible to ascertain whether these were domestic or wild animals[204]. Out of the five horses included in this study, four were radiocarbon dated to approximately ~2,800 cal. BCE, which would fit with the contemporaneous Yamnaya Culture. The fifth sample LOR18x19_Rus_m5351 was dated to approximately ~5,400 cal. BCE, and could potentially be related to the early Eneolithic Samara Culture.

- **Russia, Ullu, 43.79028 N, 42.7278 E:**

The small cemetery of Ullu is located on the mountain plateaus South of the mineral Spa Kislovdsk in the North Caucasus, Russia. Originally thought to be a Bronze Age site, the excavations revealed graves of a much later epoch. Ten excavated interments and three ritual complexes revealed objects that date to the late Scythian period (fifth century BCE). The graves are composed of stone boxes built with slabs or dry walls and clustered into four nearby areas[205]. In two of the graves horse skeletons sacrificed as grave offers were documented. In grave 3, four horses, including the horse specimen labelled BZNK169x1_Rus_m871, each harnessed with bridle and headgear were buried beside an interlinked cluster of four burials. They were densely packed into a stone box of c. 1.6x1 meters and outfitted with complete headgears. These headgears included head and cheek pieces as well as turrets in different typological sets[206].

- **Russia, Utevka VI, 52.918694 N, 50.962611 E:**

The horse specimens labelled RN02_Rus_m1825, RN03_Rus_m1851 and RN04_Rus_m1789 were excavated from Utevka VI, a late Bronze Age site, located in the vicinity of Samara, Samara region, Russia. This site is associated with the Potapov Culture, and has been dated to 2,000-1,900 cal. BCE. Horses found on site represent domestic animals, probably harnessed to a chariot and sacrificed during burial[198].

- **Russia, Uvarovka II, 53.116583 N, 48.351833 E:**

Uvarovka II is a late Bronze Age archaeological site located on the right bank of the Volga river, Samara region, Russia. Eleven mounds were found, located one after another in a chain. In 1996, the easternmost mound 1 was excavated[207]. To the South of the main burial of this mound, the burial of two horses was studied. The horses, including the sample labelled RN09_Rus_m1851, were laid on their sides, with their feet next to each other. The burial belongs to the Pokrov Culture of the first phase of the late Bronze Age[208].

- **Russia, Varfolomeevka, 50.133333 N, 48.05 E:**

Varfolomeevka /Varfolomeev is a dwelling site associated with the Orlovka Neolithic Culture in the steppe region of Volga River, located between the Maly and Bolshoy Uzem Rivers, Russia. Cultural layers of the site reach 2.2 meters and are divided into four chronological layers. The lower level (layer 3) was attributed to the middle Neolithic; the middle layers (2B and 2A) were attributed to the late Neolithic, and an upper layer was attributed to the early Eneolithic. The horse specimens labelled Var2_Rus_m5544 and Var3_Rus_m5549 come from layer 2B, dated to 6,850 ± 40 years BP from the crust on the pottery. Interestingly, besides wild horses and wild asses, aurochs, saigas, red deer, dogs, fish and birds are also represented[209,210].

- **Russia, Verkhnegubakhinskaya, 58.883333 N, 57.633333 E:**

Verkhnegubakhinskaya is a late Pleistocene cave located on the western slope of the middle Urals, Russia. Three distinct layers have been identified by excavations in 2000 and 2001. In layer 2, over 3,000 bones of large mammals were found, belonging to wolves, foxes, cave lions, woolly mammoths and rhinoceroses, horses, deer, bison and muskoxen. Of these, 788 bones belonged to horses, including the remains here referred to as RN120_Rus_m30192 and RN121_Rus_m21420[211].

- **Russia, Yana, 70.723611 N, 135.413056 E:**

The Pleistocene horse bone remains labelled R17x2_Rus_INF, R17x3_Rus_INF, R17x5_Rus_INF and R17x6_Rus_m28567 have been collected from the river bank exposure in the low reaches of the Yana river, in the western part of the Yana-Indighirka lowland, Russia. This exposure is located on 195

kilometers of the Yana river fairway, on its left river bank. All deposits within the study area at Yana are permafrost deposits, except for seasonally-melted portions observed in the profiles. The faunal record at Yana consists of thousands of the Pleistocene animal bones and bone fragments[212-214].

- **Russia, Yukagir, 72.69086 N, 142.821769 E:**

The "*Yukagir horse*", here referred to as Rus30x31_Rus_m3435, was discovered next to the Dmitry Laptev Strait, in northern Yakutia, Russia, in 2010[215]. The horse was a rather small 5-year old female adult which lived approximately ~4600 years ago, whose body got frozen and naturally mummified, and hence particularly well preserved. Morphological analyses of the remains revealed that the specimen probably belonged to the horse (sub)species *Equus lenensis*[216].

- **Slovakia, Nitriansky Hrádok, 48.069502 N, 18.200384 E:**

The horse specimen labelled NHr2_Slo_m1683 originates from Nitriansky Hrádok, a fortified settlement near Šurany, Slovakia. It dates back to the Early and Middle Bronze Age and is associated with the Mad'arovce Culture (2,200-1,600 cal. BCE). The faunal assemblage at Nitriansky Hrádok is dominated by domestic animals - and especially cattle - which represent 85% of the assemblage.

- **Spain, Cantorella, 41.552778 N, 1.039444 E:**

Cantorella is a settlement located in the Corb valley, Catalonia, Spain, with two distinct human occupations in the Final Neolithic-Eneolithic and in the Bronze Age, respectively. The horse specimen labelled UE2275x2_Spa_m2758 was unearthed from a silo dating back to the Final Neolithic-Eneolithic period, which contained two horse skulls. Horses from this first occupation dominate the faunal assemblage and likely represent an autochthonous population of horses[217]. This specimen was previously analyzed at the genetic level by Fages and colleagues[5].

- **Spain, Cova Fosca, 40.42019 N, -0.12547 E:**

The Cova Fosca site is a karst cavity located in the Alto Maestrazgo region, province of Castellón, Comunidad Valenciana, Spain[218]. In Cova Fosca, uninterrupted human occupations have been documented from the Mesolithic and throughout Neolithic, with sporadic occupations in the Epipalaeolithic[219]. Specifically, recent investigations focusing on the stratigraphic sequence of one specific sector of the site have enabled the identification of several layers, including surface levels, a middle Neolithic level, an early Neolithic level, a Mesolithic level and an Epipalaeolithic level[220,221]. The specimen CFo02_Spa_m5180 was sampled from a fragment of a right maxilla recovered from the early Neolithic level[222]. The sample was identified as a wild horse, *Equus ferus*, based on the chronology. Other animal taxa detected in Cova Fosca include domestic dogs, sheep, as well as birds, reptiles, fishes and molluscs. Altogether, this represents the richest faunal assemblage thus far retrieved in the eastern Iberian Peninsula[223,224].

- **Spain, El Acequión, 39.024444 N, -2.028056 E:**

El Acequión is a Bronze Age village, situated in the vicinity of Albacete, Spain. A great number of horses, including the samples labelled Spain38_Spa_m2040 and Spain39_Spa_m1968, were found on site. As many of the excavated horse bones display marks of human activity, archaeologists have suggested that at least some of the horses could be domestic animals[225]. These specimens were previously analyzed at the genetic level by Fages and colleagues[5].

- **Spain, El Turuñuelo, 38.949334 N, -6.064916 E:**

The horse specimen labelled 18ELTu18_Spa_m588 was recovered in the yard of the Casas del Turuñuelo site (Guareña, Badajoz, Spain), an archaeological site associated with the Iron Age Iberian

Culture of Tartessos[226]. In 2017, a lower level and a yard were discovered, revealing a large number of animals intentionally sacrificed and deposited, currently including 40 equids, five cows, four pigs and a dog. As such, Turuñuelo represents the only site in the western Mediterranean region that has provided combined material evidence for an offering of animals, ceramics, textiles and cereals[226,227].

- **Spain, Els Vilars, 41.57 N, 0.95 E:**

The fortress of Els Vilars, located in the Segre valley, Catalonia, Spain, is a complex defensive site, occupied between 750 BCE and 325 BCE. The fortress has been built and developed throughout four distinct periods: Vilars 0, I, II and III[228]. Intriguingly, people inhabiting Els Vilars started burying horse foetuses by Vilars I, an unprecedented ritual practice that seemed to have become more common throughout Vilars II. The foetuses, 4 to 9 months old, show no sign of butchery or chop marks, and were buried in foetal position close to near entrances, with their back against the wall[229]. The horse specimens labelled UE11080x2_Spa_m664 and Fetusx9m_Spa_m475 represent such foetuses from Vilars I and Vilars II, respectively, while the horse specimen labelled UE4618_Spa_m655 was sampled from an adult bone in a simple domestic unit associated with Vilars I and the horse labelled UE13045_Spa_m738 was sampled from a phalanx, excavated from Vilars 0.

- **Spain, Sigarra, 41.7075 N, 1.54222 E:**

Sigarra (or Sikara, Sikarra, and Segarra) is an archaeological site in the commune of Els Prats de Rei, Catalonia, Spain, that was first occupied by the 6th century BCE. Excavated remains and structures have been attributed to different time periods, from the ancient Iberian phase up until the early Modern period. The ancient Iberian town was located at a strategic position, 600 to 800 meters above sea level, at the crossroads of transhumance passages, thus connecting the Pyrenees to coastal plains of Barcelona and Tarragona. By the 3rd century BCE, it became a major Roman economic and political center of Catalonia[230]. The horse specimen labelled UE172_Spa_m317 was found in an earth-filled moat dating back to the fourth century BCE with other waste from food consumption.

- **Tunisia, Althiburos, 35.873429 N, 8.78621 E:**

Althiburos is an archaeological site located in the northern part of the Ksour massif, in North-West Tunisia. Excavations at Althiburos revealed an ancient Roman city as well as pre-Roman occupations[231,232], from the 10th century BCE until the fifth century CE. The three horse specimens included in this study, SV19x18_Tun_m581, SV19x19_Tun_m643 and SV19x22_Tun_m660, all date back to the sixth and fifth centuries BCE, corresponding to the so-called 'middle numidian' period. The faunal assemblage during this time period is dominated by remains of cattle, goat, sheep, and pig - most of which representing waste from food consumption - while equid remains are very scarce[233]. The presence of cut and chop marks, together with burnings in some anatomical elements, suggest occasional consumption of equid meat in the Numidian period.

- **Turkey, Acemhöyük, 38.411227 N, 33.835694 E:**

Acemhöyük is a large mound site located in the Aksaray province of central Turkey representing an important urban center in the Early and Middle Bronze Age (~2,800-1,700 BCE)[234]. The site is located at an elevation of 950 m above sea level on the alluvial fan of the Melendiz river near the South-western tip of the central Anatolian Great Salt Lake (Tüz Gölü). Acemhöyük consists of twelve levels with deposits representing Bronze Age (contemporaneous with the Eneolithic) to Medieval occupations. The horse specimen labelled AC7970_Tur_m290 is an astragalus identified based on morphology and its large size as a horse. It was excavated in 2009 from excavation area DB/48-49 and was initially associated with Bronze Age level IV. However, a direct date places it chronologically in the early 3rd century BCE and associated with a Hellenistic period village located on the top of the Bronze Age mound. This specimen likely represents a domestic horse. The horse specimens labelled

AC8811_Tur_m2125 and AC9016_Tur_m1900, sampled from mostly complete phalanges, were both identified as horses due to their large size. AC8811 was excavated in 2010 from a trash pit in area DB/48 associated with level IV and is directly dated to 2,200-2,040 BCE representing the end of the Early Bronze Age. Specimen AC9016 derives from the same excavation unit (also from work done in 2010) and is dated slightly later, 1970-1870 BCE, based on an associated date (Beta-305371: 3560 ± 30 uncal. BP) from animal bone recovered from the same locus. Based on context and date, it is likely that these two specimens represent domestic horses but since wild horses are known from the Anatolian plateau until at least the 4th millennium BCE, the status of these animals is not clear based on the archaeology alone.

- **Turkey, Çadır Höyük, 39.676776 N, 35.1436 E:**

Çadır Höyük is a modest sized mound site with lower terrace located in the Kanak Su basin situated in the Yozgat Province in central Turkey. Excavation and survey work have defined an occupation sequence extending from the Early Chalcolithic (contemporary with the Eneolithic) (late sixth mill BCE) through to the Byzantine period (ending ~1,000 CE)[235-237]. The site displays evidence for major Hittite period terrace walls suggesting a small but significant occupation of the mound during the Late Bronze Age. A long sequence of deposits dating to the Iron Age indicate that the mound was a site of industrial activity during this period as well. The horse specimen labelled CD1819_Tur_m1299 represents a complete radial carpal identified as domestic horse dating back to the Late Bronze Age (Hittite Empire). The horse specimen labelled CD2017_Tur_m4532 represents the proximal radius of a horse excavated from trench LSS5, a well-sealed deposit situated in a deep sounding dated to the Middle Chalcolithic (contemporary with the Eurasian Eneolithic) and is dated to the mid fifth mill BCE (4,610-4,460 BCE). It likely represents a local Anatolian wild horse lineage, yet it is impossible to say whether this horse was hunted as a free-living animal or if it was managed under human care. The horse specimen labelled CD5203_Tur_m985 was sampled from a left side maxillary tooth. The occlusal surface of this tooth is remarkably flat suggesting atypical tooth wear likely reflecting management and foddering. This specimen was excavated from trench USS4, and a direct date places it in the Iron Age (1,050-920 BCE). Based on the age of the specimen it is assumed to represent a domestic horse.

- **Turkey, Kırklareli-Kanlıgeçit, 41.721357 N, 27.225306 E:**

Kırklareli-Kanlıgeçit is an Early Bronze Age settlement mound located in Thrace Turkey, dating back to ~2600-2400 cal. BCE. Excavations have revealed a large faunal assemblage, consisting mainly of remains of domestic animals, including cattle, sheep/goat, pig and horse, including the horse specimen labelled Kan22_Tur_m2386, while wild animals only represent 8% of the animal fossil record. Morphometric analyses on animal bones indicate that horses at Kırklareli-Kanlıgeçit were strong and resilient animals, likely used for riding and/or as labour work force[238,239].

- **Turkey, Köşk Höyük, 37.847803 N, 34.611841 E:**

Köşk Höyük represents the remains of a small farming settlement located on the eastern margin of the broad Ereğli-Bor Plain, Niğde, Central Turkey. Köşk Höyük provides a rare glimpse into lifeways in central Anatolia during the transition from the Late Pottery Neolithic into the Early Eneolithic in central Anatolia[240-242]. The subsistence economy at Köşk Höyük was based on agriculture and the herding of domestic livestock including primarily sheep and goats with secondarily domestic cattle[242]. Additionally, large game was also an important component of the Köşk animal economy as the remains of large numbers of wild equids and also aurochs have been recovered from pit features interpreted as roasting pits, including specimen KSK16232_Tur_m6319, which represents a long bone fragment, unearthed from a large pit feature dug into bedrock and from which many equid remains were recovered. Given the age of the deposits, the general lack of archaeological evidence for management, and archaeozoological evidence for a long tradition of horse hunting

extending back into the early Holocene in the region, it is assumed that this horse represents a wild individual from a population of wild horses, isolated from other Eurasian horse populations.

- **Ukraine, Semenovka 1, 46.86888889 N, 35.43305556 E:**

The Semenovka 1 site is located in the outskirts of Melitopol, Zaporozh'e Region, southern Ukraine. This multilayer site is situated on the first terrace of the Molochnaja River right bank and includes layers from the Neolithic up until the Middle Ages[243]. Only the Mesolithic, Neolithic and Eneolithic layers with thickness from 1.5 to 2 meters were preserved to the moment of excavation, but their upper horizons were partially mixed on some areas. All layers have radiocarbon dates[244,245]. The horse specimen labelled Ukr11_Ukr_m4185 included in this study is associated with food disposal and was found in the Eneolithic layer in excavation area 1. This area contains materials from the Dereivka Culture (4,300-3,700 cal. BCE).

- **United Kingdom, Chalk Hill, 51.330592 N, 1.389657 E:**

The horse specimens labelled Chalk1_UK_m865 and Chalk3_UK_m474 were recovered from Chalk Hill, Kent, UK by Canterbury Archaeological Trust. This multi-period rural site is located on the southern coast of what is now the Isle of Thanet, South-East England[246]. Both specimens come from a linear hollow stratigraphically above a Late Bronze Age (~1,100-800 BCE) sub rectangular enclosure. Interpreted as a medieval hollow way, this feature also contains material eroded from earlier features including 49 sherds of Late Bronze Age pottery and one sherd of Late Iron Age pottery from context 409. Radiocarbon analyses of the two horse remains indicate one dates to the Late Bronze Age (Chalk1_UK_m865) and the other to the Early Iron Age (Chalk3_UK_m474). Based on the context and dates of the finds, these two individuals are interpreted as domestic horses.

- **United Kingdom, Kents cavern, 50.467709 N, -3.502667 E:**

Kent's Cavern is a cave system in Torquay, Devon, UK. The cave has stratified archaeological and palaeontological deposits dating from the middle Pleistocene to the Holocene. It is famous for having produced the earliest directly dated specimen of anatomically modern human in North-West Europe, a fragment of maxilla radiocarbon dated to 44.2–41.5 kyr cal BP[247]. The horse specimen labelled P9392_UK_m12666 was sampled from a horse molar excavated in 1867. This particular tooth has been directly dated within the Pleistocene, with a radiocarbon age of 12,500 ± 60 BP (Oxa 17545)[248].

- **United Kingdom, Magor, 51.578275 N, -2.829228 E:**

The horse specimen labelled Magor_UK_m1009 was recovered from a skull in a limited archaeological excavation at Magor by Archaeology Wales, near Caldicot, Wales. The find was unearthed at the base of an oval pit filled by a light fibrous peat, and containing no other finds. It represents a complete cranium of an elderly male horse, aged between 14 and 20 years at death, with no associated mandible, and no evidence of butchery. The animal is interpreted as domestic based on the date, context and comparable treatment of similar British Late Bronze Age finds.

Museum specimens

Our final dataset includes the genomes of three museum specimens, two of which were previously characterized[61]. These two specimens (Holotype_Mon_1870 and Paratype_Mon_1899) belong to the Przewalski's horse lineage and date back to the 19th century CE. The DNA of an additional museum specimen was analyzed here, for the first time. This pertains to Tarpan_Ukr_1868, sampled from a fragment of rib of the skeleton of a tarpan that lived in the Rakhmanovskaya steppes of the Kherson Region and died in 1868 CE. The cranial and post-cranial skeleton are preserved in the collection of the Zoological Institute (ZIN) Russian Academy of Science (Sankt-Petersburg). Collection number is № O.521. This specimen is often referred to as the "*Shatilov*" tarpan.

Radiocarbon dating

A total of 207 radiocarbon dates have been included in this study, the vast majority of which (179/207) are new. All novel radiocarbon dates were carried out at the Keck Carbon Cycle Accelerator Mass Spectrometry Laboratory, UC Irvine. For each sample, approximately ~1g of material was sampled in ancient DNA facilities at CAGT, Toulouse (France) and sent for dating of ultra-filtered collagen. Subsequently, calibrations were performed using OxCalOnline[49] based on the IntCal20 calibration curve[48] (SI Table 1).

Supplementary Methods 2. Genome sequencing

Ancient DNA was extracted in the ancient DNA facilities of the Centre for Anthropobiology and Genomics of Toulouse (CAGT), University Paul Sabatier, France. The overall methodology follows that presented in previous work, especially the work from Fages and colleagues[5]. Briefly, osseous remains were drilled into powder using either the Maxima PHP35 (Argofile) instrument and subsequently the Mixel Mill MM200 (Retsch) Micro-dismembrator, for 30 s -2 mins at 25 Hz. Osseous remains consisted mostly of petrosal bones and teeth, as petrosal bones are generally associated with better DNA preservation rates[249]. A total of 100-590 milligrams of powder were then extracted for ancient DNA using the procedure from Yang and colleagues[250], as modified by Gamba and colleagues[251]. This procedure includes a first digestion step for 60 mins at 37°C, and is aimed at removing DNA contaminants located at the powder surface. It is followed by a second digestion step, in which the remaining undigested pellets are collected following a rapid centrifugation at 2,000 rpm for 1 min and digested at 42°C overnight within 4 mL of extraction buffer (0.45 M EDTA, 0.25 mg/mL proteinase K and 0,5% N-lauryl Sarcosyl). DNA is then concentrated into approximately 200-250 uL through Millipore Amicon-30kD columns, before being further purified using MinElute QIAgen columns following the manufacturer's instructions, except that the last elution step is carried out twice using EB solution that has been pre-heated at 37°C for 10 mins and supplemented Tween (final concentration = 0.05%).

Ancient DNA extracts were subjected to mild USER-treatment following the conditions from Fages and colleagues[5], and using 23 uL of DNA extract and 7 uL of USER enzymatic mix (NEB®). Double-stranded and triple-indexed DNA libraries were constructed using the methodology from Fages and colleagues[5], modified from Rohland and colleagues[13], and 14.9 uL of USER-treated DNA extracts. In this procedure, the ends of ancient DNA templates are first repaired using T4 DNA polymerase and T4 DNA polynucleotide kinase (NEB E6050, 1.25U in 25uL final reaction volume) 20 mins at 12°C and then 15 minutes at 37°C. End-repaired DNA templates are then purified using MinElute columns as described above, and further ligated to DNA adapters consisting of the Illumina DNA sequencing primer followed by a unique 7-bp index. As such indexes are present on both adapters, the first 7-bp of each sequencing read will provide the sequence of the library index so as to allow library read demultiplexing post-sequencing. After adapter ligation, libraries are filled-in using the Bst polymerase (NEB, final concentration = 0.462U/uL) for 20 mins at 37°C and then inactivated for 20 mins at 80°C. Unpurified DNA libraries were amplified for 10-15 PCR cycles using the InPE1.0 primer and a custom forward oligonucleotide (5'-AAT GAT ACG GCG ACC ACC GAG ATC TAC ACT CTT TCC CTA CAC GAC GCT CTT CCG ATC T-3' and 5'-CAA GCA GAA GAC GGC ATA CGA GAT XXX XXX GTG ACT GGA GTT CAG ACG TGT-3', respectively). The latter includes a 6-bp index ('XXX XXX' in oligonucleotide sequence) that can serve as a third read index for demultiplexing. Amplification reactions were carried out in 25 uL reaction volumes using 1 unit of AccuPrimeTM Pfx DNA polymerase, 4-6 uL of DNA library and with an overall concentration of 200 nM of each primer, including the InPE1.0 primer and one custom PCR primer. Amplified DNA libraries were purified using Agencourt Ampure XP beads (beads-to-library volume ratio = 1.4:1) and eluted in 220 uL EB+0.05% tween. Purified DNA libraries were quantified using the TapeStation (Agilent) instrument and the D1000 chip, as well as the QuBit (Invitrogen) instrument and the high-sensitivity HS dsDNA assay. DNA libraries were then pooled at equimolar concentrations and shotgun sequenced on the Illumina MiniSeq instrument (paired-end mode, 2x75) so as to assess the library endogenous DNA content and clonality following PALEOMIX mapping (bam_pipeline version 1.2.13.2) against the horse reference genome EquCab3[53], appended with the Y-chromosome contigs from[55], and the mitochondrial reference genome (NC_001640[54]).

Raw fastQ files were demultiplexed using AdapterRemoval v2[52], allowing a maximum of one mismatch in each 7-bp internal index. Reads showing low quality ends and/or adapter sequences (--trimns --trimqualities --minadapteroverlap 3 --mm 5 --minlength 25) were also trimmed during demultiplexing, while those showing sufficient sequence overlap were collapsed (--collapse). PALEOMIX was then used with the parameters from[56] and Bowtie2 (local mode), filtering BAM alignments realigned locally around indels for PCR duplicates and for reads showing mapping quality scores inferior to 25. mapDamage2[57] (version 2.0.8) nucleotide mis-incorporation and DNA

fragmentation profiles were generated automatically through PALEOMIX from a total of 100,000 random reads per DNA library. These exhibited all the features from ancient DNA templates that have been subject to USER-treatment, including: (1) an over-representation of Cytosine (Guanine) residues at the genomic position located one nucleotide prior (after) sequencing starts (ends), and; (2) the persistence of faint C→T (G→A) nucleotide mis-incorporation rates towards read starts (ends). Samples for which DNA libraries showed endogenous DNA content compatible with an economical characterization of the horse genome by shotgun sequencing were selected and additional DNA libraries were produced before proceeding with further sequencing on the Illumina HiSeq4000 instruments from Genoscope (paired-end mode, 2x76; France Génomique). This is true for all the samples investigated in this study, except for four (BPTDG1_Fra_m11800, Closeau3_Fra_m10400, Novoil1_Kaz_m1832 and Novoil2_Kaz_m1832), which were sequenced at Novogene Europe on an Illumina NovaSeq instrument (S4 lanes, paired-end 2x150 mode). Our procedure allowed us to obtain a total of 264 novel ancient horse genomes at 0.01-fold to 17.47-fold average depth-of-coverage (median = 1.71-fold; 203 above 1X average depth-of-coverage) from 1,029 DNA libraries (980 new) and 31.86 (30.71 new) billion sequencing read pairs and 100.82 (96.94 new) billion collapsed read pairs (SI Table 1).

Supplementary Methods 3. Genome analyses

Uniparentally inherited markers

Mitochondrial DNA and Y-chromosomal sequence alignments were processed through the following procedures. For mitochondrial DNA, PCR duplicates and sequence alignments showing mapping qualities inferior to 25 were filtered and bases showing individual quality scores lower than 30 were also disregarded. Individual positions along the mitochondrial genome were called using a majority rule and as long as at least five individual high-quality reads were present. A total of 193 haplotypes previously characterized from ancient horses[3,5,14,59,63] were aligned together with the 264 novel haplotypes generated here, using mafft[64] (version 7.407). The resulting mitochondrial alignment was further divided into six sequence partitions, following previous work[3,5], and consisting of the Control Region, all tRNAs, both rRNAs and first, second, and third codon positions. The best substitution model for each partition was identified using ModelGenerator v0.85[68]. Once partitioned, the alignment with 457 ancient samples was first used as input for phylogenetic inference with RAxML 8.2.11[65]. A hundred bootstrap pseudo-replicates were conducted to assess node support. Out of the 457, only 266 radiocarbon-dated ancient specimens were retained for additionally reconstructing demographic trajectories, through Bayesian Skyline Plots. Input files for BEAST (v2.5.1[69]) were prepared in BEAUTi (version 2.5.2[66]), (1) specifying individual sequence dates in years before present, (2) reporting each individual partition substitution model according to the ModelGenerator[68] results, (3) selecting the Bayesian Skyline demographic model[69], and; (4) assuming uncorrelated log-Normal relaxed molecular clocks[252]. BEAST was run for a total of 1,000,000,000 generations. Convergence was checked using Tracer (version 1.7.2[70]). Posterior distributions were obtained using 30% as burn-in.

A more stringent procedure was followed to obtain an alignment of high-quality variants along the Y-chromosome assembly. This was so not only because the Y-chromosomal contigs show lower sequence coverage than mitochondrial and autosomal loci, but also because full-length Y-chromosome assembly is not available for horses. The absence of a full-length assembly for the Y-chromosome implies that some non-optimal read alignments could pass our mapping criteria. For example, paralogs that are not yet present as multiple copies in the available set of Y-chromosomal contigs would result in reads aligning against an apparently unique position. To limit the impact of such situations, we excluded (1) Y-contigs shorter than 5 kb, as usually representing highly-repetitive unassembled regions, or encompassing (2) more than 1% of sites identified as multicopy, or (3) more than one percent of the sites potentially present in non-MSY chromosomal regions. Criteria 2 and 3 were following the estimates reported by Felkel and colleagues[55]. This provided a total of 967,543 high-quality sites allegedly present on the MSY region of the horse Y chromosome. We then applied the strict filtering strategy described below for autosomal data (see section '*Pseudo-haploidizing ancient genomes*'), provided that the MSY-linked site was present in at least 20% of the horses, while considering only specimens with at least 20% of the sites covered. This resulted in a final alignment of 3,195 nucleotide transversions, for 142 specimens, which was further filtered to only consider DOM2 members (N=76). Input files for BEAST (v2.5.1[66,69]) were prepared in BEAUTi (version 2.5.2[66]), (1) specifying individual sequence dates in years before present, (2) selecting the Jukes-Cantor mutational model (as transitions were disregarded), (3) selecting the Bayesian Skyline demographic model[69], and; (4) assuming uncorrelated log-Normal relaxed molecular clocks[252]. BEAST was also run for a total of 1,000,000,000 generations. Convergence was checked using Tracer (version 1.7.2[70]). Posterior distributions were obtained using 30% as burn-in. The resulting Bayesian skylines are shown in Extended Fig 3ef.

An additional Maximum Likelihood phylogenetic tree was constructed based on the same final multifasta sequence alignment, not restricted to DOM2 horses (N=142). The final tree was computed using IQtree (version 1.6.12), following AICc selection of the best substitution model and evaluating node robustness from 1,000 ultrafast bootstrap approximation[71,72].

Error rates, trimming and rescaling procedures

The sequencing error profile of each individual BAM alignment file was assessed using the procedure described by Fages and colleagues[5] and disregarding bases showing quality scores inferior to 20. Here, the genome of a Mongolian horse was selected as the 'perfect genome'

(specimen KB7754[63]), and the ancestral genome was reconstructed from the consensus base shared by at least 5 of 7 outgroups, including 2 donkeys, 3 zebras and 2 Asiatic asses obtained from[59]. This provided average error rates between 0.000337-0.003966 errors per site, and revealed that nucleotide C→T and G→A mis-incorporation rates were still inflated relative to their reciprocal substitution types (T→C and A→G), despite USER treatment. BAM alignment files were, thus, further processed in order to reduce the impact of such nucleotide mis-incorporations in downstream analyses. Here, we used PMDtools (version 0.60[61]) to separate those aligned reads that likely contain post-mortem DNA damage (--threshold 1; DAM) and those that did not (--upperthreshold 1; NODAM). The NODAM fraction of the reads were directly trimmed for 5 bp at their ends where individual base qualities drop (in collapsed reads, bases occupying a more central location are read twice, and their individual quality is, thus, improved). The DAM fraction of the reads was rescaled using mapDamage2[57] version 2.0.8), penalizing all instances of C→T (G→A) as these represented potential derivatives of post-mortem Cytosine deamination. The resulting rescaled alignments were further trimmed for 10 bp at both ends, before being merged again with NODAM trimmed reads. It is noteworthy that trimming only 5 bp in the NODAM fraction, versus 10 bp in the DAM fraction, allowed us to retain a significant fraction of NODAM sites that would have been otherwise bluntly disregarded. Individual genome error profiles were then obtained using the same procedure as above, and provided average error rates lower than those estimated first, ranging between 0.000080-0.000933 errors per site (SI Table 1). These BAM alignment files were used in all downstream analyses.

Pseudo-haploidizing ancient genomes

To prevent biases resulting from uneven and/or low sequencing depths, all BAM files were pseudo-haploidized using a two-step approach. First, the number of reads supporting each nucleotide variant for each position was obtained using ANGSD[60] (version 0.933-86-g3fefdc4, htslib: 1.10.2-106-g9c35744) (-dumpCounts 4), following a series of strict filtering criteria:

- Minimum base quality equal to 30 (-minQ 30)
- Minimum mapping quality of 25 (-minMapQ 25)
- Retain uniquely mapping reads (-uniqueOnly 1)
- Remove reads flagged as bad (-remove_bads 1)
- Remove triallelic sites (-rmTriallelic 1e-4)
- Downscale mapping quality of reads with excessive mismatches (-C 50)
- Keep nucleotide transversions that, accumulated across samples, have a probability of being a SNP equal to 1 (-SNP_pval 0)

The latter filter was critical to exclude sequencing errors, particularly substitution types that could be caused by oxidative damage during library preparation, mostly reflected as C→A and G→T transversions[253] (Extended Fig 1). We additionally excluded SNPs located within genomic regions exhibiting sequencing depths significantly lower or greater than the genome average, possibly caused by Copy Number Variants (*ie*. the 1% and 99% quantiles of the position-wise coverage distribution were excluded). This provided a total of 10,205,277 nucleotide transversions fulfilling all the above requirements from which one high-quality read was randomly sampled as representing that position.

Error rates were assessed using the procedure described above measuring the lack/excess of derived mutations relative to a high-quality genome (ie. from a modern Icelandic horse[89]). They were found negative for all ancient specimens, consistent with the fact that they were obtained from ancient individuals that, thus, lack a significant number of generations (and accumulation of mutations) relative to modern individuals. These rates ranged from -0.0012 to -0.0150 missing derived mutations per site identified as a nucleotide transversion across the whole panel of horses. More importantly, this range of variation significantly and negatively correlated with the age of the sample (Spearman correlation coefficient; r=-0.77; P=0), supporting a minor impact of post-mortem damage after applying our strict filtering strategy. Indeed, post-mortem damage is expected to cause an excess of artifactual derived mutations correlating positively with the age of the samples, not negatively. This highlights the quality and unbiased nature of our panel of nucleotide transversions, which served as input for all subsequent analyses (Extended Fig 1).

Struct-f4*: F*-statistics and admixture proportions

- **Developing struct-f4**

Previous horse palaeogenomic studies have revealed (1) the existence of multiple lineages co-existing at the time of domestication[5,14,15], (2) pervasive gene flow between some but not all lineages[3,5], and (3) the possible introgression from one, and possibly two, ghost populations that have remained unsampled, but were suggested from the presence of extremely divergent uniparental markers in some of the lineages sampled[5].

To further investigate the complex and dynamic structure of horse populations, we developed a new version of the Struct-f4 statistical framework first presented by Fages and colleages[5], aimed at both assessing individual genetic affinities within a panel of genomes and estimating their individual composition of *K* genetic ancestries. Struct-f4 leverages a full set of f_4 statistics and automatically provides individual ancestry coefficients, without requiring pre-defined, ad-hoc set of reference and test populations.

The main difference between Struct-f4 and other available clustering methods, such as ADMIXTURE[77] and Ohana[78], is that Struct-f4 does not assume Hardy-Weinberg equilibrium for delineating the ancestral populations that best partition the samples into *K* groups. This hypothesis indeed comes as an important limitation, which has been thoroughly described elsewhere[79], and results in possible mis-interpretation of drifted samples as ancestral homogeneous groups instead of highly derived mixtures of multiple populations. Struct-f4 offers a solution to this problem by relying on the calculation of the widely-used f_4 statistics, which were originally devised not only to test for admixture, but also to quantify the drift between the internal nodes of a population tree. The latter provides a direct representation of the true ancestral populations. Overall, Struct-f4, thus, implements a more natural and robust (model-free) approach than other clustering alternatives.

As an example to introduce notation, let assume three hypothetical populations, namely *A*, *B* and *C*, and an outgroup *O*. Following Newick standards, assume these are related as *(((A,B),C),O)* (A represents population 1, H1, B population 2, H2, and C population 3, H3). The f_4 permutation in the form $f_4(A,C; B,O) = (p_A - p_C)(p_B - p_O)$ would quantify the drift from the node immediately ancestral to *C*, to the node ancestral to populations *A* and *B*. The Struct-f4 mixture model parametrizes $(p_A - p_C)$ and $(p_B - p_O)$ in terms of: (1) admixture coefficients for each individual (Q), and (2) the drift (*D*) between a given number of potentially ancestral populations (K, defined by the user),

$$(p_A - p_C) = \sum_i^K Q_{Ai} \sum_j^K Q_{Ci} D_{ij}$$

Following the same logic, a similar expression can be written for the second term of $f_4(A,C; B,O)$, namely $(p_B - p_O)$. Therefore, the f_4 statistics $f_4(A,C; B,O)$ can be fully modelled through both matrices *Q* and *D*, as follows:

$$(p_A - p_C)(p_B - p_O) = \left(\sum_i^K Q_{Ai} \sum_j^K Q_{Ci} D_{ij}\right)\left(\sum_i^K Q_{Bi} \sum_j^K Q_{Oi} D_{ij}\right) \qquad (2)$$

Assuming that each f_4 permutation closely follows a Normal distribution, as commonly done, calculating the likelihood of the parameters *Q* and *D* across all *n* f_4 permutations becomes straightforward:

$$lnL = ln\left(\frac{1}{\sqrt[2]{2\pi}}\right) + \sum_{i=1}^{n}\left(\frac{f_{4\_observed\_i} - f_{4_{predicted\_i}}}{s_i}\right)^2 - \sum_{i=1}^{n} ln(s_i)$$

where s_i is the standard error of the observed f_4 permutation *i*, calculated by 5Mb block-jackknifing. The predicted f_4 is derived from the parameters Q and *D*, following equation (2). Struct-f4 maximizes

the likelihood of Q and D by means of an adaptive Metropolis-Hastings MCMC, warranting optimal solutions despite transiting highly rugged likelihood landscapes.

Struct-f4 was implemented in Rcpp, which allows leveraging both the statistical and graphical capabilities of the R programming language, while harnessing the speed of C++ for core numerical calculations. It is available under an Open Source License at https://bitbucket.org/plibradosanz/structf4/src/master/.

- **Validating Struct-f4:**

The accuracy of Struct-f4 was assessed through extensive coalescent simulations using fastsimcoal2[80] (version 2.6.0.3), with mutation and recombination rates of 2.3 x10^{-8} and 1.0x10^{-8} events per generation per site, respectively, and an entire array of population histories, ranging from simple to more complex.

To illustrate the performance of Struct-f4, we only provide here one such example of complex population history. It was selected to match both the evolutionary time frame, the genetic diversity and the number of phylogenetic lineages identified in this study. Let consider an arbitrary tree depicting the relation between 10 populations, evolving under realistic parameters, as summarized in Extended Fig 2a. Ten simulation replicates were run to capture the stochasticity of the evolutionary process. To approximately mimic the horse genome size, we simulated 31 chromosomes of 75Mb each, assuming increasing gene flow (m=0% to 25%, using 5% increments) from population *E* into population *B*. Five individuals were sampled per population, except for the outgroup population, where we only considered one genome. This outgroup genome was fixed in the f_4 permutations as population *O*, to mirror the analysis of the 283 horse specimens and the DONKEY described below. Note that disregarding the remaining f_4 permutations (ie. those with other outgroups than the donkey, such as the $f_4(A,B;C,D)$ configuration) provides a conservative evaluation of the Struct-f4 performance, as solely exploiting a minor subset of all the available information. Yet, fixing the outgroup represents the only practical strategy to analyze the large data set generated in this study, as the number of underlying topologies, hence f4-permutations, increases non-linearly with the number of outgroups (see below).

The underlying ancestry components recovered by Struct-f4 for the five individuals belonging to population *B* and the 41 belonging to the other populations are shown as Extended Fig 2c. Struct-f4 correctly clustered each individual into its ancestral population, as represented by its immediately internal node in the population tree (m=0%). This was successfully achieved despite their corresponding effective sizes (10,000 individuals) largely exceeding most simulated split times. Minor noise was only perceptible for populations descending from internal nodes separated by less than 2,000 generations (*ie*. ~16 ky assuming 8 years per generation). A much finer-scale resolution could be possibly attained by including all f_4 permutations (n=489,555 in total) in addition to those with population *O* fixed as outgroup (42,570). Yet, the simulations reveal that resolution at the level of 2,000 generations or less is excellent, which provided sufficient power to uncover the deep phylogeographic structure in horses, mainly established prior to the Last Glacial Maximum), as validated in the forthcoming sections.

The inferred ancestry proportions were highly significantly and positively correlated with the amount of gene flow used in the simulations (Spearman correlation coefficient = 0.9, p=0). These varied between the 10 simulation replicates, each including 5 individuals from population *B* (Extended Fig 2b). Such variation was not due to Struct-f4 inference, but to the stochasticity of the evolutionary process simulated, given that the $f_4(A,B_i;E,O)$ values - prior to any inference - were already encompassing a large range of Z-scores. This particular $f_4(A,B_i;E,O)$ configuration averaged all f_4 permutations that included individual *i* from population B as H2, and any combination of specimens from populations *A* and *E* as H1 and H3 respectively. Apart from such underlying stochasticity, the mean of the 50 point estimates (five *B* individuals times 10 simulation replicates) provided a slight underestimate of the true admixture fraction, especially for scenarios involving reduced gene flow (*eg*. m=5%). Further inspection revealed that the f_4 statistics calculated from these scenarios did not significantly deviate from zero, indicating that such limited level of admixture yielded limited impact on the BABA and ABBA patterns underlying the f_4 permutations considered.

Combined, this showcases the full potential of Struct-f4 to infer both genomic affinities and ancestry proportions in an unsupervised manner.

- **Applying Struct-f4:**

As Struct-f4 can make use of all f_4 combinations and as the number of ancient genomes present in our panel is exceptionally large, its application entailed serious computational challenges. With 283 samples (the donkeys *E. africanus somaliensis* and *E. asinus* were fixed as an outgroup population[85]), the number of f_4 permutations is indeed equal to 11,212,743 permutations. The calculation of such a high number of configurations was unfeasible with current AdmixTools implementation[17]. We, thus, also implemented our own parallelizable and optimized code for the calculation of f_4-statistics. This code is written in C and is freely available within Struct-f4.

Following the calculations of all 11,212,743 permutations of f_4-statistics, we used Struct-f4 to cluster each individual genome as a mixture of K=4 to K=9 ancestral genetic components (Extended Fig 4). We excluded a total of 856,207 permutations of f_4-statistics during likelihood optimization to limit the impact of technical batch effects. These involved modern genomes as H1 (or H2). As these have been characterized with different sequencing technologies than those used on ancient remains, the underlying f_4 statistics could overestimate the amount of genetic drift shared between two ancient genomes placed as H3 and H2 (or H1). Similarly, f_4 statistics involving the Holotype_Mon_1870, Kent7_Kaz_m1476 and Kent6_Kaz_m1463 were also excluded, as these three genomes showed slightly inflated C→A and G→T error rates, despite the drastic procedure implemented here for filtering variants (Extended Figs 1 and 5f).

The likelihood of the different clustering models stabilized from K=6, and only marginally improved for increasing values of *K* (Bayes Factors < 1.05; Extended Fig 4). Considering that one ancestral component was fixed to represent the donkey outgroup, Struct-f4 supported the presence of five main genetic components best describing the variation present in our extensive set of individual genomes. This closely mirrors the four monophyletic clades revealed by the Neighbor Joining analysis, detailed below and shown in Fig 1b.

We next assessed the difference in likelihood between a model assuming K=6 clusters and a model in which each sample is assumed to represent a distinctive K=284 ancestry component. The likelihood improvement was less than ~5% better than the much simpler K=6 model (Extended Fig 4). We, thus, concluded that K=6 provided a good summary of the genetic structure present in our data.

Multi-Dimensional Scaling (MDS) and Neighbor Joining (NJ) tree

We performed an MDS analysis aimed at summarizing all pairwise affinities based on the calculation of f_4-statistics in a simple two-dimensional space. For this, we applied the *cmdscale* function in R, based on the co-ancestry semi-matrix summarizing all pairwise allele frequency shifts, as derived from Struct-f4. The donkey outgroup was removed before MDS calculations, to maximize resolution within horses. We then added the age of each sample as a third dimension to identify temporal changes in the horse genetic population structure (Fig 3a). This unveiled the presence of several clines of genomic affinities within our dataset. First, Upper Paleolithic (UP) horses from the Urals occupied a central position in the MDS space. They were intermediate between Siberian horses belonging to the *E. lenensis* lineage, and western European horses from southern France and Iberia. Interestingly, UP horses from Central and eastern Europe did not fully overlap with the latter, but were slightly shifted toward a group of horses including Botai and Przewalski's horses. Additionally, genetic affinities were found to extend from the UP horses of the Urals to the Western Eurasian (WE) Steppe. The latter group included horses dated to ~4.7-7.5 kya, forming a geographic cline of genetic ancestry along the WE Steppe. This cline extended from the region of Ukraine and Romania on the one hand, and the Turganik archaeological site (southwestern Russia) on the other hand. Horses from the Central WE Steppe, around the lower Volga-Don region, appeared closest to DOM2. Neolithic Anatolian horses, in turn, were well outside the WE Steppe diversity, and slightly shifted toward Botai and Przewalski's specimens.

We next built a NJ tree to further interpret this geographic and temporal pattern within a phylogenomic context. To achieve this, we first calculated pairwise genetic distances between all samples, using the bed2diffs_v1 program[16]. This software is included within the EEMS package and

was used here instead of other alternatives (*eg*. ngsDist[254]) for consistency with subsequent analyses. The BioNJ algorithm implemented in FastME[73] (version 2.1.4) was then applied to retrieve the tree topology. Node supports were assessed using a total of 100 bootstrap pseudo-replicates. Horses belonging to the *E. lenensis* lineage from Siberia were found to form a well-supported basal clade that was followed by a paraphyletic group of UP horses from the Urals. A group of ancient European horses branched off next, and included East (Hungary, Poland and Romania) and Central (Denmark, Germany, Czechia) European horses in a first subcluster (4-36.7 kya), and West European horses in a second one (Belgium, France, Spain and Portugal; 3.9-36.3 kya-old). Botai and Przewalski's horses clustered together in a different clade, in line with previous work[3]. Finally, the tree included one last monophyletic group of ancient horses from the WE Steppes, basal to all DOM2 individuals, but additionally encompassing Romanian and Anatolian specimens (4.3-8.3 kya).

The phylogenetic placement of two Neolithic Anatolian horses within the WE Steppe diversity appeared discordant with the MDS analysis. To further investigate this discrepancy, we evaluated whether the NJ tree provided a faithful representation of the pairwise genetic distances. If so, the branch lengths separating each pair of tips in the NJ tree (patristic distances) would be expected to be identical to the corresponding raw pairwise genetic distances. We thus divided patristic and raw pairwise genetic distances of a given horse specimen, relative to any remaining individual, as a measure of the reliability of phylogenetic placement, expecting values equal to one in case of perfect placement. Deviations from one, in contrast, are indicative of phylogenetic mis-placement. For each horse, we then summed up all such ratio deviations, as a proxy for quantifying the goodness-of-fit of the tree to the data ('NJ error' in Fig 1c). The most admixed horses appeared associated with poorer goodness-of-fit, as expected, since NJ inference cannot accommodate gene flow. Neolithic Anatolian horses were found to be amongst those with greatest NJ error, supporting their NJ phylogenetic mis-placement.

Admixture Graph and qpAdm Population Modelling

Struct-f4 as well as the geographic clines of genetic diversity revealed an extremely dynamic evolutionary history, involving substantial gene flow between populations, which cannot be accommodated in bifurcating trees. Adding further complexity, the presence of divergent mtDNA and Y-chromosomal haplotypes (Supplementary Fig 1) indicated the contribution of ghost populations, corresponding to divergent lineages that are not part of our extensive sample set, but still provided significant ancestry to the lineages investigated. The impact of ghost populations was independently reflected by the genetic distance from the DONKEY, which is smaller in recipient lineages relative to those devoid of ghost contribution (Extended Fig 5f). This is especially true for those horses from 2nd-4th mill BCE Iberia (IBE).

In order to accommodate the presence of ghost lineages and admixture, we ran OrientAGraph[19] (version 1.0) to reconstruct horse evolutionary population history in more detail. By providing a fully automated search of the graph space, unbiased to the initial tree, OrientAGraph represents a substantial advancement in admixture graph estimation, while preventing inherent biases associated with supervised inference. For the sake of simplicity, we restricted the analysis to 9 pre-selected groups of potential relevance for DOM2 domestication plus the outgroup, collectively grouping 208 specimens (*ca*. 73% of those included in this study). Their descriptions (and NAMES) correspond to:

- the DONKEY outgroup,
- all DOM2 horses,
- Eneolithic horses from Botai (BOTAI),
- *Equus lenensis* (ELEN) from North-eastern Siberia,
- Upper Paleolithic horses from southern France (Igue du Gral, LP-SFR),
- Horses associated with Corded Ware Complex (CWC),
- Central Pontic-Caspian steppe, encompassing three horses culturally associated with early Yamnaya (Repin), Maykop (Aygurskii) and Potltava (Sosnova) contexts), combined as C-PONT,
- Horses associated with Turganik, late Yamnaya, contexts (TURG),
- Neolithic Anatolian horses (NEO-ANA)

- and the ‘Tarpan’.

Full details on the horse specimens included within each such groups are detailed in SI Table 1.

The 10,205,277 nucleotide transversions were filtered to only retain those present at least once in each of the 9 groups, as well as in the outgroup. This resulted in 7,936,493 fully orthologous variants, that were converted into OrientAGraph[19] input format using a custom pipeline. OrientAGraph[19] was then executed with the maximum likelihood network orientation option (-mlno 1), considering a block size of 100 nucleotide transversions to accommodate linkage disequilibrium and an increasing number of migration edges, from 0 to 5 (-M 0 to 5).

The model assuming no migration was found to explain 99.96% of the overall genetic variance. However, further inspection of the pairwise residuals indicated the existence of yet unmodeled genomic affinities, which required the addition of admixture edges. The first one revealed the introgression of Central European CWC horses into the Tarpan (34.2%), which was placed as a sister lineage to DOM2. This was confirmed by implementing a rotating scheme in qpAdm[18] (version 7.0), whereby two-way admixture models similarly explained the Tarpan as a mixture of CWC (32.2-32.2%) and DOM2 (67.8%) ancestries (SI Table 3). Considering a three-way admixture model added some contribution from TURG horses (TURG: 14.4%; DOM2: 52.5%; CWC: 33.2%). The second migration inferred by OrientAGraph hints at the contribution from one (or multiple) divergent ghost population(s) (here, modelled as a contribution from the outgroup) into the highly-drifted NEO-ANA horses. In the absence of known closely-related lineages in our dataset, the exact contribution of such ghosts into NEO-ANA is difficult to quantify. The third migration edge revealed that BOTAI received ~22.6% introgression from a lineage closest to ELEN, while the remaining 77.4% descend from its common ancestor with NEO-ANA horses. This is in line with the ancestry proportions inferred by Struct-f4 showing that the ancestry component maximized in NEO-ANA was significant in BOTAI but absent within WE steppe horses north of Caucasus. The third migration edge, thus, likely represents early dispersals from Anatolia into CA through a route located South of the Caucasus mountains and the Caspian Sea.

Struct-f4 not only inferred that NEO-ANA spread into CA, but also that NEO-ANA contributed significantly to the genetic makeup of additional lineages located further West and associated with CWC contexts in Germany. This admixture most likely took place following an expansion from Anatolia into Western Carpathians (Hungary), but not from the WE steppes. This is supported by qpAdm modelling, as the Hungarian sample CAR05_Hun_m2458 is consistently modelled as receiving major contribution from a population closely-related to the Turkish sample Kan22_Tur_m2386, regardless of the number of donor populations considered (SI Table 3). This indicates connectivity at least between southern Thrace and Central Europe. It is possible that this (these) wave(s) into Europe originated from Anatolia, but NEO-ANA horses received divergent ghost introgression afterwards, making them an unrecognizable donor, different to its truly ancestral population. Such late ghost influence into NEO-ANA is in line with OrientAGraph inference, from M=2 to M=5. In fact, assuming M=5 migration edges, OrientAGraph accommodated the ancestry of CWC horses right at an intermediate phylogenetic position, *ie*. between LP-SFR horses from Western Europe (France) and a group of horses encompassing WE steppe horses, from which NEO-ANA branched off first (Extended Fig 5e). We evaluated whether such intermediate phylogenetic position was shaped by gene flow or not. Either case, both competing scenarios assume a topology (((DOM2, NEO-ANA), CWC), URAL), which allowed us to leverage the f_4 statistics to test for gene flow. We found a significant excess of ABBA events (77,446), in comparison to BABA counts (69,433) (Z-score=-10.86), supporting that the intermediate CWC placement was indeed shaped by gene flow. That this event remained undetected by OrientAGraph[19], and under the specified running options, likely owes to the nature of the underlying admixture, which formed a cline of ancestry between European and Anatolian horses, and with Central and eastern Europe representing the most intense contact zone. This cline of ancestry was most likely formed following contact over long time periods rather than a single pulse. Indeed, the genetic contribution of ancient Anatolian horses to local horses from Central and eastern Europe, including CWC, possibly corresponds to one (of several) dispersal(s) out-of-Anatolia. This could have commenced at least ~35,000 BCE since a similar ancestry profile was already present in specimen RONPC06_Rom_m34801. This ancestry profile showing NEO-ANA affinities, however, was

not present in the contemporaneous specimen Vert311_Bel_m34314 from Belgium, indicating that past expansions from Anatolia did not reach Belgium at the time. Such continuous gene flow remains difficult to detect through admixture graphs, which model admixture through unidirectional single pulses only.

Regardless of the number of migration edges considered (M=1 to 5), DOM2 was always modelled together with the 'Tarpan' in a group that was most closely related to C-PONT. These were nested within a monophyletic group including TURG, and from which NEO-ANA branched off first. This supported qpAdm modelling (SI Table 3), in which the genetic makeup of DOM2 horses consisted of WE Steppe horses (at least ~95%). Interestingly, the placement of TURG horses more basal to C-PONT is in line with the presence of one ancestry component maximized in NEO-ANA, which was also brought into BOTAI through admixture (M=3 to 5). This ancestry component is not present in C-PONT horses located further West in the Volga-Don region, which indicates secondary contacts between TURG and their geographically closest neighboring populations in the East (*ie*. BOTAI). Combined, our analyses indicate C-PONT horses, and the lower Don-Volga region, as the center for DOM2 domestication.

Direct Ancestry test

The genetic affinities between DOM2 horses and those ancient remains located within WE steppes strongly suggest that this region may have represented the domestication center underlying DOM2 origins. We, thus, formally tested whether Pontic-Caspian horses were in fact directly ancestral to DOM2 horses. To achieve this, we followed the methodology developed by Schraiber[75] and contrasted the statistical support for two competing hypotheses using a Likelihood Ratio Test. The null hypothesis assumes direct ancestry, while the second considers that the most ancient sample was not directly ancestral to DOM2, but represented a sister group that diverged from the true ancestral population.

It is worth noticing that the method requires two sources of information: allele frequencies within DOM2 horses on the one hand, and read counts for putative ancestral samples on the other. While the underlying statistical model can co-estimate error rates for the latter, DOM2 allele frequencies are expected to be error-free. We, thus, decided to apply even stricter filters to our SNP panel in order to exclude potentially residual sequencing errors within DOM2 horses. These consisted of selecting only variants segregating at least as doubletons within our vast collection of DOM2 horses, and retaining only positions that were covered in at least 75% of the DOM2 samples. Pruning linked variants using Plink[76] (version v1.9) with the following parameters, --indep-pairwise 50 10 0.2, resulted in a panel of ~1.4M of extremely high-quality nucleotide transversions, in approximate linkage equilibrium as assumed by the method. Allele frequencies were polarized as ancestral and derived leveraging, for autosomes, a group of modern genomes from seven male specimens of diverse equine species (*E. africanus somaliensis, E. asinus, E. burchelli, E. grevyi, E. hartmannae, E. hemionus onager, E. kiang*[59]), including zebras and asses, according to a majority rule in which positions not shared by at least 2 genomes were disregarded. Results from direct ancestry tests are summarized in SI Table 2.

Out of all the potential ancestors (including the donkeys as negative controls), only two samples could not be rejected as belonging to a population directly ancestral to DOM2. These two specimens, LOR18x15_Rus_m2763 and RN53_Rus_m2785, were radiocarbon-dated to approximately ~4,700 years ago (SI Table 1), and were excavated from the late Yamnaya Turganik settlement (TURG), located in present-day South-West Russia. Importantly, direct ancestry (*ie*. genetic continuity) of Anatolian (or Botai or eastern-European horses) and DOM2 was strongly rejected. Interestingly, the parameters estimated also indicate that the amount of drift shared between DOM2 and NEO-ANA horses (column F in SI Table 2) is comparable to that shared between DOM2 and BOTAI horses, providing indirect but strong and additional evidence on the NEO-ANA and BOTAI affinities.

Predicting the geographic origins of DOM2 horses

The strong patterns of isolation-by-distance observed prior to the third millennium BCE challenge the grouping of individuals into well-delineated populations, and thus compromise the applicability of common methods for modelling population histories in ancient DNA, including

qpAdm and admixture graphs. By providing a predictable extrapolation of allele frequencies across space, however, geographic clines of ancestry can help identify the latitudinal and longitudinal coordinates that most likely underpin the genetic origins of DOM2 horses; *ie*. the geographic center of domestication.

To leverage this simple but powerful principle, we applied Locator[20] (version 1.2), a recently-developed method implementing a deep neural network aimed at predicting the geographic origins of a sample from its levels and patterns of genomic variation, given a panel of geolocated reference populations. This panel was here represented by all non-DOM2 horses (N=136), except the Tarpan and the four Przewalski's horses present in our dataset. To prevent any bias owing to the imputation algorithm implemented within Locator, which was not originally developed to account for the idiosyncrasies of ancient DNA data, we only considered nucleotide transversions covered at least in 75% of the samples. This was aimed to minimize missingness, leaving a total of 3,194,008 SNPs, exceeding the 100,000 validation SNPs used while benchmarking Locator[20], hence providing high resolution data for predicting the most likely genetic homeland of DOM2 horses. The methodology was run using default parameters, except that the width of each neural layer was 512 (instead of 256). The run returning the lowest validation error (mean error = 5.17967 generations of dispersal; median = 4.10985) from a total of 50 independent runs was considered to provide the best prediction. The analysis was repeated for each DOM2 horse as well as the Tarpan and the four Przewalski's horses present in our dataset and the corresponding results are displayed in Fig 3c.

Worldwide expansion of DOM2 horses

That all modern horses descend from the WE Steppe entails a profound implication. It reveals that DOM2 horses expanded well beyond their native range. Our data set confirms that nearly all horse lineages that characterized the horse population structure prior to ~4,200 years ago were replaced by horses characteristic of the DOM2 genomic makeup during the centuries that followed. This suggests that modern domestic horses spread all over Eurasia extremely rapidly after they were domesticated.

We further investigated the expansion of DOM2 horses from their originally geographically-restricted pocket within the steppes of the lower Volga-Don to the rest of Eurasia. To achieve this, we explicitly incorporated the geographic coordinates (longitude, latitude) of all ancient samples, and their age, into the analyses. More specifically, we used the *ncf* package of the R programming language[82] to implement a partial Mantel test, measuring the correlation between geographic and genomic distances over time, in temporal windows of variable span. Time intervals were indeed extended forward in time until encompassing at least 10 samples spread across a large Eurasian range (here defined as a region covering at least two-thirds of the total geographic area identified when considering all sampled specimens together). This filter was feasible thanks to our vast sampling effort, and implemented to minimize regional effects, owing to the over-representation of specific regions within given time intervals. The latter could otherwise confound temporal with geographic patterns of evolution. To measure the area delimited by a set of latitude and longitude coordinates, we used the GeoRange package (version 0.1.0) of the R programming language[85]. Of note, the partial Mantel test corrects for the time-variation within each window, similar in spirit to what described by Loog and colleagues[83], hence, enabling us to adapt window span accordingly to the density of samples over time. Haversine geographic distances between pairs of ancient samples were computed using the geosphere package (version 1.5.10) in R[84], from the corresponding geographic coordinates (SI Table 1), while genomic distances were calculated using the bed2diffs_v1 program. A high correlation coefficient between spatial and genetic distances indicates that the patterns of genomic variation are driven by isolation-by-distance (or by geographic barriers), while absence of correlation suggests limited geographic structure, thus, elevated mobility and migration amongst horse populations.

The analysis was carried out for autosomes and the X chromosome separately, so as to investigate migration regimes were sex-biased or not. Confidence intervals were calculated by sampling with replacement individuals within each time-window (100 bootstrap pseudo-replicates). Bootstrap pseudo-replicates were weighted by the total area covered by the sampled specimens, to maximize the importance of the most informative replicates. Strikingly, geographic and genomic

distances were highly correlated prior to ~5kya, suggesting the presence of highly-structured horse populations at the scale of Eurasia, with a slight increase in mobility by the end of the Last Glacial era (~11.5 kya), followed by further isolation, possibly due to habitat fragmentation and the increase of woodlands during the early Holocene. Isolation by distance totally vanished by the beginning of the 2nd mill BCE, following the rapid globalization of DOM2 horses. This shift in mobility was unprecedented in the previous 20 millennia. There, both the autosomes and the X chromosome showed nearly indistinguishable trajectories, suggesting no strong asymmetry between male and female migration regimes during this process.

To further characterize the drivers for the strong geographic structure found prior to 5kya, we ran EEMS[16] (built with Eigen version 3.2.2 and Boost version 1.57, and using rEEMSplots version 0.0.0.9000) conditioning on the time windows pre-dating the DOM2 expansion (>5 kya), during the 3rd mill BCE, and post-dating their expansion (*ie*. the last 4 ky). The EEMS MCMC was run for 50M iterations, with a burn-in period of 15M. Likelihood trajectories revealed robust convergence, and validation plots exhibited an excellent correlation between the observed and fitted genetic dissimilarities. Interestingly, the genomic distance increased almost linearly with the geographic distance for pre-expansion horses, regardless of their age, while this trend vanished for post-expansion DOM2 horses (Fig 2). This was true everywhere except in Iberia, where IBE horses continued to exist at least until ~3,900 years ago (Spain39_Spa_m1968). Overall, this mirrors the patterns of isolation-by-distance described above. Interestingly, the migration barriers inferred by EEMS overlaid with important geographic features, such as the Caspian Sea, the Black Sea, the Alps, the Pyrenean mountain range and the Pamir-Alai and Altai mountain ranges. This suggests that the population structure in place prior to horse domestication was shaped by natural geographic barriers. It is noteworthy that EEMS migration surfaces were $\log_{10}$ transformed but not mean-centered as implemented by default, to allow immediate visual comparison of the scale of the migrations inferred. In order to facilitate tracking the spread of the ancestry components prior to and during the last four millennia, pie-charts depicting the ancestry proportions inferred by Struct-f4 were overlaid on the migration surfaces, using the draw.pie R function from the mapplots package[81] (version 1.5.1). The size of each individual pie-chart was commensurate with the number of samples excavated from the same geographic coordinates.

F_{ST} selection scans

To investigate the genetic changes that may be associated with the explosive success of DOM2 horses ~2,000 BCE, we compared the frequency of each nucleotide transversion (n=10,205,277), in DOM2 and non-DOM2 horses. The high and even sample sizes for both groups provide unprecedented resolution into the patterns of allele frequency differentiation (N=141 and N=142, respectively). Additionally, non-DOM2 horses include all known genetic lineages pre-dating DOM2 domestication and offer, thus, an exhaustive reference panel for the genetic variation preceding domestication.

Weir and Cockerham F_{ST} index was calculated with Plink[76] (version 1.9), and the results were integrated and visualized together with external genomic tracks using the GViz R package[86] (version 1.36.2). Two outlier regions stood out as extremely differentiated in DOM2 horses. The first was located within the EquCab9:72,810,000-73,734,000 region and encompasses a narrow region clearly peaking around the *GSDMC* gene promoter, where it reaches F_{ST} values as high as 0.87. Although marginally overlapping the first *GSDMC* exon for a few isoforms, the vast majority of this F_{ST} peak indeed lays immediately upstream of the gene. Interestingly, the whole orthologous region in humans, mice and cows corresponds to an intron, not to the gene promoter as in modern domestic horses. The F_{ST} peak is also surrounded by two independent insertions of transposable elements in the EquCab3 reference assembly. These LINEs are likely present in DOM2 horse genomes only, given that non-DOM2 samples show extremely limited sequence coverage within those regions.

The second F_{ST} outlier region is located with EquCab3:30,216,000-46,667,500 and spans 16Mb and 138 protein-coding genes. It delineates a nearly symmetric increase in genetic differentiation that peaks close to the *ZFPM1* gene, with F_{ST} values of 0.73 (for the nucleotide transversion EquCab3:35,709,483). The orthologous base is located within an intron of the *ZFPM1* gene in other mammal species. Only shorter isoforms are, however, currently annotated for the EquCab3 assembly,

leaving this position 20 Kb upstream of the *ZFPM1* transcription start site. Sequence coverage was found on par in DOM2 and non-DOM2 samples across the whole region.

Coat-color variation alleles

Animal domestication, including horse domestication, has been traditionally associated with an explosion of coat-coloration patterns[255]. Our extensive genome panel covers time periods and locations that have not been previously examined. We, thus, undertook this opportunity to track the alleles carried by each individual genome for a set of 43 loci associated with or causative for various coat colors and patterns. For biallelic SNPs, we simply counted the proportion of reads supporting the causative allele. Results were summarized in a heatmap, ordered as the NJ tree in Fig 1b, where the color intensity scales either with the proportion of supporting reads (for SNPs) (Extended Fig 8).

This phylogenetic ordering revealed limited shifts in allele frequencies between clades, as DOM2 and non-DOM2 horses. For example, the EquCab3:36,979,560 variant located within the *MC1R* gene and causative for chestnut coat coloration was previously inferred to have evolved under positive selection from early domestication stages[256]. While almost exclusive to DOM2 horses, this variant did not raise in frequency until recent times, in line with more recent work reporting an extensive ancient genome panel[5]. Interestingly, our data confirm that mutations at the *TRPM1* gene that are associated with Leopard spotting complex and night blindness in homozygous state[257] segregated at moderate frequencies within Botai horses. It revealed that they were absent amongst their closest relatives, including from sites located in the Altai range (Novoilinka-III) and the Urals (Aleksandrovskoe IV). As night blindness reduces the individual fitness as the animal is more subject to night predation, the presence of the allele may support previous interpretations that Botai horses were domesticated[3].

Supplementary Discussion 1. Linguistic index of Indo-European equine and Indo-Iranian chariotry terminology

The following four SI Tables provide a linguistic index on (1) horse-related terms in Indo-European languages (SI Table 4), (2) chariotry terminology in Indo-Iranian languages (SI Table 5), (3) horse epithets in Indo-Iranian languages (SI Table 6) and (4) coat chromonyms applied to horses (SI Table 7). The information is compiled from the literature[258-264].

SI Table 4. Proposed Proto-Indo-European horse-related terms with cognates in Europe and Asia (phylogenetically stratified)

PIE	Anatolian	Tocharian	Germanic	Celtic	Italic	Greek	Armenian	Balto-Slavic	Indic	Iranian	
**h_1eḱ-u(o)-*	HLuw. *ásu-* 'horse'	A *yuk*, B *yakwe* 'horse'	ON *jór*, OE *eoh* 'horse'	OIr. *ech* 'horse'	Lat. *equus* 'horse'	ἵππος 'horse, mare'	*ēš* 'ass'		Skt. *áśva-* 'horse'	YAv. *aspa-*, OP *asa-*, Khot. *aśśa-* 'horse'	Indo-European (+ Anatolian)
**h_1eḱ-ueh_2-*					Lat. *equa* 'mare'			Lith. *ašvà* 'mare'	Skt. *áśvā-* 'mare'	Av. *aspā-* 'mare'	
**p(ō)lH-*			Go. *fula* 'foal'	W *ebawl* 'foal'		πῶλος 'foal'	*ul* 'kid'?				Indo-European - Anatolian - Tocharian
**ḱŏpH-o-*			ON *hófr*, OE *hōf* 'hoof'						Skt. *śaphá-* 'hoof'	YAv. *safa-*, Khot. *saha-* 'hoof'	
**uol-o-*								Lith. *vãlas* 'horsetail hair'	Skt. *vā́ra-*, *vā́la-* '(hair of the) tail, horse-hair'		
**su-leh_2-*								OPru. *sulo* 'curdled milk'	Skt. *súrā-* 'alcoholic drink'	YAv. *hurā-*, MP *hur* 'alcoholic drink, koumiss'	Balto-Slavic + Indo-Iranian
**b^hrod^h-no-*								CS *bronъ* 'white, greyish'	Skt. *bradhná-* 'pale red, ruddy, bay'		

(Shading is applied to terms with semantics relatable to horses.)

SI Table 5. Indo-Iranian chariotry terminology

Proto-Indo-Iranian	Sanskrit	Avestan	Indo-Iranian other	IE other
**HratHa-*	*rátha-* ‘chariot’	*raθa-* ‘chariot’	Khot. *rraha-* ‘chariot’	
**HratHiH-*	*ráthī́-* ‘charioteer’	*raiθī-* ‘charioteer’		
**HratHaištaH-*	*ratheṣṭhā́-* ‘chariot fighter’	*raθaēštā-* ‘chariot warrior’		
**Haru̯ant-*	*árvant-* ‘race-horse’	*auruuaṇt-* ‘race-horse’		
**i̯aH-*	*yā́-* ‘drive’	*yā-man-* ‘course’		ToA *yā-* ‘go, travel’, Lith. *jóti* ‘drive, go’
**Hraćanā-*	*raśanā́-* ‘cord, bridle’		MP *lsn* ‘rope’	
**Hiauktra-*	*yóktra-* ‘thong, yoking cord’	*yaoxəδra-* ‘halter, bridle’		
**Hab^{h}i-d^{h}aHana-*	*abhidhā́nī-* ‘horse-halter’	*aiβiδāna-* ‘bridle’	Khot. *byāna-* ‘bridle’	

SI Table 6. Indo-Iranian epithets involving horses

Proto-Indo-Iranian	Sanskrit	Avestan	Indo-Iranian other	IE other
**Hāću̯a-Haću̯a-*	*āśvàśva-* ‘having quick horses’	*āsu.aspa-* ‘id.’		Cf. Gk. ὠκέες ἵπποι ‘swift horses’
**Hi̯ukta-Haću̯a-*	*yuktā́śva-* ‘having yoked horses’	*yuxta.aspa-* ‘id.’		
**Hrjra-Haću̯a-*	*r̥jrā́śva-* ‘with fast horses’	*ərəzrāspa-* ’id.’		
**Hsu-Haću̯a-*	*sváśva-* ‘having good horses’	*huu-aspa-* ‘id.’		
**Hu̯i-šHta-Haću̯a-*	Skt. *víṣitāso áśvāḥ* ‘unbound horses’	*Vīštāspa-* (proper name)	OP *Vištāspa-* (proper name)	
**kšipra-Haću̯a-*	*kṣiprāśva-* ‘with swift horses’	*xšuuiβrāspa-* (proper name)		

SI Table 7. Indo-Iranian coat chromonyms applied to horses

Proto-Indo-Iranian	Sanskrit	Avestan	Indo-Iranian other	IE other
**b^{h}ab^{h}ru-*	*babhrú-* ‘reddish brown, brown’		Mit. *b/papru-nnu* ‘epithet of horses’	
**b^{h}rad^{h}na-*	*bradhná-* ‘pale red, ruddy, bay’			CS *bronъ* ‘white, greyish (of horses)’
**Haruša-*	*aruṣá-* ‘reddish, white’	*auruša-* ‘white’		OHG *elo* ‘sallow’
**j^{h}ari-*	*hári-* ‘yellowish, bay’	*zairi-* ‘yellow’		
**kadru-*	*kádru-* ‘reddish brown’	*kadrva.aspa-* (placename)		
**palHuša-*	*paruṣá-* ‘grey(-brown)’	*pouruša-* ‘grey’		E *fallow*
**parita-*	*palitá-* ‘grey’		Mit. *p/baritta-nnu* ‘grey (of horses)’	
**pingara-*	*piṅgalá-* ‘reddish’		Mit. *p/binkara-nnu* ‘reddish (of horses)’	
**pršant-*	*pŕ̥ṣant-* ‘dappled’	*paršaṯ.gauu-* (proper name)		

Supplementary Notes

92. Farka, C. Die Abteilung für Bodendenkmale des Bundesdenkmalamtes. Jahresbericht 2008. *Fundberichte aus Österreich* **47**, 9–96 (2008).
93. Germonpré, M. Influence of climate on sexual segregation and cub mortality in Pleniglacial cave bear. The Future from the Past. Archaeozoology in Wildlife Conservation and Heritage Management (2004).
94. Pleinerová, I. Březno. Osada lidu knovízké kultury v SZ Čechách. Ústí nad Labem: Severočeské nakladatelství (1988).
95. Peške, L. Knovízský osteologický materiál. In: Březno. Osada lidu knovízké kultury v SZ Čechách (ed. Pleinerová, I.) Ústí nad Labem: Severočeské nakladatelství, pp. 59–65 (1988).
96. Řídký, J., Stolz, D., Zápotocká, M. Neolitické osídlení v Černém Vole (Praha-západ). Formy objektů, keramika a štípaná industrie z výzkumů 1975–77 a 1914. In: Praehistorica XXVIII. Praha: Univerzita Karlova, 177-236 (2009).
97. Daněček, D. Holubice, k. ú. Holubice v Čechách, výstavba rodinných domů (č. př. 10/2005). *Středočeský vlastivědný sborník* **24**, 137 (2006).
98. Kovačiková, L., Daněček, D. Užitkový význam hospodářských zvířat na neolitickém sídlišti v Holubicích. In: Beneš, J., Pokorný, P. (eds.), Bioarcheologie v České republice. České Budějovice – Praha: University of South Bohemia in České Budějovice – Institute of archaeology of the Czech Academy of Sciences, pp. 177–198 (2008).
99. Pleinerová, I. Litovice, k. ú. Hostivice. Středočeský vlastivědný sborník 23, 125 (2005).
100. Kyselý, R. Archeozoologická problematika eneolitu Čech. Dissertation. Praha: Faculty of Science, Charles University (2010).
101. Svoboda, J. & Šmíd, M. Dílenský objekt kultury nálevkovitých pohárů na Stránské skále. *Pravěk - Nová řada* **4**, 79–125 (1994).
102. Kyselý, R. & Peške, L. Horse size and domestication: Early equid bones from the Czechia in the European context. *Anthropozoologica* **51**, 15-39 (2016).
103. Hložek, J. Tuchoměřice, výstavba komunikací obytného souboru 24 rodinných domů. *Středočeský vlastivědný sborník* **24**, 133–135 (2006).
104. Sůvová, Z. & Hložek, J. Tuchoměřice: archeozoologická analýza materiálu ze sídliště mladší doby bronzové (kultura knovízská/štítarská). In: Doba popelnicových polí a doba halštatská. Brno: Ústav archeologie a muzeologie Filozofické fakulty Masarykovy university, pp. 315–322 (2007).
105. Hložek, J. & Smíšek, K. Tuchoměřice, k. ú. Kněžívka, výstavba komunikace pro obchodní centrum 'Outlet Airport Praha'. *Středočeský vlastivědný sborník* **26**, 111–112 (2008).
106. Limburský, P., Brnič, Ž., Dobeš, M., Flašar, J., Kleinová, K., Kovačiková, L., Koštová, N., Kyselý, R., Likovský, J., Pleinerová, I., Salač, V., Stránská, P., Trojánková, O., Vélová, L. Pohřební areály únětické kultury ve Vliněvsi. Praha: Institute of archaeology of the Czech Academy of Sciences (2018).
107. Dobeš, M., Stránská, P. Křivánek, R., Limburský P. Časně eneolitické ohrazení ve Vliněvsi: příspěvek k povaze kontaktu mezi jordanovskou a michelsberskou kulturou v Čechách. *Památky archeologické* **107**, 51–115 (2016).
108. Rasmussen, U. Four minor Pitted Ware culture sites on Djursland. Their individual character function. in The Pitted Ware Culture on Djursland. Supra-regional significance and contacts in the Middle Neolithic of southern Scandinavia. In: ed. Klassen, L. Aarhus University Press and East Jutland Museum, pp. 141–242 (2020).
109. Lang, V. Settlement Sites and Settlement in the Late Bronze and Early Iron Ages. In: The Bronze and Early Iron Ages in Estonia. Tartu University Press Tartu, pp. 60–62 (2007).
110. Lõugas, L. Subfossil vertebrate fauna of Asva site, Saaremaa. Mammals. *Stilus* **5**, 71–93 (1994).
111. Lang, V. The Bronze and Early Iron Ages in Estonia. In: Settlement Sites and Settlement in the Late Bronze and Early Iron Ages. University of Tartu Press, pp. 63–65 (2012).
112. Bodu, P. "*Magdalenians – Early Azilians in the centre of the Paris Basin : A filiation ? The example of Le Closeau (Rueil-Malmaison, France)*". The organization of lithic technology

in late glacial and early post glacial Europe. Ed. Sarah Miliken. British Archaeological Reports, *Series* **700**, pp. 131-147 (1998).
113. Bodu, P., & Bemilli, C. Le gisement du Closeau à Rueil-Malmaison (Hauts-de-Seines) : le lion est-il mort ce soir ? Actes du colloque international de Besançon. Les derniers chasseurs-cueilleurs d'Europe occidentale, Besançon, octobre 1998. Presses Universitaires Franc-comtoises. *Annales littéraires* **699**, pp. 173-185 (2000).
114. Bignon, O. Chasser les chevaux à la fin du Paléolithique dans le Bassin parisien. Stratégies de subsistance et des modes de vie au Magdalénien et à l'Azilien ancien. *BAR International Series* **1747**, pp. 170 (2008).
115. Bignon, O. & Bodu, P. Stratégie cynégétique et mode de vie à l'Azilien ancien dans le Bassin parisien : les apports de l'exploitation des chevaux du Closeau (niveau inférieur ; Rueil-Malmaison, Hauts-de-Seine). *Anthropologie* **110**, 401–417 (2006).
116. Bignon-Lau, O. About Early Azilian Lifeway in the Paris Basin: Economical and Spatial from zooarchaeological data. in From the Atlantic to beyond the Bug - Finding and defining the Federmesser-Gruppen/Azilian on the North European Plain and adjacent areas. In: eds. Grimm, S. B., Mevel, L., Sobkovoak-Tabaka, I. & Weber, M. J. Mainz, RGZM Tagungen, pp. 35–60 (2019).
117. Castel, J.-C. *et al*. Animal exploitation strategies in eastern aquitaine (France) during the last glacial maximum. Wild Things. Recent Advances in Palaeolithic and Mesolithic Research. Oxbow Books, Oxford, pp. 160–174 (2014).
118. Nickels, A. La Monédière à Bessan (Hérault). Le bilan des recherches. *Documents* **12**, 51–119 (1989).
119. Motzenbäcker, I. Ausgrabung einer mehrschichtigen bronze- und eisenzeitlichen Siedlung: Tachti Perda, Kachetien, Georgien. In: Aktuelle Forschungen in Eurasien, Deutsches Archäologisches Institut, Berlin, pp. 66-67 (2014).
120. Seregély, T *et al.* Archäologische Forschungen zur prähistorischen Nutzung von Felstürmen und Höhlen auf der Nördlichen Frankenalb, Oberfranken. In: Beiträge zur Archäologie in Ober- und Unterfranken. Band 8. Verlag Dr. Faustus, pp. 29-52 (2013).
121. Seregély, T. Wattendorf-Motzenstein: eine schnurkeramische Siedlung auf der Nördlichen Frankenalb. Studien zum dritten vorchristlichen Jahrtausend in Nordostbayern. In: Endneolithische Siedlungsstrukturen in Oberfranken I, Verlag Rudolf Habelt, pp. 126-128 (2008).
122. Müller, J. & Seregély, T. Wattendorf-Motzenstein: eine schnurkeramische Siedlung auf der Nördlichen Frankenalb. Naturwissenschaftliche Ergebnisse und Rekonstruktion des schnurkeramischen Siedlungswesens in Mitteleuropa. In: Endneolithische Siedlungsstrukturen in Oberfranken II, Verlag Rudolf Habelt, pp.196 (2008).
123. Seregély, T. Die Rothensteine bei Stübig: Felsturmopferplatz und schnurkeramische Steilhangsiedlung. In: Hohler Stein, Rothensteine und Jungfernhöhle. Archäologische Forschungen zur prähistorischen Nutzung naturheiliger Plätze auf der Nördlichen Frankenalb, eds. Falkenstein, F. Würzburg, pp. 44-55 (2012).
124. Sørensen, M. L. S. & Rebay-Salisbury, K. Landscapes of the body: Burials of the Middle Bronze Age in Hungary. European. *J Arc* **11**, 49–74 (2007).
125. Poroszlai, I. The conservation and exhibition of archaeological remains: archaeological parks and experimental archaeology. In: Hungarian archaeology at the turn of the Millennium. Budapest, pp. 432–435 (2003).
126. Gál, E. Animals at the Dawn of Metallurgy in South-Western Hungary. Institute of Archaeology, Research Centre for the Humanities. Hungarian Academy of Sciences, MTA BTK Régészeti Intézet, Budapest, pp. 288 (2017). http://real.mtak.hu/74515/
127. Dyson, R. H. The Iron Age architecture at Hasanlu: an essay. *Expedition* **31**, 107 (1989).
128. Dyson, R. H. The Achaemenid painted pottery of Hasanlu IIIA. *Anatolian Studies* **49**, 101–110 (1999).
129. Negahbān, E. O. Sagzabad Excavation Report. *Iran* **12**, 216 (1974).
130. Bagnasco Gianni, G., Garzulino, A., Kay, S., Marzullo, M. & Smith, C. J. Civita di tarquinia (comune di tarquinia, provincia di viterbo, regione lazio). *Pap Br Sch Rome* **86**, 328-332 (2018).
131. Kukushkin, I. A. Research of the Aschisu burial ground. Kurgan 2. In: Kadyrbayev readings. Aktobe, pp. 86–91 (2007).

132. Logvin, A. V. & Shevnina, I. V. Regarding a Sintashta burial complex of the Bestamak burial ground. In: Materials of the international scientific conference 'Archaeology of Kazakhstan in the era of independence: results, prospects'. Kustanai University, Institute of archaeology, eds. A. H. Margulan, pp. 349–359 (2011).
133. Merz, V. K. Studies of the Borly settlement in 2017 and prospects for studying the Neolithic of North-East Kazakhstan. In: Proceedings of the International Scientific Conference 'Margulanov Readings - 2018. Spiritual Modernization and Archaeological Heritage' Kustanai University, Institute of archaeology, eds. A. H. Margulan, pp. 111–115 (2018).
134. Logvin, A. V. & Shevnina, I. V. Study of the Sintashta burial ground Karatomar, Kurgan 1 (preliminary report). In: XXI Ural archaeological meeting dedicated to the 85th anniversary of the birth of G.I. Matveeva and the 70th anniversary of the birth of I.B. Vasiliev, pp. 123–125 (2018).
135. Kalieva, S. S. & Logvin, V. N. Skotovody Turgaya v tret'em tysyacheletii do nashei ery. Kustanai University, Institute of archaeology, eds. A. H. Margulan, Kustanai (1997).
136. Motuzaite Matuzeviciute, G. *et al.* Climatic or dietary change? Stable isotope analysis of Neolithic–Bronze Age populations from the Upper Ob and Tobol River basins. *Holocene* **26**, 1711–1721 (2016).
137. Anthony, D. W. & Brown, D. R. Eneolithic horse exploitation in the Eurasian steppes: diet, ritual and riding. *Antiquity* **74**, 75–86 (2000).
138. Merz, I. V. The culture of the population of East Kazakhstan in the Early Bronze Age. Altai State University (2017).
139. Chechushkov, I. V., Usmanova, E. R. & Kosintsev, P. A. Early evidence for horse utilization in the Eurasian steppes and the case of the Novoil'inovskiy 2 Cemetery in Kazakhstan. *J Arc Sci Rep* **32**, 102420 (2020).
140. Merz, V. K. Mesolithic complexes of the Shiderty 3 site. *Vestnik KazNU Historical Series* **1**, 108–110 (2007).
141. Croitor, R. & Sîrbu, G. Animal remains from the Late Eneolithic settlement of Gordineşti II-Stînca goală (Edineț district). Part 1. *Revista Arheolică serie nouă* **XIII**, 215-219 (2017).
142. Sava, E. & Kaiser, E. Die Siedlung mit 'Aschehügeln' beim Dorf Odaia-Miciurin, Republik Moldova. Archäologische und naturwissenschaftliche Untersuchungen. Academiei de Ştiințe Moldova. Chișinău : Muzeul Național de Arheologie și Istorie a Moldovei, pp. 532 (2011).
143. Khudyakov, Y. S. Khereksury i olennyye kamni. in Arkheologiya, etnografiya i antropologiya Mongolii. Nobosibirsk: Nauka, pp. 136–162 (1987).
144. Iderkhangai, T.-O. *et al.* 2015 Archaeological Rescue Excavation Report on the Egiin Gol Hydroelectric Dam, Khutag Undur Sum, Bulgan Aimag. Ulaanbaatar State University. Ulaanbaatar, Mongolia, pp. 67–150 (2015).
145. Kusliy, M. A. *et al.* Traces of Late Bronze and Early Iron Age Mongolian Horse Mitochondrial Lineages in Modern Populations. *Genes* **12**, 412(2021)
146. Tishkin, A. A. "Deer" stones of Mongolia and adjacent territories as one of the indicators of the archaic nomadic empire (to the formulation of the question). In: V (XXI) All-Russian Archaeological Congress. Barnaul: Altai State University, pp. 1026–1026 (2017).
147. Lepetz, S. *et al.* Customs, rites, and sacrifices relating to a mortuary complex in Late Bronze Age Mongolia (Tsatsyn Ereg, Arkhangai). *Anthropozoologica* **54**, 151–177 (2019).
148. Kovalev, A. A., Erdenebaatar, D. & Rukavishnikova, I. V. A ritual complex with deer stones at Uushigiin Uvur, Mongolia: composition and construction stages. Archaeology, Ethnology and *Anthropology of Eurasia* **44**, 82–92 (2016).
149. Tishkin, A. A. Advancing Archaeological Research of the Mongolian Altai through the Scientific Study of Deer Stones: New Discoveries from Buyant Valley. *Asian Perspectives* **59**, 453–477 (2020).
150. Bednarczyk, J. Na pograniczu światów: studia z pradziejów międzymorza bałtycko-pontyjskiego ofiarowane Profesorowi Aleksandrowi Kośko w 60. rocznicę urodzin. In: Poznań: Wydawnictwo Poznańskie, pp. 595 (2008).
151. Moskal-del Hoyo, M. *et al.* The continuous persistence of open oak forests in the Miechów Upland (Poland) in the second half of the Holocene. *Quat Int* **458**, 14–27 (2017).

152. Nowak, M., Hoyo, M. M.-D., Mueller-Bieniek, A., Lityńska-Zając, M. & Kotynia, K. Benefits and weaknesses of radiocarbon dating of plant material as reflected by Neolithic archaeological sites from Poland, Slovakia and Hungary. *Geochronometria* **44**, 188–201 (2017).
153. Korczyńska M., Cappenberg K., Nowak M., Szwarczewski P., Moskal-del Hoyo M. Multi-methodological approaches to investigate large archaeological sites: The case study of the Eneolithic settlement in Mozgawa, western Lesser Poland. *J Arc Sci Rep* **27**, 101941 (2019).
154. Moskal-del Hoyo, M. *et al.* Open country species persisted in loess regions during the Atlantic and early Subboreal phases: New multidisciplinary data from southern Poland. *Rev. Palaeobot. Palynol.* **253**, 49–69 (2018).
155. Dumitrescu V. *Stratigrafia aşezării-tell de pe Ostrovelul de la Căscioarele,* Cultură. *şi civilizație la Dunărea de Jos* **1**, 73- 81 (1986).
156. Bălășescu A., Moise D., Radu V. *The palaeoeconomy of Gumelnița communities on the territory of Romania*, Cultură. In: şi Civilizație la Dunărea de Jos, XXII, In Honoreum Silvia Marinescu-Bîlcu, pp. 167-206 (2005).
157. Haimovici, S. Materialul faunistic de la Girbovat. Studiu arheozoologic. *Arheologia Moldovei* **14**, 153–166 (1991).
158. Păunescu, A. Paleoliticul şi mezoliticul din spațiul transilvan: studiu monografic. Bucuresti: Agir, pp. 573 (2001).
159. Hansen, S. et al. Pietrele am "Lacul Gorgana". Bericht über die Ausgrabungen in der neolithischen und kupferzeitlichen Siedlung und die geomorphologischen Untersuchungen in den Sommern 2012–2016. *Eurasia Antiqua* **20**, 1-116 (2017).
160. Bokovenko, N. A., Plasteeva, N. A. & Tishkin, A. A. Horses from the Arzhan-1 Mound: Results of Archaeological Research and Morphometric Analysis of the Preserved Osteological Collection. *Povolzhskaya Arkheologiya (The Volga River Region Archaeology)* **3**, 219–232 (2020).
161. Čugunov, K., Parzinger, H. & Nagler A. Der skythenzeitliche Fürstenkurgan von Aržan 2 in Tuva. Archäologie in Eurasien 26, Steppenvölker Eurasiens 3. Mainz: Philipp von Zabern (2010).
162. Gryaznov, M. P. Arzhan. Arzhan: Tsarskii kurgan ranneskifskovo vremeni (Arzhan: The Tsar Kurgan of the Early Scythian Time). Leningrad: Nauka (1980).
163. Korenevskiy, S. N. & Kalmykov, A. A. Maykopskie pogrebeniya kurgana 22 mogilnika Aygurskiy 2. *Rossiyskaya Arkheologiya*, 77–94 (2017).
164. Gerasimova, M. M., Pezhemskiy, D. V. & Jablonskiy, L. T. Paleoantropologicheskie materialy maykopskoy epokhi iz Zentralnogo Predkavkazya. Materialy po Izucheniyu IstorikoKulturnogo Naslediya Severnom Kavkaza 91–121 (2007).
165. Seregin, N. N. et al. Research in the Chemalsky District', Archaeological discoveries 2017, 484–486 (2019).
166. Tishkin, A. A. and Seregin, N. N. Objects of horse equipment from the barrows of Biiken culture of Northern Altai. In: Bazarov, B. V. and Kradin, N. N. eds, Nomadic Empires of Eurasia in archaeological and interdisciplinary studies. Ulan-Ude: BSC SB RAS, pp. 87–90 (2019).
167. Kosintsev, P. A. Animals in the sacrificial complexes of the Bolshekaragansky burial ground. In: Kadyrbayev readings. Aktobe, pp. 50–54 (2010).
168. Khalyapin, M. V. The first burial mound of the Sintashta culture in the Steppe Ural Bronze Age of Eastern Europe: characteristics of cultures, chronology and periodization. In: Conference materials: 'By the centenary of the periodization of V.A. Gorodtsov Bronze Age Eastern Europe', pp. 417–425 (2001).
169. Chugunov, K. V., Parzinger, H. & Nagler, A. Chronology and Cultural Affinity of the Kurgan Arzhan-2 Complex According to Archaeological Data. In: Impact of the Environment on Human Migration in Eurasia. Springer: Netherlands, pp. 1–7 (2005).
170. Chugunov, K. V. Exploration of the Chinge-Tey I burial and funerary complex in Tuva. *Archaeological Papers of the State Hermitage Museum* **42**, 92–109 (2019).
171. Bessudnov, A. N., Bessudnov, A. A., Burova, N. D., Lavrushin, Y. A. & Spiridonova, E. A. Nekotorye rezul'taty issledovanii paleoliticheskikh pamiatnikov u khutora Divnogor'ye na

srednem Donu (2007--2011 gg.)[some Results of Investigations of Palaeolithic sites Near Divnogorie Farmstead on the Middle Don (seasons 2007--2011)]. *Kratkie soobshcheniia Instituta arkheologii* 144–154 (2012).
172. Sycheva, S. A. *et al.* Divnogorie pedolithocomplex of the Russian Plain: Latest Pleistocene deposits and environments based on study of the Divnogorie 9 geoarchaeological site (middle reaches of the Don River). *Quat Int* **418**, 49–60 (2016).
173. Galkina, L. I. & Ovodov, N. D. Anthropogenic theriofauna of Western Altai caves. In:Taxonomy, fauna, zoogeography of mammals and their parasites. Novosibirsk: Nauka, pp. 165–180 (1975).
174. Vasiliev, S. K., Ovodov, N. D. & Martynovich, N. V. New paleotheriological studies of the Hyena's Lair Cave (North-Western Altai). *Problems of Archaeology Ethnography Anthropology of Siberia and Neighboring Territories* **12**, 43–49 (2006).
175. Epimakhov, A. V. Southern Trans-Urals in the Middle Bronze Age. Edited by N. B. Vinogradov. Chelyabinsk: A. Miller Library (2002).
176. Epimakhov, A. V. Early complex societies of the North of Central Eurasia (based on the materials of the Kamennyi Ambar-5 burial ground). Chelyabinsk: Chelyabinsk Printing House (2005).
177. Hanks, B. K., Epimakhov, A. V. & Renfrew, A. C. Towards a refined chronology for the Bronze Age of the southern Urals, Russia. *Antiquity* **81**, 353–367 (2007).
178. Gening, V. F., Zdanovich, G. B. & Gening, V. V. Sintashta: archeological sites of Aryan tribes of the Ural-Kazakh Steppes. Chelyabinsk: South Ural Book Publishing House (1992).
179. Ventresca Miller, A. *et al.* Weaning practices among pastoralists: New evidence of infant feeding patterns from Bronze Age Eurasia. *Am J Phys Anthropol* **162**, 409–422 (2017).
180. Hanks, B. *et al.* Bronze Age diet and economy: New stable isotope data from the Central Eurasian steppes (2100-1700 BC). *J Arc Sci* **97**, 14–25 (2018).
181. Judd, M. A. *et al.* Life in the fast lane: Settled pastoralism in the Central Eurasian Steppe during the Middle Bronze Age. *Am J Hum Biol* **30**, e23129 (2018).
182. Dudgeon, J. V. *et al.* Investigating Biogenic Versus Diagenetic Trace Element Incorporation in Archaeological Mineralized Tissues with LA-ICP-MS. In: Recent Advances in Laser Ablation ICP-MS for Archaeology. Natural Science in Archaeology. Berlin, Heidelberg: Springer-Verlag, pp. 323–341 (2016).
183. Gayduchenko, L. L. Organic remains from fortified settlements and necropoli of the "Country of Towns". In: Complex societies of central Eurasia from the 3rd to the 1st millennium BC: regional specifics in light of global models. Washington: Institute for the Study of Man, pp. 400–418 (2002).
184. Kosintsev, P. A. Animals in the burial rite of the population of the Volga-Urals area in the beginning of the 2nd millennium BC. *J. Indo-European studies* **45**, 232–248 (2002).
185. Rogachev, A. N. Mnogosloinye stoyanki Kostenkovsko-Borshevskogo raiona na Donu i problema razvitiya kul'tury v epokhy verkhnego paleolita na Russkoi Ravnine. *Materialy i issledovaniya po arkheologii SSSR* **59**, 9–134 (1957).
186. Hoffecker, J. F. *et al.* Evidence for kill-butchery events of early Upper Paleolithic age at Kostenki, *Russia. J Arc Sci* **37**, 1073–1089 (2010).
187. Klein, R. G. Man and Culture in the Late Pleistocene: A Case Study. Chandler Publishing Company, pp. 296 (1969).
188. Anthony, D. W., Brown, D. R., Kuznetsov, P. F.& Mochalov, O. D. Excavations at the LBA settlement at Krasnosmarskoe. In: A Bronze Age Landscape in the Russian Steppes: The Samara Valley Project. Anthony D. W., Brown, D. R., Mochalov, O. D., Khokhlov, A. A., Kuznetsov, PA. F. eds Cotsen Institute of Archaeology Press: UCLA, pp. 227-290 (2016).
189. Marsadolov, L. S. Monuments of early nomads in Ust-Kuyum in Altai (based on excavations by GP Sosnovsky and GP Sergeev). *Archaeological Papers of the State Hermitage Museum* **22**, 11–22 (1981).
190. Tishkin, A. A. Biykenskaya kul'tura Altaya arzhano-mayemirskogo vremeni: soderzhaniye i opyt periodizatsii. In: 'Terra Scythica': Materialy mezhdunarodnogo simpoziuma. Molodin, V. I. & Khansen, S. eds., Nobosibirsk: IAE SO RAN pp. 272–290 (2011).
191. Svendsen, J. I. *et al.* Geo-archaeological investigations of Palaeolithic sites along the Ural Mountains--on the northern presence of humans during the last Ice Age. *Quat Sci Rev* **29**, 3138–3156 (2010).

192. Kosintsev, P. A. & Bachura, O. P. Late Pleistocene and Holocene mammal fauna of the Southern Urals. *Quat Int* **284**, 161–170 (2013).
193. Myshkin, V. N. & Turetskiy, M. A. Kurgany bronzovogo veka na reke Malyy Kinel'. *Voprosy arkheologii Povolzh'ya* **4**, 314–334 (2006).
194. Kiryushin, Y. F. *et al.* Archaeobotanical and phytolithic studies at the Novoilinka-3 settlement (Northern Kulunda)'. *Tomsk State University Journal of History* **4**, 10–14 (2013).
195. Kiryushin, K. Y. et al. Comprehensive studies of the Novoilinka-VI settlement in 2014. *Bulletin of the Altai Science* **1**, 70–75 (2015).
196. Kiryushin, K. Y. and Gaiduchenko, L. L. Articles of bones in the materials of the first layer Eneolithic settlement "Novoilinka-VI". *Theory and Practice of Arc. Research* **3**, 25–43 (2016).
197. Erokhin, N. G., Tikhonova, N. R. & Volkov, R. B. Pershinskaya I cave - a stratified archaeological site in the Middle Trans-Urals (Stone Age - Late Iron Age). In: Security archaeological research in the Middle Urals vol. 1. Yekaterinburg: Publishing House 'Yekaterinburg', pp. 61–69 (1997).
198. Kuznetsov, P. F. The emergence of Bronze Age chariots in eastern Europe. *Antiquity* **80**, 638–645 (2006).
199. Kuznetsov, P. F. Dating of the monument at Repin Khutor and cultural and related materials of the Early Bronze Age of the steppe zone of Eastern Europe. *Russian Arc* **1**, 13–21(2013).
200. Gugalinskaya, L. A. *et al.* Paleoecology of soil formation on final Paleolithic settlement Sholma-1 (Cis-Volga Upland, Chuvash Plateau). *Bull Commission Study Quat* **70**, 45–58 (2010).
201. Berezina, N. Sites of Final Paleolithic and Mesolithic from the Chuvash Volga Region: the Issue of Cultural Interpretation. *Povolzhskaya Arkheologiya* (The Volga River Region Archaeology), 190–210 (2017).
202. Malov, N. M. & Filipchenko, V. V. Monuments of the catacomb culture of the Lower Volga. *Archaeological News* **4**, 52–62 (1995).
203. Malov, N. M. & Kosintsev, P. A. Settlement of the Volo-Don attacking bomb culture of Sosnovka I from the Saratov Volga. *Archeology of the East European Steppe* **8**, 78–94 (2010).
204. Morgunova, N. L. et al. Turganikskoye poseleniye v Orenburgskoy oblasti. [The Turganik settlement in the Orenburg region]. Orenburg: OGAU Publishing (Orenburg, 2017).
205. Reinhold, S. Kislovodsk, Russische Föderation: Spätbronzezeitliche Fundplätze im kaukasischen Hochgebirge. *e-Forschungsberichte* **2**, 82–88 (2017).
206. Kantorovich, A. R. Obrazno-syuzhetnyy repertuar vostochnoyevropeyskogo skifskogo zverinogo stilya: printsipy i rezultaty klassifitsirovaniya i kodirovaniya. Drevnosti. Issledovaniya. Problemy. Sbornik statey v chest, pp.195–223 (2018).
207. Mikhailova, O. V. & Kuzmina, O. V. New monuments of the Bronze Age in the Samara Volga. In: Protection and study of historical and cultural monuments in the Samara region. Samara, pp. 98–141 (1999).
208. Bochkarev, V. S. & Kuznetsov, P. F. Zhelobchatyie psalii epohi pozdney bronzyi evraziyskih stepey. [Fluted cheek-pieces from the Eurasian steppes under the late Bronze Ages] Koni, kolesnitsyi i kolesnichie stepey Evrazii. [Horses, chariots and chariot' drivers of Eurasian steppes]. In: *Horses, Chariots, and Chariot Drivers of the Eurasian Steppes*. Russian Academy of Sciences - Ural Branch. Yekaterinburg, pp. 257–291 (2010).
209. Vybornov, A., Kosintsev, P. & Kulkova, M. The origin of farming in the Lower Volga Region. *Documenta Praehistorica* **42**, 67–75 (2015).
210. Vybornov, A. et al. Diet and Chronology of Neolithic-Eneolithic Cultures (from 6500 to 4700 cal BC) in the Lower Volga Basin. *Radiocarbon* **60**, 1597–1610 (2018).
211. Bachura, O. P., Kisagulov, A. V. & Kosintsev, P. A. Large mammal fauna of the Late Pleistocene from the Verkhnegubakhinskaya cave (Perm Preduralye). *Fauna of the Urals and Siberia* **1**, 144–152 (2016).
212. Nikolskiy, P.A. *et al.* Implications of the discovery of a stag-moose (Cervalces sp., Cervidae, Mammalia) skull with different-sized antler beams. *Zool J* **98** 1177-1185 (2019).
213. Pitulko, V.V. *et al*. The Yana RHS Site: Humans in the Arctic before the Last Glaciation. *Science* **303**, 52–56 (2004).

214. Pitulko, V., Pavlova, E., Nikolskiy, P. Revising the archaeological record of the Upper Pleistocene Arctic Siberia: Human dispersal and adaptations in MIS 3 and 2. *Quat Sci Rev* **165**, 127-148 (2017).
215. Boeskorov, G. G. *et al.* Preliminary analyses of the frozen mummies of mammoth (*Mammuthus primigenius*), bison (*Bison priscus*) and horse (*Equus sp.*) from the Yana-Indigirka Lowland, Yakutia, Russia. *Integr Zool* **9**, 471–480 (2014).
216. Boeskorov, G. G. et al. A study of a frozen mummy of a wild horse from the Holocene of Yakutia, East Siberia, Russia. *Mamm Res* **63**, 1-8 (2018).
217. Abad, Ò. E., i Garra, A. M. & Bieto, E. T. Cantorella (Maldà, Urgell), un nou assentament a l'aire lliure del neolític final-calcolític i del bronze ple a la vall del Corb. Tribuna d'Arqueologia 2011–2012 (2011).
218. Olària, C. Cova Fosca: un asentamiento meso-neolítico de cazadores y pastores en la serranía del Alto Maestrazgo. Diputación de Castellón. Servicio de Arqueologia (1988).
219. Olària, C. El Parque de la Gasulla (Ares del Maestre, Castellón): un ensayo de interpretación para un territorio con testimonios rupestres. *Quaderns de Prehistòria i Arqueologia de Castelló* **30**, 11-32 (2012).
220. Olària, C. Nuevas dataciones de C-14 para el Neolítico mediterráneo peninsular. *Quaderns de Prehistòria i Arqueologia de Castelló* **21**, 27-34 (2000).
221. Llorente Rodríguez, L. The hares from Cova Fosca (Castellón, Spain). *Archaeofauna* **19**, 59-97 (2010).
222. Lira, J. et al. Ancient DNA reveals traces of Iberian Neolithic and Bronze Age lineages in modern Iberian horses. *Mol Ecol* 19, 64–78 (2010).
223. Llorente-Rodríguez, L., Ruíz-García, J.-J. & Morales-Muñiz, A. Herders or hunters? Discriminating butchery practices through phalanx breakage patterns at Cova Fosca (Castellón, Spain). *Quat Int* **330**, 61–71 (2014).
224. Llorente Rodríguez, L. Cova Fosca (Ares del Maestrat, Castellón): Arqueozoología de la Revolución Neolítica en la fachada levantina de Iberia. PhD Thesis. Madrid: Universidad Autónoma de Madrid (2015).
225. Liesau, C. Arqueozoología del caballo en la antigua Iberia. *Gladius* **25**, 187–206 (2005).
226. Celestino, S. & Rodríguez González, E. Un espacio para el sacrificio: el patio del yacimiento tartésico de Casas del Turuñuelo (Guareña, Badajoz). *Complutum* **30**, 343-366 (2019).
227. Marín-Aguilera B, Rodríguez-González E, Celestino S, & Gleba M. Dressing the sacrifice: textiles, textile production and the sacrificial economy at Casas del Turuñuelo in fifth-century BC Iberia. *Antiquity* **93**, 933-953 (2019).
228. Nieto Espinet, A. Seguint les traces de la transhumància. Aproximació teòrica a partir dels resultats arqueozoològics de la fortalesa dels Vilars (Arbeca, Garrigues). *Revista d'arqueologia de Ponent* **26**, 11-34 (2016).
229. Nieto, A., Gardeisen, A., Junyent, E. & López, J. B. Inhumations de foetus d'équidés dans la forteresse du premier âge du Fer de Els Vilars (Arbeca, Catalogne). In: *Histoires d'équidés : des textes, des images, des os*. Gardeisen, A. eds. Montpellier, France, pp. 125–148 (2010).
230. Ortiz, N. S., Pàmies, D. & Moreno, I. De Sigarra a Prats de Segarra: noves descobertes arqueològiques al Municipium Sigarrense (els Prats de Rei, Anoia) entre l'ibèric antic i l'edat mitjana. Tribuna d'Arqueologia 2013-2014. Servei d'Arqueologia I Paleontologia. Barcelona (2016).
231. Sanmartí, J. *et al.* Filling Gaps in the Protohistory of the Eastern Maghreb: The Althiburos Archaeological Project (El Kef, Tunisia). *J Afric Arc* **10**, 21–44 (2012).
232. Kallala, N., Belarte Franco, M. C. & Others. Althiburos II. La fouille dans l'aire du capitole et dans la nécropole méridionale: études. Documenta 28, Institut Català d'Arqueologia Clàssica. Tarragona, pp. 550 (2016).
233. Valenzuela-Lamas, S. Alimentation et élevage à Althiburos à partir des restes fauniques. In: Kallala, N.; Sanmartí, J. (Dirs.); Belarte, M.C. (Ed.): *Althiburos II. L'aire du capitole et la nécropole méridionale: études*. Documenta 28, Institut Català d'Arqueologia Clàssica. Tarragona, pp. 421-448 (2016).

234. Arbuckle, B. S. Pastoralism, Provisioning, and Power at Bronze Age Acemhöyük, Turkey. *Am Anthropol* **114**, 462–476 (2012).
235. Arbuckle, B. S. Chalcolithic Caprines, Dark Age Dairy, and Byzantine Beef: A First Look at Animal Exploitation at Middle and Late Holocene Çadır Höyük, North Central Turkey. *Anatolica* **35**, 179–224 (2009).
236. Steadman, S. R. *et al.* Recent discoveries (2015-2016) at Cadir Hoyuk on the north central plateau. *Anatolica* **43**, 203–250 (2017).
237. Steadman, S. R., McMahon, G. & Ross, J. C. Chalcolithic, Hittite/Iron Age, and Byzantine investigations Çadır Höyük: The 2017-2018 Seasons. In: The Archaeology of Anatolia: Recent Discoveries (2017-2018), Vol. III. Steadman, S. R. & McMahon, G. eds. Newcastle upon Tyne: Cambridge Scholars Publishing, pp. 307 (2019).
238. Benecke, N. On the beginning of horse husbandry in the southern Balkan Peninsula- the horse bones from Kirklareli-Kanhgecit (Turkish Thrace). In: Equids in Time and Space: Papers in Honour of Véra Eisenmann. Oxford, Oxbow Books, pp. 240 (2006).
239. Özdoğan, M. & Parzinger, H. Die frühbronzezeitliche Siedlung von Kanlıgeçit bei Kırklareli. Ostthrakien während des 3. Jahrtausends v. Chr. im Spannungsfeld von anatolischer und balkanischer Kulturentwicklung. Archäologie in Eurasien 27, Studien im Thrakien-Marmara-Raum 3. Darmstadt: Philipp von Zabern (2012).
240. Silistreli, U. Les fouilles de Köşk Höyük. In: Anatolia and the ancient Near East: Studies in honor of Tahsin Özgüç. Emre, K., Mellink, M., Hrouda, B. & Özgüç, N. eds. Ankara, pp. 461–463 (1989)
241. Öztan, A. Köşk Höyük: Nigde--Bor Ovası'nda bir Neolitik yerlesim. In: Turkiye'de Neolitik Donem: Yeni kazılar, yeni bulgular. Arkeoloji ve Sanat Yayınları, Istanbul, pp. 223–236 (2007).
242. Öztan, A. Köşk Höyük: Anadolu Arkeolojisine Yeni Katkılar. *TUBA-AR* **5**, 55-69 (2016).
243. Kotova, N. S. & Tuboltsev, O. V. New settlements of the Neolithic-Eneolithic period at Melitopol. Eurasia antiqua. *Zeitschrift für Archäologie Eurasiens* **2**, 29–58 (1996).
244. Kotova, N. S. Neolithization in Ukraine. *BAR International Series* **1109**, pp. 300 (2003).
245. Kotova, N. S. Early Eneolithic in the Pontic Steppes. *BAR International Series*, pp. 314 (2008).
246. Clark, P., Shand, G. & Weekes, J. Chalk Hill: Neolithic and Bronze Age Discoveries at Ramsgate, Kent. Sidestone Press, pp. 275 (2019).
247. Higham, T. *et al.* The earliest evidence for anatomically modern humans in northwestern Europe. *Nature* **479**, 521–524 (2011).
248. Jacobi, R. & Higham, T. Radiocarbon Dating of the Later Upper Palaeolithic Human Occupation of Kent's Cavern, Devon, England. In: New AMS Results. Understanding the Past: Papers Offered to Stefan K. Kozlowski. Warsaw: Center for Research on the Antiquity of Southeastern Europe, pp. 137–154 (2009).
249. Pinhasi, R. *et al.* Optimal Ancient DNA Yields from the Inner Ear Part of the Human Petrous Bone. *PLoS ONE* **10**, e0129102 (2015).
250. Yang, D. Y., Eng, B., Waye, J. S., Dudar, J. C. & Saunders, S. R. Technical note: improved DNA extraction from ancient bones using silica-based spin columns. *Am J Phys Anthropol* **105**, 539–543 (1998).
251. Gamba, C. *et al.* Comparing the performance of three ancient DNA extraction methods for high-throughput sequencing. *Mol Ecol Resour* **16**, 459–469 (2016).
252. Drummond, A. J., Ho, S. Y. W., Phillips, M. J. & Rambaut, A. Relaxed Phylogenetics and Dating with Confidence. *PLoS Biol* **4**, (2006).
253. Chen, L., Liu, P., Evans, T. C. & Ettwiller, L. M. DNA damage is a pervasive cause of sequencing errors, directly confounding variant identification. *Science* **355**, 752–756 (2017).
254. Vieira, F. G., Lassalle, F., Korneliussen, T. S. & Fumagalli, M. Improving the estimation of genetic distances from Next-Generation Sequencing data. *Biol J Linn Soc* **117**, 139–149 (2016).
255. Linderholm, A. & Larson, G. The role of humans in facilitating and sustaining coat colour variation in domestic animals. *Semin Cell Dev Biol* **24**, 587–593 (2013).
256. Ludwig, A. et al. Coat color variation at the beginning of horse domestication. *Science* **324**, 485 (2009).

257. Bellone, R. R. *et al.* Evidence for a retroviral insertion in TRPM1 as the cause of congenital stationary night blindness and leopard complex spotting in the horse. *PLoS ONE* **8**, e78280 (2013).
258. Bartholomae, Chr. *Altiranisches Wörterbuch*. Karl J. Trübner: Strassburg (1904).
259. Mayrhofer, M. Welches Material aus dem Indo-Arischen von Mitanni verbleibt für eine selektive Darstellung? In: E. Neu (ed.), *Investigationes philologicae et comparativae: Gedenkschrift für Heinz Kronasser*. Wiesbaden: Harrassowitz, pp. 72–90 (1982).
260. Mayrhofer, M. *Etymologisches Wörterbuch des Altindoarischen*. Heidelberg: Carl Winter (1992-2001).
261. Meid, W. Die Terminologie von Pferd und Wagen im Indogermanischen. In: B. Hänsel, Stefan Zimmer (ed.), *Indogermanen und das Pferd*. Budapest: Archaeolingua Alapitvany, pp. 53–66 (1994).
262. Parvulescu, A. The Indo-European horse: A linguistic reconstruction. *Word* **44**: 69–76 (1993).
263. Pokorny, J. *Indogermanisches etymologisches Woŕterbuch*. Francke. Bern (1959–1969).
264. Lubotsky, A.M. Subm. Indo-European and Indo-Iranian wagon terminology and the date of the Indo-Iranian split. In: Kristian Kristiansen, Guus Kroonen & Eske Willerslev, *When Archaeology Meets Linguistics and Genetics. Towards a new European Prehistory*. Oxford University Press, *in press*.
