# Supplementary material for: The origins and spread of domestic horses from the Western Eurasian steppes
Source: Nature. 2021 Oct 20;598(7882):634–40. doi: 10.1038/s41586-021-04018-9 (PMC8550961; doi:10.1038/s41586-021-04018-9)
Supplement: Supplementary file 2 — Reporting Summary [file 41586_2021_4018_MOESM2_ESM.pdf]

nature portfolio

Corresponding author(s): Ludovic ORLANDO

Last updated by author(s): Aug 12, 2021

Reporting Summary

Nature Portfolio wishes to improve the reproducibility of the work that we publish. This form provides structure for consistency and transparency in reporting. For further information on Nature Portfolio policies, see our Editorial Policies and the Editorial Policy Checklist.

Statistics

For all statistical analyses, confirm that the following items are present in the figure legend, table legend, main text, or Methods section.

n/a	Confirmed	
☐	☒	The exact sample size (*n*) for each experimental group/condition, given as a discrete number and unit of measurement
☐	☒	A statement on whether measurements were taken from distinct samples or whether the same sample was measured repeatedly
☒	☐	The statistical test(s) used AND whether they are one- or two-sided<br>*Only common tests should be described solely by name; describe more complex techniques in the Methods section.*
☐	☒	A description of all covariates tested
☐	☒	A description of any assumptions or corrections, such as tests of normality and adjustment for multiple comparisons
☐	☒	A full description of the statistical parameters including central tendency (e.g. means) or other basic estimates (e.g. regression coefficient) AND variation (e.g. standard deviation) or associated estimates of uncertainty (e.g. confidence intervals)
☒	☐	For null hypothesis testing, the test statistic (e.g. *F*, *t*, *r*) with confidence intervals, effect sizes, degrees of freedom and *P* value noted<br>*Give P values as exact values whenever suitable.*
☐	☒	For Bayesian analysis, information on the choice of priors and Markov chain Monte Carlo settings
☐	☒	For hierarchical and complex designs, identification of the appropriate level for tests and full reporting of outcomes
☐	☒	Estimates of effect sizes (e.g. Cohen's *d*, Pearson's *r*), indicating how they were calculated

Our web collection on statistics for biologists contains articles on many of the points above.

Software and code

Policy information about availability of computer code

Data collection	All metadata pertaining to the experimental work underlying ancient DNA characterization at CAGT is managed through the open-source CASCADE laboratory information management system (see Dolle et al. Frontiers Ecol Evol 2020).
Data analysis	The Struct-f4 software is available without restriction on Bitbucket at https://bitbucket.org/plibradosanz/structf4/src/master/, together with a companion manual providing installation and running instructions. All other analyses relied on available software, fully referenced in the manuscript, including:<br>-AdapterRemoval2 (version 2.3.0),<br>-Paleomix (version 1.2.13.2),<br>-mapDamage2 (version 2.0.8),<br>-ANGSD (version 0.933-86-g3fefdc4, htslib: 1.10.2-106-g9c35744) ,<br>-PMDtools (version 0.60),<br>-mafft (version v7.407),<br>-RAxML (version 8.2.11),<br>-BEAST (version 2.5.1),<br>-ModelGenerator (version 0.85),<br>-Tracer (version 1.7.2),<br>-IQtree (version 1.6.12),<br>-FastME (version 2.1.4),<br>-bed2diffs_v1 from EEMS (built with Eigen version 3.2.2 and Boost version 1_57, and using rEEMSplots version 0.0.0.9000),<br>-the ape R package (version 5.5),<br>-Plink (version v1.9),<br>-qpAdm (version 7.0),<br>-OrientAGraph (version 1.0),<br>-fastsimcoal2 (version 2.6.0.3),

-the mapplots package (version 1.5.1),
-the ncf R package (version 1.2-9),
-the geosphere R package (version 1.5-10) ,
-the GeoRange R package (version 0.1.0),
-LOCATOR (version 1.2), and;
-the GViz R package (version 1.36.2).

For manuscripts utilizing custom algorithms or software that are central to the research but not yet described in published literature, software must be made available to editors and reviewers. We strongly encourage code deposition in a community repository (e.g. GitHub). See the Nature Portfolio guidelines for submitting code & software for further information.

Data

Policy information about availability of data

All manuscripts must include a data availability statement. This statement should provide the following information, where applicable:

- Accession codes, unique identifiers, or web links for publicly available datasets
- A description of any restrictions on data availability
- For clinical datasets or third party data, please ensure that the statement adheres to our policy

All collapsed and paired-end sequence data for samples sequenced in this study are available in compressed fastq format through the European Nucleotide Archive under accession number PRJEB44430, together with rescaled and trimmed bam sequence alignments against both the nuclear and mitochondrial horse reference genomes. Previously published ancient data used in this study are available under accession numbers PRJEB7537, PRJEB10098, PRJEB10854, PRJEB22390 and PRJEB31613, and detailed in Supplementary Table 1. The genomes of ten modern horses, publicly available, were also accessed as indicated in their corresponding original publications (see Jonsson et al. PNAS 2014, Der Sarkissian et al. Curr Biol 2015, Renaud et al. 2018, Jagannathan et al. 2019, Andersson et al. 2012).

Field-specific reporting

Please select the one below that is the best fit for your research. If you are not sure, read the appropriate sections before making your selection.

☐ Life sciences ☐ Behavioural & social sciences ☒ Ecological, evolutionary & environmental sciences

For a reference copy of the document with all sections, see nature.com/documents/nr-reporting-summary-flat.pdf

Ecological, evolutionary & environmental sciences study design

All studies must disclose on these points even when the disclosure is negative.

Study description	We have sequenced 264 ancient horse genomes, including from regions and/or time periods that remained uncharacterized at the genetic level. Additionally, we complemented the data previously generated for nine ancient horses and included a total of ten modern horse genomes, selected to represent a whole diversity range of breeds/populations. We applied procedures aimed at minimizing the impact of post-mortem DNA damage on sequence quality and identified a total of 10M+ high-quality polymorphic sites in our data set. For sites of critical importance, ancient genome variation was characterized from more than a single genome, providing individual replicates of the signatures identified. Metadata considered in the analyses included the GPS coordinates of the excavation sites and the age of the samples analyzed, most often assessed from radiocarbon dating. The data set gathered helped solved long-standing controversies about horse domestication.
Research sample	Ancient horse remains (Equus ferus caballus) were collected and screened by sequencing following DNA extraction and next-generation DNA library preparation in state-of-the-art ancient DNA facilities. Shallow DNA sequencing helped measure DNA preservation levels so as to identify those specimens for which whole genome sequences could be characterized by means of shotgun DNA sequencing. We mainly focused on the time period spanning the first to fourth millennium BCE (Before Common Era), as it encompassed the whole time frame of horse domestication. We, however, also included older samples so as to characterize the pre-domestication population structure. Finally, we also sequenced the genome of one historical 'Tarpan' specimen, given the contentious status of this population regarding horse domestication. Archaeological sites, individual specimens as well as their respective radiocarbon dates and sex assignments (as inferred from DNA data) are provided in Supplementary Table 1. Supplementary Methods provide additional information on each archaeological site.
Sampling strategy	Sampling was conditioned by the availability of ancient remains. In order to cover the whole temporal and geographic range relevant for horse domestication, we have gathered together an extensive team of archaeologists and curators in charge of material collection from across Eurasia and North-Africa, and with full authority to undertake research-based activity on such material. The resulting data represents a large collection of 264 ancient genomes. Combined with a selection of genomes previously published, both modern and ancient, they provided adequate data and statistical power for statistical testing. The robustness of our analyses was assessed through a range of appropriate statistical methods, including bootstrapping, Maximum Likelihood, replicates and formal statistical tests. Sampling procedures were aimed at minimizing destruction, and were focused, whenever possible, on osseous remains such as petrosal bones, that are generally associated with better average DNA preservation.
Data collection	A majority of the remains consisted of loose petrosal bones and teeth that lost connection with their original skulls. These were either directly shipped to Ludovic Orlando by the archaeologists and/or curators in charge, or delivered in person to him (as he visited his collaborators, or when his collaborators visited his laboratory). Sampling for DNA extraction and radiocarbon dating were performed in the ancient DNA facilities of the CAGT laboratory using appropriate drilling instruments under flow-hoods and in an environment with filtered, positive air pressure. Routine procedures at CAGT are aimed at minimizing both destruction and contamination.

Timing and spatial scale	All archaeological remains investigated in this study are described in Supplementary Table 1 and have been collected, mostly from 2017 (although the earliest was collected in 1995). A study that was then ongoing in our laboratory suggested that horse husbandry at Botai did not give rise to modern domestic horses, which implied that another domestication centre was yet to be found. As the Botai culture is located in the second half of the third millennium BCE of central Asia, we mainly decided to focus our attention on the following 1000 years, but also extended sampling to other regions, including those previously described as potential domestication centres. Both due to the complexity of the horse population genetic structure at the time, and the relatively limited abundance of horse archaeological remains during the third millennium BCE, we extended sampling to prior the third millennium in order to increase resolution and gain insights on those lineages pre-dating domestication. Finally, the discovery that a massive population turnover took place from the late third to early second millennium BCE led us characterize the second millennium BCE more extensively at the genetic level. Overall, over 2,000 archaeological remains have been collected since 2017. They have been screened for DNA and subjected to shotgun DNA sequencing whenever showing sufficient DNA quality, following a routine data production pipeline at CAGT. Decisions on which locations and time periods should be given priority or added to our data set were made as the project made progress following preliminary analyses of the data collected, and re-assessed on approximately a monthly basis. The genetic data analyzed in this study correspond to a main data freeze that was done in July 2020, and supplemented with additional data from November 2020.
Data exclusions	No data generated in this study were excluded from our analyses. Following standard procedures in ancient DNA research, transition substitutions were masked from most analyses in order to limit the noise added by post-mortem DNA damage, largely inflating DNA sequencing rates. We limited our analyses to only a fraction of horse genomes previously reported in order to (1) avoid technical batch effects that may have structured the data due to the different DNA library construction and sequencing technologies used, (2) reduce computational burden and (3) only those time and/or geographic regions that were relevant for the present study.
Reproducibility	The quality of the genome sequences was validated by calculating their respective sequencing error rates. Data uncertainty was accounted for in our analyses (e.g. base qualities) to assess robustness. Genome originating from the same stratigraphic layers in a given site carried similar genetic information.
Randomization	Groups were defined to reflect ancient horse populations, eg horses from Iberia carrying similar genome variation were clustered together, and considered to form an ancient Iberian population. Such clustering was based on phylogenetic inference and on the temporal and geographic provenance of the specimens.
Blinding	Blinding was not applicable to this study since the geographic and temporal metadata associated with each sample was key to carry research (and prioritize those areas worth further sampling and investigations). However, computational analyses were first carried out at the individual level, before groups/populations were defined.

Did the study involve field work? ☐ Yes ☒ No

Reporting for specific materials, systems and methods

We require information from authors about some types of materials, experimental systems and methods used in many studies. Here, indicate whether each material, system or method listed is relevant to your study. If you are not sure if a list item applies to your research, read the appropriate section before selecting a response.

Materials & experimental systems

n/a	Involved in the study	
☒	☐	Antibodies
☒	☐	Eukaryotic cell lines
☐	☒	Palaeontology and archaeology
☒	☐	Animals and other organisms
☒	☐	Human research participants
☒	☐	Clinical data
☒	☐	Dual use research of concern

Methods

n/a	Involved in the study	
☒	☐	ChIP-seq
☒	☐	Flow cytometry
☒	☐	MRI-based neuroimaging

Palaeontology and Archaeology

Specimen provenance	The samples that were analyzed in this study were collected from a range of environmental conditions, spanning Northern African to Siberian excavation conditions. As this involved sampling from across Eurasia and different procedures between countries and institutions, key contact persons were identified in each country so as to access relevant material and coordinate legal authorization to sample material for DNA analysis and radiocarbon dating. Samples were collected with permission from the organizations holding the collections and documented through official agreement letters provided by the named archaeologists and/or curators and/or directors of relevant institutions, all named below. Official agreements to share material for partially destructive research were sent to Ludovic Orlando in the form of authorization letters, as part of the ethical framework established for the ERC PEGASUS Consolidator grant (681601). We sought every opportunity to access samples as part of collaborations with other research projects so as to both save resources and avoid double sampling, and, thus, ultimately minimize destruction. The archaeological sites where each individual specimen was excavated are listed in Supplementary Table 1. The exact locations of each remain are provided with full reference to the names of the archaeological sites and their GPS coordinates. The following list provides the sites and names of those collaborators (together with the head of their institution when necessary) who granted access to the corresponding material, with reference to letters and permits where appropriate:<br><br>-Kostenki 15 (layer 25183 (179), square 623/K-25), Medvezhiya (layer 34763 (7.2-7.4 square N-1, 4.4-4.6 square 15-n, 1.5-2.0 square

N3 and 5.0-5.2 square N1)): Dr. N. S. Chernetsov (collections of the Archaeological Institute of the Russian Academy of Sciences, Sankt-Petersburg; authorization letter nb 12505-2145/239),

-Tepe Hasanlu, Tepe Sagzabad: Dr. M. Mashkour, Dr. M. S. Salehi (Archaeology Institute of the University of Teheran, authorization letter nb 654-854), Dr. B. Omrani (Research Institute of the Iranian Cultural Heritage and Tourism, authorization letter nb 9810308),

-Aygurskiy: V. A. Babenko (Stavropol, excavation 'Nasledie' 2000-2001, license nb 2000-776 and nb 2001-776, exported in 2016 to the German Archaeological Institute, Berlin, Germany),

-Ullu: Dr. A.B. Belinskij (Stavropol, excavation 'Nasledie' & DAI Eurasia-Department 201, license nb 2013-633, exported in 2013),

-Tarpan from the Kherson Region: Dr. T. V. Kuznetsova (Department of Paleontology, Faculty of Geology, Moscow State University; collections of the Zoological Institute, Russian Academy of Sciences, Sankt-Petersburg, Russia; collection nb O.521),

-Yukaghir: Dr. A.V. Prokopiev (Geological Museum of the Diamond and Precious Metal Geology Institute, Siberian branch, Russian Academy of Sciences; letter nb 304-03-21-0443/503),

-Yana complex of sites, Divnogor'ye 9: Pr. V. Pitulko, Pr. A. A. Bessudnov, O. I. Boguslavskiy (Institute of Material Culture, Russian Academy of Sciences; projects 16-18-10265 and 21-18-00457 from the Russian Science Foundation, field permits 468 (2016), 738 (2017) 779 (2018); confirmation letter nb 14102/33-772.4-263),

-Algay, burial mound at Berezovaya Mountain, Krasnosamarskoe, Noviye Kluchi III, Oroshaemo I, Potapovka, Repin Khutor, Turganik, Ouren, Utevka VI, Uvarovka II, Varfolomeevka: Pr. P. Kuznetsov, and Rector Dr. O.D. Mochalov (collections from the Archaeological Laboratory at the Samara State University of Social Sciences and Education, Russia; confirmation letter nb 03-01-Myzea),

-Sosnovka, Sintashta, Bol'shekaraganskii, Kameennyi Ambar 5, Aleksandrovskoe IV, Serpievskaya, Nikolskaya, Sholma-1, Pershinskaya, Verkhnegubakhinskaya: Dr. M. G. Golovatin (Institute of Plant and Animal Ecology, Ural Branch of the Russian Academy of Sciences; confirmation letter nb 16353-2115/214),

-Semenovka 1: Pr. N. S. Kotova, Pr. A. B. Bujskikh (Institute of Archaeology, National Ukrainian Academy of Sciences, Kiev, Ukraine; confirmation letter nb 125/01-19-334),

-Ganga-Tsagaan-Ereg, Khantain-tov, Monostoy-Nuga, Ushkin-Uver, Arzhan-1, Chinge-Tey-I, Hyena's Lair, Novoilinka-III, Novoilinka-VI, Bijke-V, Choburak-I, Kuyum: Pr. A. Tishkin, Dr. I. I. Nazarov (Institute of History, Barnaul University; project 19-59-15001 co-funded by CNRS and the Russian Foundation for Basic Research),

-Morin Mort, Bor Shoroonii, Zeerdegchingiin Khoshuu, Zunii Gol, Zuunkhangai, Ulaan Tolgoi: Pr. J. Bayarsaikhan (collections from the National Museum of Mongolia, Ulaanbaatar; exported in 2015 and 2015 under research agreement nb 20150315), and Dr. W. Taylor (Fulbright US Student research award nb 34154234, National Geeographic Young Explorer’s grant nb 9713-15), National Science Foundation Doctoral Dissertation Improvement Grant nb 1522024),

-Burgast-1, Tatsyn Ereg: Dr. Associate Pr. G. Eregzen (collections of the Institute of Archaeology, Mongolian Academy of Sciences, Ulaanbaatar, Mongolia; confirmation letter nb 01/48),

-Pietrele, Dr. E. Nicolae; Căscioarele, Nandru Peștera Curată: Dr. A. Bălășescu (Bioarchaeology Department, Vasile Pârvan Institute of Archaeology, Romanian Academy),

-Cova Fosca: Pr. C. Rosa Olaria Puyoles (Catedra de Prehistoria, Universitat Jaume I, Castello Spain),

-Els Vilars, Cantorella, Sigarra, La Monédière: Dr. A. Nieto Espinet (History Department, University of Leida, Spain),

-Casas del Turuñuelo, Pr. S. Celestino (Instituto Arqueologia Merida, Spain; projects IB10131 and IB18060, both funded by Junta de Extremadura and European Regional Development Fund),

-Althiburos: Dr. Silvia Valenzuela-Lamas (Institucio Mila i Fontanals, Barcelona, Spain),

-Fengtai, Zambujal, Kirklareli-Kanligecit, Garbovat, Dunaújváros-Kosziderpadlás, Miciurin, Nitriansky Hrádok, Arzhan-II: Prof. Dr. N. Benecke (German Archaeological Institute, Berlin, Germany), and Prof. Dr. A. Ludwig (Leibniz-Institut fur Zoo- und Wildtierforschung, Berlin, Germany),

-Shilikty: Pr. A. T. Toleubayev, Associate Pr. R. S. Zhumatayev, Dean of Faculty M. S. Nogaibayeva (collections from the Department of Archaeology and Ethnology, Al-Farabi Kazakh National University, Almaty, Republic of Kazakhstan, confirmation letter nb 15-23-720); Krasnyi Yar, Botai: Pr. Viktor Zaibert, Dean of Faculty M. S. Nogaibayeva (collections from The Research Institutee ‘Archaeology and the Steppe Civilisations”, Al-Farabi Kazakh National University, confirmation letter nb 1523-602),

-Kent, Ashchisu, Novoil'inovskiy 2: Dr. V. G. Loman (collections of the Saryarka Archaeological Institute, E. A. Buketov Karaganda University, Karaganda, Republic of Kazakhstan; confirmation letter nb 4-21/151),

-Michurino I, Shiderty III, Borly, Borly 4: Dr. Viktor K. Merz, Acting Deputy Chairman of the Board for academic work P.O. Bykov (collections of the A. Kh. Margulan Joint Archaeological Research Centre Toraighyrov University, Republic of Kazakhstan; confirmation letter 107/1232),

-Belkaragai, Bestamak, Kozhai, Halvai: Pr. A. Logvin, Dr. I. Shevnina, Acting Vice-Rector on Science, Internationalization and Digitalization G. Shakamal (A. Baitursynov Kostanay Regional University, Kostanay, Republic of Kazakhstan; confirmation letter nb 15-30-09/1052),

-Kaposujlak-Vardomb: Pr. G. Kulcsar (head of Department of Prehistory, Institute of Archaeology, Research Centre for the Humanities, Hungarian Academy of Sciences Centre of Excellence; confirmation letter BTK KP/1557-1/2021),

-Bad Pirawarth: Dr. E. Pucher (Naturhistorisches Museum Wien); affiliated archaeological project researcher: Dr. C. Schwall (Department of Prehistory and Western Asian/Northeast African Archaeology, Austrian Archaeological Institute, Austrian Academy of Sciences; sample made available through project nb OAW Innovationsfonds 23100 – OAW4002),

-Březno u Loun (pit 629 ID 510), Černý Vůl (104/1975-1977 ID 250), Holubice (2/2005 ID 146), Litovice (it 118/2004 ID 352), Stránská skála, Tištín (pit 553/2002 ID 175312), Toušeň – Hradišťko (trench 5/1976 ID P3416), Vliněves (8805/2007 ID 4349), Tuchoměřice (36/2005 ID 428), Tuchoměřice – Kněžívka (73/2007 ID 1176): Mgr. J. Marik (Institute of Archaeology of the Czech Academy of Sciences, Prague, Czech Republic; confirmation letter nb ARVP-3464/2021),

-Gordinesti III burial ground, Gordinesti II-Sinca goala settlement: Dr. H. Shephard (Archaeological Institute of America, US), Dr. V. Ghilas (Institute of Cultural Heritage, Centre of Archaeology, Academy of Science of Moldova; confirmation letters nb 06/90-06/91 27.04.2017; custom declaration from May 4th 2017),

-Etiolles, Le Closeau, Tureau des Gardes: Dr. O. Bignon-Lau (ArScAn-UMR 7041 CNRS, Nanterre, France),

-Igue du Gral, co-directors of excavation campaigns at the site: Dr. J.-C. Castel, Dr. M. Boudadi-Maligne (Museum de Genève, Swizerland & PACEA, UMR 5199, University of Bordeaux, France),

-Tarquinia monumental complex: Pr. Dr. G. Bagnasco Gianni (Dipartimento di Beni Culturali E Ambientali Etruscologia, Universita Degli Studi di Milano, Italy),

-Miechow 3, Mozgawa, Pielgrzymowice, Kazimierza Wielka, Slawecinek: Pr. J. Wilczynski (collections of the Institute of Systematics and Evolution of Animals, Polish Academy of Sciences, Krakow, Poland); Mozgawa site: Dr. M. Moskal-del Hoyo excavation under project 2013/11/B/HS3/03822, funded by the Polish National Science Centre 2014-2017),

-Goyet: Dr. A. Folie (Paleontological Collections, Royal Belgian Institute of Natural Sciences, Brussels, Belgium; destructive analysis request DAD-2015-06),

-Hohler Stein bei Schwabthal: Dr. T. Seregély (collections of the Institute for Archaeology, Heritage Conservation Studies and Art History, University of Bamberg, Germany; excavation campaign from 2008),

-Acemhoyuk, Cadir Hoyuk, Kosk Hoyuk: Pr. Dr. A. Oztan, PR. G. McMahon, Pr. B. Arbuckle (Department of Anthropology, University of North Carolina at Chapel Hill; authorization from Aksaray, Nigde and Yozgat museums; permit references B.16.0.KVM.0.13.01.00.155.02 (YA.2011.52) 36712, B.16.0.KVM.4.51.00.01/160.02/483),

-Asva, Ridala: Pr. L. Lougas (Archaeological Research Collection, Tallin University; sampling report AI-PP-345, sample references AI 3799:467 and AI4261),

-Kent's cavern: B. Chandler (Collections and Engagement Manager, Torquay Museum, United Kingdom),

-Chalk Hill, Magor: Dr. R. Bendrey (School of History, Classics and Archaeology, collections from the University of Edinburgh, United Kingdom), and;

-Ginnerup: Dr. L. Klassen (collection from the Research Department, East Jutland Museum, Randers, Denmark).

The sample TP4 from Tachti Perda was made available from the remaining DNA material from one of our previous studies (Gaunitz et al. Science 2018).

Specimen deposition

The specimens are available upon direct request to the archaeologists and/or curators in charge. Photographs taken while processing the samples for DNA are also made available in Ludovic Orlando's laboratory.

Dating methods

A total of 207 archaeological samples have been radiocarbon dated at the Keck Carbon Cycle Accelerator Mass Spectrometry Laboratory, UC Irvine. Raw and calibrated radiocardon dates are provided in Supplementary Table 1 (IntCal20 calibration curve, OxCal Online).

☒ Tick this box to confirm that the raw and calibrated dates are available in the paper or in Supplementary Information.

Ethics oversight

No ethical oversight was required as the material was sampled following procedures aimed at minimizing destruction and damage, following discussion, supervision and agreement with those curators in charge of the collection. Additionally, DNA analyses aimed at sequencing whole genomes instead of a restricted list of target loci, which eliminates the need for additional sampling in the future. Combined, this complies to the 3R principles.

Note that full information on the approval of the study protocol must also be provided in the manuscript.
