# Supplementary figures and images for: The origins and spread of domestic horses from the Western Eurasian steppes
Source: Nature. 2021 Oct 20;598(7882):634–40. doi: 10.1038/s41586-021-04018-9 (PMC8550961; doi:10.1038/s41586-021-04018-9)

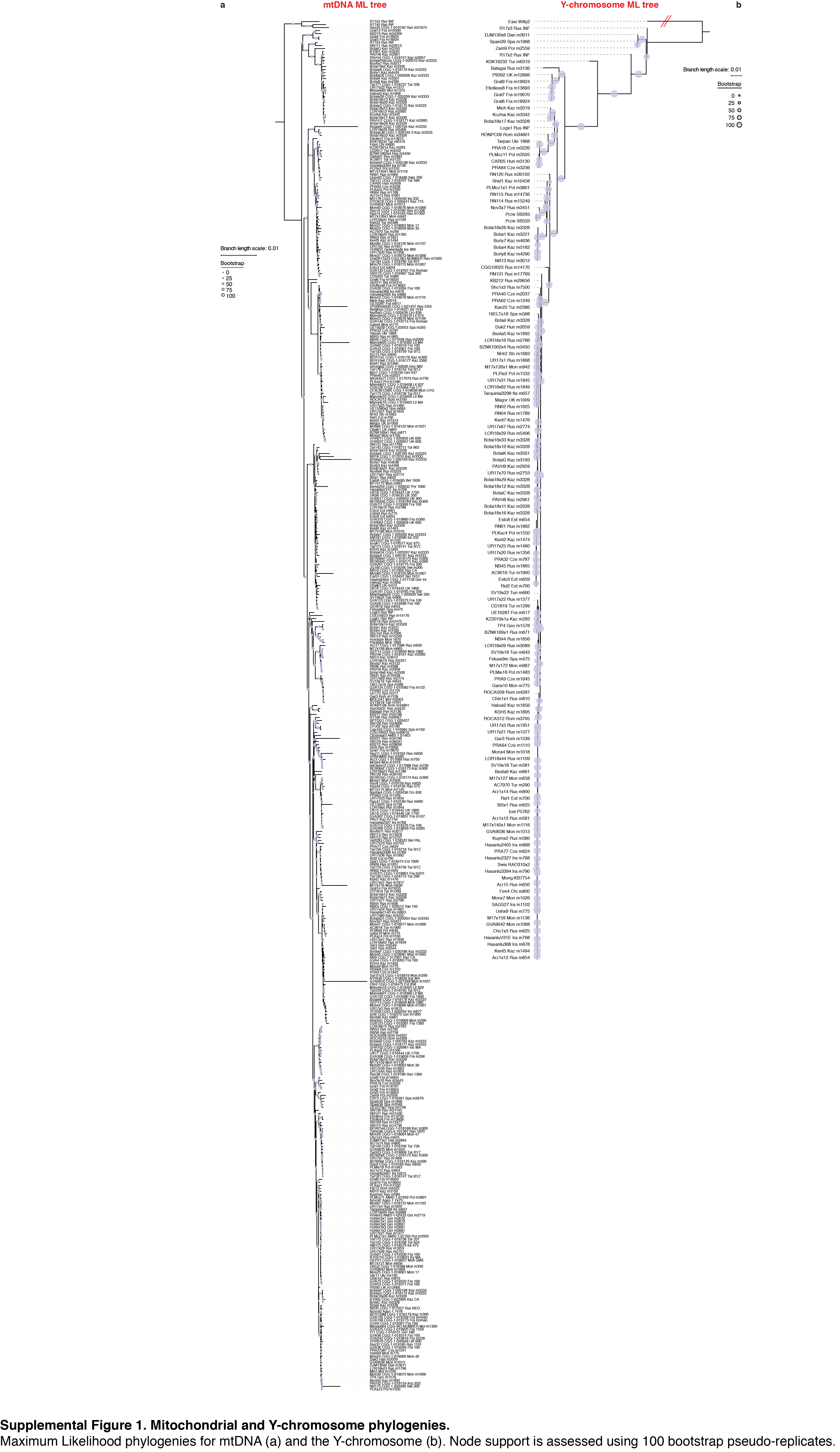

Supplement: Supplementary file 4 — Supplementary Fig. 1 Mitochondrial and Y-chromosome phylogenies This figure provides ML phylogenies mtDNA (a) and the Y-chromosome (b), with full sample labels. Node support is assessed using 100 bootstrap pseudo-replicates. [file 41586_2021_4018_MOESM4_ESM.jpg]
